# Identification of a tomato UDP-arabinosyltransferase for airborne volatile reception

**DOI:** 10.1038/s41467-023-36381-8

**Published:** 2023-02-08

**Authors:** Koichi Sugimoto, Eiichiro Ono, Tamaki Inaba, Takehiko Tsukahara, Kenji Matsui, Manabu Horikawa, Hiromi Toyonaga, Kohki Fujikawa, Tsukiho Osawa, Shunichi Homma, Yoshikazu Kiriiwa, Ippei Ohmura, Atsushi Miyagawa, Hatsuo Yamamura, Mikio Fujii, Rika Ozawa, Bunta Watanabe, Kenji Miura, Hiroshi Ezura, Toshiyuki Ohnishi, Junji Takabayashi

**Affiliations:** 1 Center for Ecological Research, Kyoto University, 2-509-3 Hirano, Otsu, Shiga 510-2113 Japan; 2 Tsukuba-Plant Innovation Research Center, University of Tsukuba, 1-1-1 Tennodai, Tsukuba, Ibaraki 305-8572 Japan; 3 Research Institute, Suntory Global Innovation Center Ltd, Suntory Foundation for Life Sciences, 8-1-1 Seika-dai, Seika, Souraku, Kyoto 619-0284 Japan; 4 Graduate School of Integrated Science and Technology, Shizuoka University, 836 Ohya, Suruga, Shizuoka 422-8529 Japan; 5 Graduate School of Sciences and Technology for Innovation, Yamaguchi University, 1677-1 Yoshida, Yamaguchi, 753-8515 Japan; 6 Bioorganic Research Institute, Suntory Foundation for Life Sciences, 8-1-1 Seika-dai, Seika, Souraku, Kyoto 619-0284 Japan; 7 Faculty of Agriculture, Shizuoka University, 836 Ohya, Suruga, Shizuoka 422-8529 Japan; 8 Agri-Intelligence Cultivation Institute, Shizuoka University, Nagoya, Suruga, Shizuoka 422-8529 Japan; 9 Graduate School of Engineering, Nagoya Institute of Technology, Gokiso-cho, Showa, Nagoya, 466-8555 Japan; 10 School of Pharmacy, International University of Health and Welfare, 2600-1 Kitakanemaru, Ohtawara, Tochigi 324-8501 Japan; 11 Institute for Chemical Research, Kyoto University, Gokasho, Uji, Kyoto 611-0011 Japan; 12 Research Institute of Green Science and Technology, Shizuoka University, 836 Ohya, Suruga, Shizuoka, 422-8529 Japan; 13 Institute for Tea Science, Shizuoka University, 836 Ohya, Suruga, Shizuoka, 422-8529 Japan; 14 Present Address: Chemistry Laboratory, The Jikei University School of Medicine, Kokuryo, Chofu, Tokyo 182-8570 Japan

**Keywords:** Plant ecology, Plant physiology

## Abstract

Volatiles from herbivore-infested plants function as a chemical warning of future herbivory for neighboring plants. (*Z*)-3-Hexenol emitted from tomato plants infested by common cutworms is taken up by uninfested plants and converted to (*Z*)-3-hexenyl β-vicianoside (HexVic). Here we show that a wild tomato species (*Solanum pennellii*) shows limited HexVic accumulation compared to a domesticated tomato species (*Solanum lycopersicum*) after (*Z*)-3-hexenol exposure. Common cutworms grow better on an introgression line containing an *S. pennellii* chromosome 11 segment that impairs HexVic accumulation, suggesting that (*Z*)-3-hexenol diglycosylation is involved in the defense of tomato against herbivory. We finally reveal that HexVic accumulation is genetically associated with a uridine diphosphate-glycosyltransferase (UGT) gene cluster that harbors *UGT91R1* on chromosome 11. Biochemical and transgenic analyses of UGT91R1 show that it preferentially catalyzes (*Z*)-3-hexenyl β-d-glucopyranoside arabinosylation to produce HexVic *in planta*.

## Abstract (web summary)

Volatiles from herbivore-infested plants can function as chemical warning signals to neighbouring plants. Here the authors show that a tomato UDP-glycosyltransferase can convert a volatile signal emitted by infested plants to promote plant defense.

## Introduction

Plant volatiles have various ecological functions including defense against herbivores and/or pathogens^1^. Herbivory-induced plant volatiles (HIPVs) are involved in indirect defense against herbivores as they attract carnivorous natural enemies of currently infesting herbivores^2–4^. HIPVs also mediate directional communication from infested plants (HIPV emitters) to uninfested plants (HIPV receivers)^5,6^. HIPVs are used by the receiver as infochemicals, indicating a possible future influx of herbivores from the emitter plants and the subsequent attack^2^. One of the intriguing questions in such plant–plant communication is how plants receive the volatiles.

One of the mechanisms of volatile reception in plant–plant communication is glycosylation. For example, in tomatoes, uninfested plants receive airborne (*Z*)-3-hexenol from plants infested with common cutworms (CCW, *Spodoptera litura*) and convert it to diglycosylated (*Z*)-3-hexenol, that is, (*Z*)-3-hexenyl-*O*-α-l-arabinopyranosyl-(1→6)-β-d-glucopyranoside [(*Z*)-3-hexenyl β-vicianoside (HexVic)], as a defense chemical against CCWs^7,8^. Plants can also glycosylate exogenously applied volatile alcohols^9^.

Plants generally store volatiles as hydrophilic mono- and di-glycosides in plant cells^10^. In tea leaves (*Camellia sinensis*), most endogenously synthesized volatile alcohols, such as terpene alcohols, green leaf alcohols, and phenylpropanoids, are primarily stored as the diglycoside β-primeveroside^11^. Tomato fruits of “non-smoky” cultivars accumulate volatiles as flavor precursors in hydrolysis-sensitive diglycosides during the early stages of development. In contrast, in the maturing stage, the diglycosides are additionally converted to hydrolysis-tolerant triglycosides, which no longer serve as flavor precursors^12^. Transgenic petunia plants (*Petunia hybrida*) overexpressing (*S*)-linalool synthase produce excess amounts of (*S*)-linalool and its glycoside, (*S*)-linalyl β-d-glucopyranoside^13^. Poplar plants (*Populus tremula* × *tremuloides*) overexpressing eugenol synthase accumulate eugenol and its mono- and di-glycosides^14^.

Genes involved in the glycosylation of endogenous volatiles in tomato fruit^15^ and tea leaves^11,16^ have been identified, supporting the notion that plants have developed glycosylation machinery to store endogenous volatiles as water-soluble glycosides in separate cell compartments. However, it remains unclear how plants convert exogenous volatiles to their glycosides. In this study, we aimed to identify the enzyme involved in HexVic biosynthesis and evaluate the contribution of volatile glycosylation in the defense against herbivores in tomatoes. We investigated the diversity in glycosylation of (*Z*)-3-hexenol among wild and domesticated tomato species. Using genetic and biochemical approaches, we unraveled how tomato plants convert exogenous (*Z*)-3-hexenol to its diglycoside for defense against herbivores.

## Results

### Variation in HexVic accumulation in the wild and a domesticated tomato species

We quantified HexVic content in 17 wild and 1 domesticated tomato species (*Solanum lycopersicum*) (listed in Table 1) after exposure to (*Z*)-3-hexenol to compare their ability to biosynthesize HexVic. Although nearly all exposed plants accumulated more HexVic than unexposed conspecifics, HexVic content varied between unexposed and exposed plants among the species (*p* < 0.05, Welch’s *t*-test for comparing unexposed and exposed plants, Table 1). One of the wild species, namely, *S. pennellii*, accumulated the lowest amount of HexVic (2.8% relative to *S. lycopersicum*). As genetic resources for *S. pennellii* are well established, we used this species in the subsequent experiments.

**Table 1:** HexVic content in various wild and domesticated tomato species

Species (accession number)		Control (μg·g^–1^ FW)	(*Z*)-3-Hexenol exposure (μg·g^–1^ FW)
*S. galapagense*	(LA0317)	0.72 ± 0.25	50.00 ± 6.87
*S. cheesmaniae*	(LA1041)	2.32 ± 0.12	43.22 ± 17.58
*S. lycopersicoides*	(LA2951)	0.65 ± 0.12	27.06 ± 6.88
*S. juglandifolium*	(LA0322)	0.93 ± 0.52	25.45 ± 0.98
*S. chilense*	(LA1930)	0.92 ± 0.35	24.94 ± 3.99
*S. huaylasense*	(LA1982)	0.48 ± 0.07	21.17 ± 9.45
*S. chmielewskii*	(LA1316)	1.02 ± 0.57	20.58 ± 8.49
*S. habrochaites*	(LA1777)	0.09 ± 0.04	15.16 ± 1.17
*S. peruvianum*	(TOMJPF00010)	0.30 ± 0.04	10.04 ± 0.87
*S. lycopersicum*	(TOMJPF00009)	0.10 ± 0.05	9.41 ± 1.93
*S. corneliomulleri*	(LA1973)	0.51 ± 0.20	7.87 ± 2.14
*S. pimpinellifolium*	(TOMJPF00011)	0.64 ± 0.23	7.48 ± 0.88
*S. ochranthum*	(LA2682)	0.28 ± 0.15	7.23 ± 1.86
*S. arcanum*	(LA1360)	0.16 ± 0.05	5.01 ± 0.97
*S. neorickii*	(LA1319)	0.12 ± 0.09	1.96 ± 0.20
*S. sitiens*	(LA1974)	0.11 ± 0.02	1.81 ± 1.57
*S. pennellii*	(TOMJPF00008)	ND	0.27 ± 0.15

HexVic content in the leaves of the indicated wild tomato species exposed or not exposed to (*Z*)-3-hexenol was analyzed. Data are mean ± SE from three independent experiments. The two-sided *p* values were estimated using Welch’s *t*-test for a comparison between control and (*Z*)-3-hexenol-exposured plants.

*ND* not detected, *FW* fresh weight.

To exclude the possibility that *S. pennellii* ecotype (accession no. TOMJPF00008) used in the above experiment would be distinct from other *S. pennellii* ecotypes in its ability to biosynthesize HexVic, we measured HexVic content in (*Z*)-3-hexenol-exposed *S. pennellii* plants collected from 17 natural habitats along a latitudinal gradient in Peru (Supplementary Fig. 1a). Despite variations among these accessions, their HexVic content was lower than that of *S. lycopersicum* (*p* < 0.05, Dunnett’s multiple comparison test) (Supplementary Fig. 1b). This result showed that *S. pennellii* is genetically impaired in terms of HexVic biosynthesis.

### Impaired HexVic production leads to susceptibility to common cutworms

**Fig. 1:**
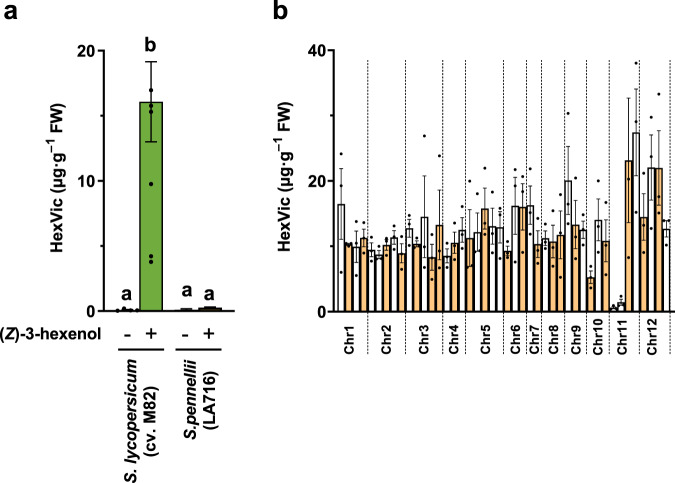
Chemical profiles of (*Z*)-3-hexenyl β-vicianoside (HexVic) in introgression lines of *S. pennellii*. **a** (*Z*)-3-Hexenyl β-vicianoside (HexVic) content in the leaves of parental species (*S. lycopersicum* cv. M82 and *S. pennellii* (LA0716)) of introgression lines (ILs) exposed (+) or not exposed (−) to (*Z*)-3-hexenol. Error bars indicate standard error of the mean from independent experiments (nine plants each of *S. lycopersicum* and *S. pennellii* not exposed to (*Z*)-3-hexenol. Four and five plants of *S. lycopersicum* and *S. pennellii* exposed to (*Z*)-3-hexenol, respectively). Statistical significance among species and treatments were determined using the one-way ANOVA with the Tukey’s multiple comparison tests, and significant differences are indicated by different characters (*p* < 0.05). **b** HexVic content in the leaves of (*Z*)-3-hexenol-exposed ILs. Error bars indicate standard error of the mean from three independent plants.

To assess the genetic basis of HexVic biosynthesis, we analyzed introgression lines (ILs) from a *S. lycopersicum* cv. M82 × *S. pennellii* (LA0716) cross^17^. Compared to M82 plants, LA0716 showed low HexVic accumulation after (*Z*)-3-hexenol exposure (approximately 7%, which was similar to the level in unexposed plants, *p* < 0.05, Tukey’s multiple comparison test; Fig. 1a). We measured HexVic content in 44 (*Z*)-3-hexenol-exposed ILs and found that IL11-1 and IL11-2 demonstrated impaired HexVic accumulation (Fig. 1b).

To evaluate the biological effect of HexVic deficiency under herbivory, the defense levels of IL11-1 and M82 plants (as a control) in response to challenge with CCWs were analyzed using larval weight. We did not use IL11-2 plants in the CCW feeding trials because their leaves showed pleiotropic phenotypes (Supplementary Fig. 2a); the leaves were softer and brighter light yellow–green than those of WT and IL11-1 plants. The weight of the second-instar CCWs on M82 plants was not significantly different from that of the second instars on IL11-1 plants 1 day after infestation (Supplementary Fig. 2b). However, after 7 days, the weight of the second instars on IL11-1 plants was higher than that of the second instars on M82 plants (Supplementary Fig. 2b). CCW-infested M82 plants substantially accumulated HexVic in their leaves compared to uninfested control plants, whereas IL11-1 plants did not (Supplementary Fig. 2c). As CCW infestation elicits (*Z*)-3-hexenol emission from tomato plants^7,18^, we reasoned that undamaged leaves of CCW-infested plants were also locally exposed to (*Z*)-3-hexenol and converted it to HexVic. This result showed that (1) the introgressed chromosome 11 segment derived from *S. pennellii* genome contains the genetic region responsible for impairment of HexVic and (2) susceptibility to CCWs in IL11-1 plants is potentially due to the impairment of HexVic accumulation after challenge.

### Screening of a candidate gene for HexVic biosynthesis in *S. lycopersicum*

**Fig. 2:**
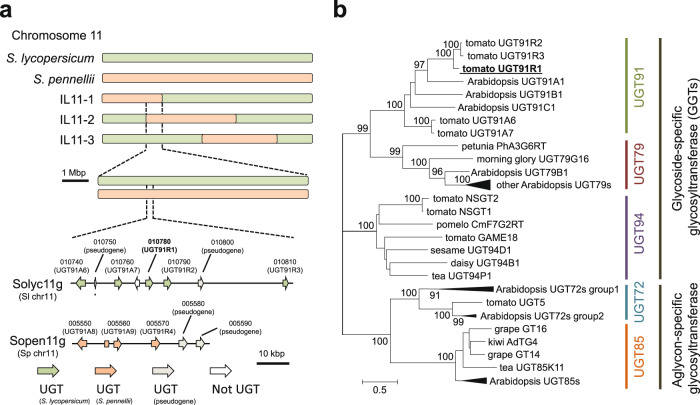
Genetic and phylogenetic tree analyses of glycosyltransferases located in overlap region of *S. pennellii* chromosome segment between IL11-1 and IL11-2. **a** Schematic diagram of tomato chromosome 11 around the locus of gene involved in HexVic biosynthesis. Green and orange bars represent chromosome segments derived from *S. lycopersicum* and *S. pennellii*, respectively. Introgression line (IL)11-1, IL11-2, and IL11-3 harbor different segments of *S. pennellii* chromosome. Overlap region of *S. pennellii* chromosome segment between IL11-1 and IL11-2 contains a gene cluster encoding putative glycosyltransferases in both *S. lycopersicum* and *S. pennellii*. Orientation of arrowhead indicates the orientation of glycosyltransferase genes, and the corresponding gene IDs (https://solgenomics.net/) are shown. **b** Phylogenetic tree of glycosyltransferases constructed from biochemically identified glycosyltransferases involved in specialized metabolites, including volatile glycosides. Sequences from various plant species were collected from GenBank protein database. As a reference for UGT families, *UGT* genes from *Arabidopsis* were used, except for UGT94, because this gene does not exist in *Arabidopsis*. A tree was constructed using the maximum-likelihood method with 1000 bootstrap replications.

Based on the segregated HexVic accumulation patterns, the gene(s) encoding the HexVic biosynthetic enzyme was assumed to be located in the overlap region between IL11-1 and IL11-2, which is functional in *S. lycopersicum*, but not in *S. pennellii* (Fig. 2a). The overlap region (approximately 8 Mbp) in *S. lycopersicum* genome contained approximately 290 genes (Supplementary Table 1), including a uridine diphosphate (UDP)-glycosyltransferase (UGT) cluster that comprises seven *UGT91* family genes (Fig. 2a and Supplementary Table 2). The UGT91 family proteins are classified into a functional phylogenetic group designated as glycoside-specific glycosyltransferases (GGTs), which specifically catalyze glycosylation at the sugar moiety of various glycosides (Fig. 2b and Supplementary Data 1)^19–23^. Two out of seven *UGT91* genes in the cluster (*Solyc11g010750* and *Solyc11g010800*) contained a premature stop codon; hence, they were predicted to encode a truncated protein and were, therefore, excluded from further analysis. *Solyc11g010780* was also predicted to encode a truncated protein based on data in the Solanaceae Genomics Network (https://solgenomics.net/). However, resequencing a full-length cDNA of *Solyc11g010780* from clone LEFL1056CC10 revealed that it encodes an intact UGT. Therefore, *Solyc11g010780* was included as a candidate together with the remaining four full-length genes.

To narrow down the candidate genes, we analyzed the correlation between gene expression and HexVic accumulation. Among the five candidates, three (*Solyc11g010760*, *Solyc11g010780*, and *Solyc11g010810*) were substantially expressed in the leaves (Supplementary Fig. 3a). Transcripts of *Solyc11g010780* were exclusively detected in the leaf tissues, whereas *Solyc11g010760* and *Solyc11g010810* were expressed in both leaves and green fruits (Supplementary Fig. 3a). The accumulation of HexVic after (*Z*)-3-hexenol exposure was evident in the leaves but not in immature and mature fruits of M82 plants. (Supplementary Fig. 3b). Based on the spatial correlation between transcript and HexVic accumulation, we concluded that *Solyc11g010780* (hereinafter termed *UGT91R1* according to its amino acid sequence^24^) was the most likely candidate gene involved in HexVic biosynthesis.

### Biochemical property of UGT91R1

**Fig. 3:**
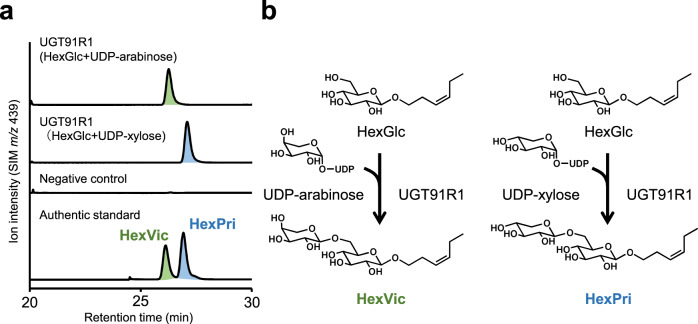
Biochemical characterization of tomato UGT91R1. **a** UGT91R1 catalyzes arabinosylation and xylosylation of HexGlc to produce HexVic and HexPri, respectively. **b** LC-MS analysis of enzymatic product of UGT91R1 compared with authentic standard (HexVic and HexPri). SIM, selected ion monitoring.

To test whether UGT91R1 derived from M82 plants catalyzes the arabinosylation of HexGlc into HexVic, we heterologously expressed UGT91R1 in *Escherichia coli* and performed in vitro enzymatic assays with recombinant UGT91R1, UDP-arabinose as a sugar donor, and HexGlc as a sugar acceptor. UGT91R1 produced a new peak with a retention time at 25.5 min (Fig. 3a). This peak was identical to that of the authentic HexVic, which was structurally determined to be arabinosylated at the C-6′ position of the glucoside moiety (Fig. 3b). Moreover, in the presence of UDP-xylose as a sugar donor, UGT91R1 converted HexGlc to (*Z*)-3-hexenyl-*O*-β-d-xylopyranosyl-(1→6)-β-d-glucopyranoside [(*Z*)-3-hexenyl β-primeveroside (HexPri)] (*t*_R_: 26.2 min) by xylosylation (Fig. 3a). In contrast, UGT91R1 was inert to any hexose-type sugar donor such as UDP-glucose, UDP-galactose, and UDP-glucuronic acid. These results showed that UGT91R1 transferred pentoses (arabinose and xylose) to the hydroxy group at the C-6′ position of the glucose moiety of HexGlc in vitro (Fig. 3b).

To further evaluate the substrate preference of UGT91R1 toward UDP-pentoses, the apparent steady-state kinetic parameters were determined for both UDP-arabinose and UDP-xylose as sugar donors (Table 2). The *K*_m_ values for HexGlc (5.9 μM and 7.9 μM with UDP-arabinose and UDP-xylose, respectively) showed comparable specificity. Moreover, the *k*_cat_ value for UDP-arabinose (0.33 s^−1^) was eight times more than that for UDP-xylose (0.04 s^−1^). Overall, the specificity constant (i.e., the *k*_cat_/*K*_m_ values) for UDP-arabinose was 11 times more than that for UDP-xylose (Table 2). These kinetic analyses suggested that UGT91R1 mediates HexVic accumulation in *S. lycopersicum* upon exposure to (*Z*)-3-hexenol by preferentially catalyzing the arabinosylation for HexGlc.

**Table 2:** Kinetic parameters of UGT91R1 and UGT91R4

Enzyme	Substrate fixed	Substrate variable	*K*_m_ (μM)	*k*_cat_ (s^−1^)	*k*_cat_/*K*_m_ (s^−1^mM^−1^)
UGT91R1	UDP-arabinose	HexGlc	5.9 ± 0.5	0.33 ± 0.009	57 ± 5
	UDP-xylose		7.9 ± 0.9	0.04 ± 0.001	5 ± 0.6
	HexGlc	UDP-arabinose	38 ± 4	1.9 ± 0.1	51 ± 5
		UDP-xylose	603 ± 75	1.8 ± 0.1	3 ± 0.4
UGT91R4	HexGlc	UDP-arabinose	23 ± 2	0.26 ± 0.01	11.2 ± 0.9

Data are presented as mean ± SD (*n* = 3).

### Metabolic changes in *UGT91R1-*knockout and -overexpressing tomatoes

**Fig. 4:**
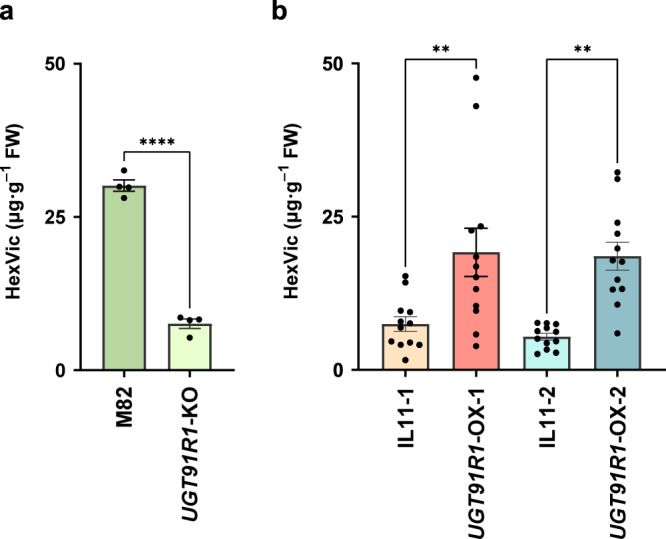
Metabolic changes in *UGT91R1*-knockout and -overexpressed tomatoes. **a** HexVic content in the leaves of *S. lycopersicum* cv. M82 and *UGT91R1*-knockout lines (*UGT91R1*-KO) exposed to (*Z*)-3-hexenol. Error bars indicate standard error of the mean from four independent plants. Statistical significance was determined using an unpaired two-tailed *t*-test, and reported as ****p < 0.0001. FW, fresh weight. **b** HexVic content in the leaves of *UGT91R1-*overexpressed IL11-1 and IL11-2 (*UGT91R1*-OX-1 and *UGT91R1*-OX-2, respectively) exposed to (*Z*)-3-hexenol, respectively. Error bars indicate standard error of the mean from 12 independent plants. FW, fresh weight. Data were analyzed using the one-way ANOVA with the Tukey’s test for multiple comparison. Statistical significance is reported as **p < 0.05.

**Fig. 5:**
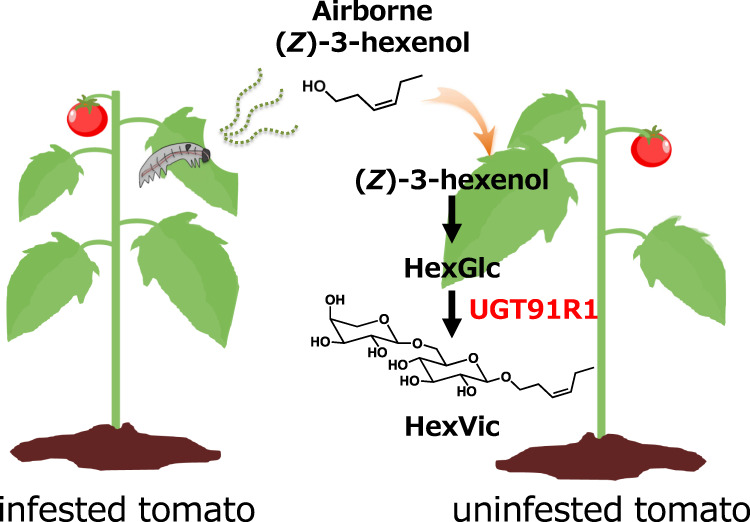
Role of (*Z*)-3-hexenol glycosylation in the chemical defense system against herbivory. Schematic representation of the roles of (*Z*)-3-hexenol glycosylation in the distal/future defense against herbivory.

To investigate the physiological function of UGT91R1 in tomatoes, we generated *UGT91R1*-knockout (*UGT91R1*-KO) tomatoes using genome editing and quantified endogenous HexVic accumulation in the transgenic tomatoes upon exposure to (*Z*)-3-hexenol (Supplementary Fig. 4). The amount of HexVic was reduced significantly to 25% of the amount in the respective wild-type tomato (HexVic; 7.6 ± 0.8 μg·g^–1^ FW in *UGT91R1*-KO and 30.1 ± 0.9 μg·g^–1^ FW in M82 plants, Fig. 4a). We also generated transgenic tomatoes that transiently overexpressed UGT91R1 (*UGT91R1*-OX-1 and *UGT91R1*-OX-2) in IL11-1 and IL11-2, which are intrinsically impaired in HexVic production. Notably, both *UGT91R1*-OX-1 and *UGT91R1*-OX-2 tomatoes significantly gained the ability to accumulate HexVic (19.2 ± 3.9 μg·g^–1^ FW in *UGT91R1*-OX-1 and 18.5 ± 2.3 μg·g^–1^ FW in *UGT91R1*-OX-2) compared to IL11-1 and IL11-2 (Fig. 4b). Collectively, UGT91R1 expression was strongly correlated with HexVic accumulation *in planta*. Remnants of HexVic in the *UGT91R1*-KO tomatoes suggest functionally redundant UGT genes other than *UGT91R1* in the UGT cluster result in the production of HexVic. In line with the biochemical properties of UGT91R1, these results showed that UGT91R1 is responsible for HexVic biosynthesis for airborne (*Z*)-3-hexenol *in planta* (Fig. 5). CCW feeding trials in *UGT91R1*-OX and *UGT91R1*-KO plants should be conducted in the future to gain further insights to understand the physiological and ecological functions of UGT91R1.

### Characterization of UGT91R1-related genes in *S. pennellii*

*Solanum pennellii* is intrinsically impaired in HexVic biosynthesis. However, *UGT91R1* homologs were present in *S. pennellii* genome. Based on a BLAST search against a publicly available genome sequence of *S. pennellii* (https://solgenomics.net/), we identified five homologs (Fig. 2a and Supplementary Table 2). *Sopen11g005550*, *Sopen11g005560*, *Sopen11g005570*, and *Sopen11g005580* exhibited high nucleotide similarity with *Solyc11g010740*, *Solyc11g010760*, *Solyc11g010780*, and *Solyc11g010810*, respectively (Supplementary Fig. 5a and Supplementary Data 2). Among these, Sopen11g005580 has a 4-bp deletion (CACT, bp 885 to 888) that causes a frameshift mutation resulting in a premature stop codon (CTC ACT GAG TTA TCA CTA GGA→CTG AGT TAT CAC TAG (“amber”) GA). Moreover, Sopen11g005590 has a nucleotide substitution (G to A) at bp 1098, which causes a nonsense mutation (TGG→TGA (“opal”)). Therefore, these two genes are nonfunctional.

Despite the limited HexVic accumulation in *S. pennellii*, IL11-1, and IL11-2, based on the genomic synteny and structural similarity to UGT91R1 on chromosome 11 (Fig. 2a), Sopen11g005570, named UGT91R4, was identified as a locus allelic to UGT91R1 (Solyc11g010780), with 92% amino acid identity (Supplementary Fig. 6). Therefore, we evaluated the transcript accumulation of *UGT91R1* in M82 plants and that of *UGT91R4* in IL11-1, IL11-2, and *S. pennellii* plants. The transcript level of *UGT91R4* in *S. pennellii* was significantly lower than that of *UGT91R1* in M82 plants (*p* < 0.05, one-way ANOVA with Tukey’s multiple comparison test, Supplementary Fig. 5b). However, no significant difference in the transcript level of *UGT91R1* and *UGT91R4* between M82 and IL11-1 plants was observed, and the transcript level of *UGT91R4* in IL11-2 was slightly higher than that of *UGT91R1* in M82 plants (Supplementary Fig. 5b). These results suggest that the low transcript level of *UGT91R4* is partially responsible for the defective HexVic accumulation in *S. pennelli*, but not in IL11-1 or IL11-2.

Owing to the heterologous genomic structure of IL11-1 and IL11-2, UGT91R4 introgressed from *S. pennelli* is likely under transcriptional regulation in *S. lycopersicum* cv. M82. To further investigate defective HexVic accumulation in *S. pennellii* and the ILs, we performed biochemical characterization of UGT91R4 using UDP-arabinose as a sugar donor and HexGlc as a sugar acceptor. UGT91R4 produced HexVic in vitro, and the kinetic parameters of UGT91R4 were as follows: *K*_m_ value: 23 ± 2 μM, *k*_cat_ value: 0.26 ± 0.01 s^−1^, and *k*_cat_/*K*_m_ value: 11.2 s^−1^mM^−1^. The *k*_cat_ value of UGT91R4 was reduced to one-seventh and the *k*_cat_/*K*_m_ value was reduced to one-fifth compared with those of UGT91R1 (Table 2). Compared to the homology models of UGT91R1 and UGT91R4, we found conserved residues composed of the substrate pocket (Phe141, Gln388, Asp387) and catalytic dyad (His20, Asp118), but a polymorphic site at the 145^th^ position, Phe145 in UGT91R4 and Val145 in UGT91R4, respectively (Supplementary Fig. 7a–c). The lower specificity constant (*k*_cat_/*K*_m_) of UGT91R4 to UDP-arabinose than that of UGT91R1 suggested the less stabilizing contribution of the OH-π interaction between the aromatic ring of Phe141 and the 4-OH group of the arabinose moiety of UDP-arabinose; this could be attributed to the absence of Phe145 in UGT91R4 (Supplementary Fig. 7c). In line with the fact that the HexGlc content was comparable among M82, *S. pennellii*, IL11-1, and IL11-2 (Supplementary Fig. 8a), these results support the hypothesis that the lower catalytic activity of UGT91R4 in HexVic biosynthesis is responsible for altered HexVic accumulation in *S. pennellii*, IL11-1, and IL11-2 plants.

### Evaluation of HexGlc accumulation and function

The impairment in HexVic accumulation in *S. pennellii* suggested that its precursor monoglucoside, HexGlc, might be accumulated after (*Z*)-3-hexenol exposure. However, HexGlc accumulated at comparable levels in *S. lycopersicum*, *S. pennellii*, and ILs upon (*Z*)-3-hexenol exposure (Supplementary Fig. 8a, *p* > 0.3 for all pairwise comparisons, Dunnett’s multiple comparison test). As IL11-1 was susceptible to CCWs, HexGlc accumulation hardly accounts for the differences in CCW performance between *S. lycopersicum* and ILs. When CCWs were fed an artificial diet with or without HexGlc at an amount equivalent to that in (*Z*)-3-hexenol-exposed tomato leaves (0.25 μg·g^–1^ FW, Supplementary Fig. 8a) for 7 days, their weights were not significantly different (Supplementary Fig. 8b) (*p* = 0.2188, Wilcoxon’s signed-rank test). This result indicated that HexGlc has no defense activity against CCWs, highlighting the fact that UGT-mediated diglycosylation of (*Z*)-3-hexenol was necessary to exert a defense function *in planta*.

## Discussion

We evaluated the ability to biosynthesize HexVic, a defense compound against CCWs, in 17 tomato species and found that these species accumulate HexVic to varying levels upon exposure to (*Z*)-3-hexenol. Interestingly, domesticated tomato (*S. lycopersicum*) showed a higher ability to convert airborne (*Z*)-3-hexenol to HexVic than wild species such as *S. pennellii* (Table 1), suggesting that domesticated tomatoes have a more robust defense against CCWs than some wild species from the perspective of volatile-mediated defense. This was unexpected because, in general, domesticated microbes grown in artificial, less-competitive environments easily lose cooperative traits of social behaviors^25,26^ and domesticated plants invest less energy in defense against herbivory than wild plants^27,28^. For example, domesticated tomato plants tend to show a lower defense against *Manduca sexta*, a specialist herbivore of *Solanaceae* plants, than wild relatives^29^. The volatile glycosylation-mediated defense may be enhanced because of the domestication of tomato cultivars based on wild tomato species in agricultural environments.

The fact that CCWs performed better in terms of body weight on IL11-1 plants than on M82 plants can be partially explained by the lower amount of HexVic in IL11-1 plants, as HexVic has been shown to act as a defense compound in in vitro experiments^7^. It is important to note that HexGlc accumulated to similar levels in *S. pennellii*, ILs, and *S. lycopersicum* after (*Z*)-3-hexenol exposure (Supplementary Fig. 8a). Thus, *S. pennellii* can convert (*Z*)-3-hexenol to HexGlc, which would be mediated by other UGTs, represented by the UGT85 family^10,11,16,30,31^. Considering the difference in defense levels against herbivores between HexVic (defensive) and HexGlc (inert), the chemical structure of glycosides, for example, sugar group, linkage-type, or linkage position, seems to be crucial for the defensive activity against herbivores. Given that green leaf volatiles suppress the growth of both gram-positive and gram-negative bacteria^32^, the formation of (*Z*)-3-hexenol by hydrolysis of HexVic by insect-derived β-glycosidase in the mid-gut^33,34^ or by plant-derived diglycoside-specific glycosidase^35^ might attenuate the growth of gut microbes, thereby repressing the growth of CCWs. The roles of HexVic *in planta* and *in insecta* should be further studied using genetically modified tomato plants lacking UGT91R1.

We showed that HexVic biosynthesis from exogenous (*Z*)-3-hexenol requires a functional *UGT91* gene cluster on chromosome 11 (Fig. 2a). UGT91R1 can produce HexVic by transferring arabinose to the glucose moiety of HexGlc (Fig. 3a, b), supporting this notion. The structural similarity of other *UGT* genes in the cluster suggested that some of them also participate in the diglycosylation of airborne volatiles by exerting similar biochemical properties. The result also supports that the *UGT91R1*-KO tomatoes produce small amounts of HexVic upon exposure to (*Z*)-3-hexenol (Fig. 4a). Notably, UGT91R1 showed sugar donor preference for UDP-pentose, particularly UDP-arabinose (Table 2), but it did not catalyze glycosylation with UDP-hexoses such as UDP-glucose and UDP-galactose in vitro. UGT91R1 was likely to distinguish UDP-pentoses from UDP-hexose because it had a bulky Ile140 residue proximal to the sugar donor (Supplementary Fig. 7a–c), which was conserved among previously characterized pentosyltransferases^36,37^ and contributed to pentose preference (Table 3)^11,38^. Moreover, preferential production of HexVic by UGT91R1-catalyzed arabinosylation is determined using the Michaelis–Menten constant (*K*_m_) rather than turnover (*k*_cat_) for UDP-arabinose (Table 2), explaining the dominant presence of HexVic in tomatoes. Although we did not exclude the possibilities that metabolic fluxes (e.g., the intracellular UDP-sugar availability, subcellular localization of the enzymes and their substrates, and the efficiency of (*Z*)-3-hexenol uptake from the atmosphere), the substrate specificity of UGT91R4 also affects HexVic biosynthesis in IL11-1, IL11-2, and *S. pennellii*. Our study provides valuable insights into the underlying mechanisms of sugar donor specificity and catalytic efficiency of pentosyltransferases that have independently evolved in diverged land plants^11^.

**Table 3:** Comparison of substrate specificity of GGTs

		Substrate specificity			
UGT	Species	Sugar donor	Sugar acceptor	Sequence alignment	Reference
UGT91R1	Tomato (*S. lycopersicum*)	**UDP-arabinose/UDP-xylose**	Volatile glucoside	IPSGYFS **I** FIAA 140	**This work**
CsGT2 (UGT94P1)	Tea (*C. sinensis*)	**UDP-xylose**	Volatile glucoside	IPAVQLM **I** TGAT 141	11
AcF3GGT1	Kiwifruit (*A. chinensis*)	**UDP-xylose**	Flavonoid galactoside	IKSVNYC **I** ISPA 136	36
UGT79B1	*A. thaliana*	**UDP-xylose**	Flavonoid glucoside	AKTVCFN **I** VSAA 142	37
UGT94D1	Sesame (*S. indicum*)	UDP-glucose	Lignan glucoside	IPAMVFL S TGAA 140	53
UGT94F1	*V. persica*	UDP-glucose	Flavonoid glucoside	SPSVWFM A SGAT 144	19
NSGT1	Tomato (*S. lycopersicum*)	UDP-glucose	Volatile diglycoside	IHAIMFY V SSTS 145	15
GAME18	Tomato (*S. lycopersicum*)	UDP-glucose	Steroidal glycoalkaloid	IPSLRFY T VNAA 137	41

Bold-type letters in the sequence alignment indicate the positions of Ile-140 of UGT91R1 and the corresponding amino acid residues with UDP-pentoses such as UDP-arabinose or UDP-xylose as sugar donors.

Comparison of *UGT91R1* and *UGT91R4* sequences of *S. lycopersicum* and *S. pennellii* showed that the coding sequences are highly conserved (93%), whereas the intergenic sequences are not^39,40^. We did not exclude the possibility of other unknown mechanisms in the metabolic alteration in HexVic biosynthesis or catabolism, as over 290 genes are present in the introgressed genomic regions from *S. pennellii* (Supplementary Table 1). However, the possibility is unlikely as *UGT91R1*-KO and *UGT91R1*-OX tomatoes demonstrated reduced and increased HexVic accumulation, respectively (Fig. 4a, b). Based on these findings, we suggest that UGT91R1-mediated arabinosylation is the molecular basis of HexVic biosynthesis. The transcript of *UGT91R1* accumulates in the roots and aerial parts of *S. lycopersicum* based on data available on the Tomato expression database (http://bar.utoronto.ca/eplant_tomato/). Our quantitative analysis of the HexVic content in the roots of *S. lycopersicum* exposed to (*Z*)-3-hexenol demonstrated that HexVic accumulated in the roots and leaves (Supplementary Fig. 9), suggesting that similar trans-acting volatile glycosylation, mediated by UGT91R1, occurs in the rhizosphere and aboveground parts.

Besides the *UGT91* cluster described in this study, it should be noted that there are other tomato GGTs for different specialized glycosides, namely NON-SMOKY GLYCOSYLTRANSFERASE 1 (NSGT1) and GLYCOALKALOID METABOLISM 18 (GAME18). NSGT1 and GAME18 are glucosyltransferases for phenylpropene glycoside and steroidal glycoalkaloid, respectively, and do not share the Ile residue conserved among pentosyltransferases (Table 3). They are located in different gene clusters on chromosomes 9 and 7, respectively^15,41^. The gene multiplication of GGTs in the tomato genome likely enabled the production of diverse glycosides through functional differentiation. GGT multiplication is found in not only tomatoes but also various seed plants^19,42^. UGT-mediated glycosylation is hypothesized to have been originally developed for the storage and sequestration of endogenous reactive, lipophilic, and toxic compounds to avoid intoxication^43^ and was then exploited for the incorporation of exogenous volatile alcohols for defense^44^. The perception of various volatile alcohols by UGTs may contribute to other processes involved in volatile-mediated ecological interactions with rhizobacteria^45^, herbivores^46^, or other environmental factors^47^, which is an intriguing question that remains to be addressed.

## Methods

### Plants and growth conditions

As a domesticated species, *S. lycopersicum* cv. M82 (for comparison with wild species or ILs) was used. The seeds of wild species, *S. lycopersicum var*. cerasiforme (TOMJPF00009), *S. peruvianum* (TOMJPF00010), *S. pimpinellifolium* (TOMJPF00011), and *S. pennellii* (TOMJPF00008), were purchased from the National BioResource Project (NBRP) at the University of Tsukuba, Japan. The seeds of wild species, *S. pennellii* (LA0716, LA1376, LA1926, LA0751, LA1656, LA1946, LA1272, LA1674, LA2580, LA1277, LA1724, LA2657, LA1356, LA1732, LA2963, LA1367, and LA1733), *S. habrochaites* (LA1777), *S. lycopersicoides* (LA2951), *S. chilense* (LA1930), *S. galapagense* (LA0317), *S. cheesmaniae* (LA1041), *S. arcanum* (LA1360), *S. chmielewskii* (LA1316), *S. neorickii* (LA1319), *S. corneliomulleri* (LA1973), *S. sitiens* (LA1974), *S. huaylasense* (LA1982), *S. ochranthum* (LA2682), and *S. juglandifolium* (LA3322) were provided by the Tomato Genetic Resource Center (TGRC) at the University of California Davis, USA. ILs between *S. lycopersicum* cv. M82 and *S. pennellii* (LA0716) were purchased from the NBRP (TOMJPF00013s), except for IL1-1 and IL6-2-2, which were provided by the TGRC. Tomato plants were grown in plastic pots (8 cm in diameter) filled with soil (Ikubyou-baido, Takii Seed, Kyoto, Japan) in a glass house under a controlled temperature of 25 ± 2 °C and a 16 h photoperiod with natural and supplemented light. Four-to-five-week-old plants were used in all experiments, except for tomato fruits. We considered fully ripe red fruit as an indicator of the mature stage and green fruit as an indicator of the immature stage; both of them and leaves were used for RT-PCR assays and analysis of tissue-specific HexVic accumulation. The number of plants used in each experiment and the number of independent experiments are indicated in the figure legends.

### Generation of *UGT91R1*-KO and *UGT91R1*-OX tomatoes

The genome editing vector was constructed and transformed into *S. lycopersicum* cv. M82 seedlings. Briefly, potential target sequences were predicted using the CCTOP program^48^ with a CRISPRater editing score^49^. The oligo DNAs of two target sites were synthesized (Supplementary Table 4) and cloned into BbsI-digested pMR217 and pMR218 entry vectors^50^. The sgRNA expression cassette of entry vectors was transferred into the pDeCas9_Km vector using the Gateway system^51^. The constructed vector was transformed into *Rhizobium radiobacter* (agrobacterium) strain GV2260. *Agrobacterium* was used to infect tomato cotyledons as described by Sun et al. 2006^52^. Diploid plants among the regenerated shoots were selected using a flow cytometer (Quantum P, CYTOTECHS, Ibaraki, Japan), and their targeted genome was sequenced with *UGT91R1*-specific primers (Supplementary Table 3). Both edited and unedited plants were further grown under fluorescent light (approximately 150 μmol·m^–2^·s^–1^) until true leaves emerged. For the generation of *UGT91R1*-OX in IL11-1 and IL11-2, UGT91R1 cDNA was amplified using PCR with PrimeSTAR HS DNA Polymerase using the TO-2282-2 and TO-2283-2 primers and subcloned into the SalI-digested pBYR2HS-CFlagHis with an In-Fusion HD Cloning Kit (Takara Bio). The resulting construct, UGT91R1-pBYR2HS-CFlagHis, was transformed into *Agrobacterium tumefaciens* GV3101. The suspension of *A. tumefaciens* GV3101 harboring UGT91R1-pBYR2HS-CFlagHis was infiltrated into 4-week-old leaves of IL11-1 and IL11-2 plants. The infiltrated plants were grown under the same conditions as *UGT91R1*-KO tomatoes for 4 days.

### Treatment with volatile (*Z*)-3-hexenol

A 100-mM stock solution of (*Z*)-3-hexenol (Wako Pure Chemicals, Osaka, Japan) was prepared in dichloromethane. To measure the content of (*Z*)-3-hexenyl glycosides in tomatoes used in this study excluding mature M82 plants grown for 3 months, a single tomato plant was enclosed in a glass jar along with filter paper (0.25 cm^2^) containing 1 μmol (*Z*)-3-hexenol per liter of the container, corresponding to 24.4 ppmV (*Z*)-3-hexenol in the initial step of the exposure period. The exposed plants were left under the growth conditions from 8 am to 2 pm for sampling. A filter paper with the same volume of dichloromethane was used as a control treatment. Mature M82 plants grown for 3 months were exposed to (*Z*)-3-hexenol with leaves and fruits covered with polyvinyl fluoride bags instead of glass jars in the same conditions as described above.

### Quantification of HexVic in the tomatoes

Harvested leaves, stems, and fruits were flash-frozen in liquid nitrogen and stored at –80 °C until extraction. To quantify endogenous glycosides in wild, domesticated, and IL plants exposed to (*Z*)-3-hexenol, (*Z*)-3-hexenyl glycosides were extracted using ice-cold methanol from fine crashed target tissues (approximately 100 mg)^7^. To quantify HexVic content in *UGT91R1-*KO and *UGT91R1-*OX lines, approximately 500 mg of these tissues was finely crushed in a tissue mill (Taitec, Saitama, Japan), suspended in ice-cooled methanol containing an internal standard (2 µmol of 4-isopropyl benzyl β-d-glucopyranoside), and filtered. The filtrate was concentrated *in vacuo*, dissolved in distilled water, and purified with a Cleaner ODS C_18_ SPE column (Bonna-Agela Technologies, Tianjin, China). The glycosidic fractions were concentrated *in vacuo* and dissolved in distilled water before LC-MS analysis. The LC-MS analysis was performed on a LCMS-2020 system (Shimadzu, Kyoto, Japan), equipped with a Capcell Pak UG120 C_18_ reversed-phase column (2.0 mm i.d. × 150 mm, 5 μm; Shiseido, Tokyo, Japan) using gradient elution with aqueous formic acid (0.1%, v/v) as solvent A and acetonitrile as solvent B at a flow rate of 0.2 mL·min^–1^ at 40 °C. The gradient condition started with isocratic conditions of 9% of solvent B for 30 min, then increased up to 24% of solvent B for 5 min, and maintained 24% of solvent B for 33 min. HexVic content was calculated based on a calibration curve constructed using chemically synthesized HexVic in this work (Supplementary Data 3).

### Gene expression analysis

Harvested leaves, stems, and fruits were flash-frozen and pulverized in liquid nitrogen. The total RNA from the leaves and stems was extracted using an RNeasy Plant Mini Kit and RNase-Free DNase Set (Qiagen, Germantown, MD, USA). RNA from fruits was extracted with Fruit-mate for RNA-purification (TaKaRa Bio, Shiga, Japan). The RNA was reverse-transcribed to cDNA using the PrimeScript RT Reagent Kit (TaKaRa Bio). Gene expression was evaluated using semiquantitative RT-PCR (Supplementary Fig. 3a) and qRT-PCR (Supplementary Fig. 5b) with specific primers (Supplementary Table 3). Target mRNA levels were quantified using the ΔΔCt method and normalized to the expression level of the reference gene *GAPDH*^53^. The results are presented as mean ± SE of three independent experiments (Supplementary Fig. 5b).

### Enzymatic assays of recombinant UGT91R1

Full-length UGT91R1 cDNA synthesized using GeneArt Technology (Life Technologies, Carlsbad, CA, USA) was subcloned into the pET15b expression vector (Merck Millipore, Darmstadt, Germany) and transformed into *E. coli* BL21 (DE3). Recombinant protein expression was induced by incubating 200 mL of culture with 0.1 mM IPTG at 22 °C for 24 h. Proteins were harvested using ultrasonic disruption and were purified using 1 mL of HisTrap HP (GE Healthcare, Chicago, IL, USA). The purified enzyme was incubated with 100 mM sugar acceptor (HexGlc) and 2 mM sugar donor (UDP-arabinose (chemically or enzymatically synthesized), UDP-xylose (Complex Carbohydrate Research Center, GA, USA), or UDP-galactose, UDP-glucose, UDP- glucuronic acid (Sigma Aldrich, Tokyo, Japan)) in 50 mM potassium phosphate buffer (pH 7.5) at 30 °C for 15 min. The reaction was stopped by adding 50 μL of ice-cold methanol and quickly cooled with liquid nitrogen. The enzymatic reaction mixtures were analyzed using HPLC-MS with a Shimadzu LCMS-2020 system, coupled to a Capcell Pak UG120 C_18_ reversed-phase column (2.0 mm i.d. × 150 mm, 5 μm) under the following conditions: 5% solvent B (0–1 min), followed by a linear gradient flow up to 20% in 34 min (solvent A: H_2_O containing 0.05% (v/v) formic acid, B: acetonitrile).

### Homology modeling

Three-dimensional models of UGT91R1 and UGT91R4 were established using Discovery Studio 4.0 (DS4.0) (BIOVIA; https://www.3dsbiovia.com/). The crystal structure of AtUGT72B1 (PDB code 2vce) was used as a template. Initial structures were constructed using the homology modeling protocols of the DS4.0 Modeler module. The model structures of UGT91R1 and UGT91R4 were inserted into UDP-2F-Glc bound to the crystal structure of At_UGT72B1. Furthermore, the sugar moiety of UDP-2F-Glc bound to UGT91R1 or UGT91R4 was replaced with xylose or arabinose to construct UDP-xylose- and UDP-arabinose-bound UGT91R1, and UDP-arabinose-bound UGT91R4, respectively. Structure optimization of the three initial complex models, UGT91R1-UDP-xylose, UGT91R1-UDP-arabinose, and UGT91R4-UDP-arabinose, was performed using molecular mechanics and dynamic simulation with the CHARMM force field of DS4.0.

### Phylogenetic analysis

Nucleotide and amino acid sequences of UGTs were aligned based on codon position using ClustalW and MUSCLE in MEGA6, respectively^54^. All positions containing gaps and missing data were eliminated from the subsequent analysis. Phylogenetic trees were constructed using the maximum-likelihood method with T92 + G and JTT + G + I + F for nucleotide and amino acid sequences, respectively, based on the Bayesian Information Criterion using model test analysis^55^. The reliability of the tree was evaluated using bootstrap analysis of 1,000 replicates. The sequences used in this analysis are listed in Supplementary Data 1 and 2.

### Insect bioassay

The eggs of common cutworm (*Spodoptera litura*) were purchased from Sumika Technoservice (Hyogo, Japan) and were hatched in the laboratory. The hatched neonates were reared on an artificial diet (Insecta LFS, Nosan Co., Kanagawa, Japan) until they were used for experiments in a biotron under a controlled temperature of 25 ± 2 °C and a 16 h photoperiod of fluorescent light. To assay insect performance on plants, *S. lycopersicum* or the HexVic-deficient IL11-1 plants were challenged with the third instars for 7 days. To assay insect performance on the artificial diet, newly hatched neonates were fed the diet mixed with HexGlc at 0.25 μg·g^–1^, which is close to the average amount of HexVic detected in (*Z*)-3-hexenol-exposed tomato plants. The insects were weighed on a precision balance 7 days after the experiments.

### Statistics

All statistical analyses were conducted using R 3.3.0 (http://www.r-project.org/) and GraphPad Prism 9 (GraphPad Software Inc, California, USA). Data were analyzed using the one-way ANOVA and Tukey’s tests for multiple comparisons, Dunnett’s multiple comparison test for repeated pairwise comparisons, and unpaired two-tailed *t*-test and Welch’s *t*-test for pairwise comparisons. HexVic content was compared between *S. lycopersicum* and various accessions of *S. pennellii* to find outlier genotypes using the Smirnov–Grubbs test. Weight gain of insects on plants and artificial diet was analyzed using the Tukey’s multiple comparison and Wilcoxon signed-rank tests, respectively. Results were considered significant at α < 0.05.

### Reporting summary

Further information on research design is available in the Nature Portfolio Reporting Summary linked to this article.

## Supplementary information

Supplementary Information

Descriptions of Additional Supplementary Files

Supplementary Data 1

Supplementary Data 2

Supplementary Data 3

Reporting Summary

## Data Availability

All data are available in the main text or the supplementary materials. Data supporting the findings of this study are available within the article and the supplementary materials. Source data are provided with this paper.

## Source data

Source data 1
