# Supplementary material for: Identification of a tomato UDP-arabinosyltransferase for airborne volatile reception
Source: Nat Commun. 2023 Feb 8;14:677. doi: 10.1038/s41467-023-36381-8 (PMC9908901; doi:10.1038/s41467-023-36381-8)
Supplement: Supplementary file 1 — Supplementary Information [file 41467_2023_36381_MOESM1_ESM.pdf]

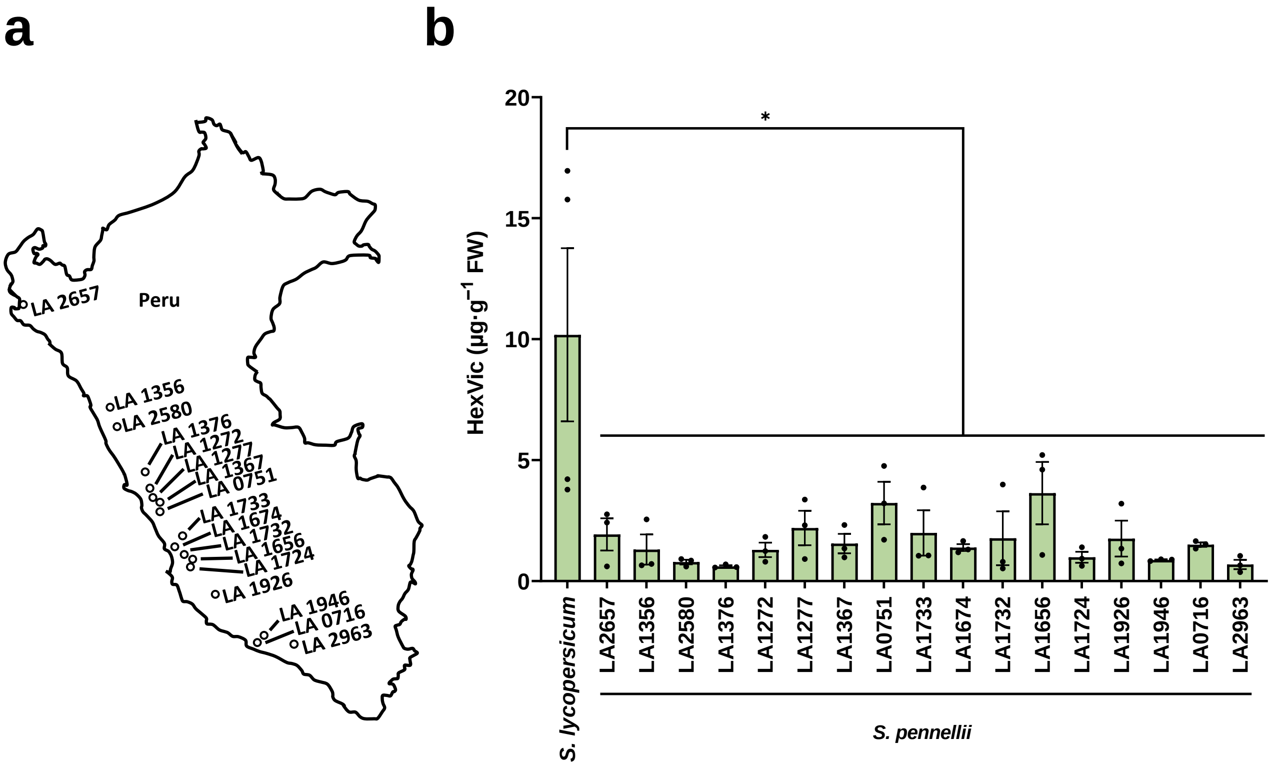

Supplementary Figure 1: HexVic accumulation in various *S. pennellii* accessions.

a Map showing geographical distribution of *S. pennellii* accession collection sites in Peru.

b HexVic contents in leaves of (*Z*)-3-hexenol-exposed plants of different *S. pennellii* accessions. Error bars indicate standard error of the mean from independent experiments (four plants for *S. lycopersicum* cv. M82 and three plants for each *S.pennellii* genotypes). Statistical significances were determined by Dunnett's multiple comparisons test. Statistically significant differences are indicated as *$p < 0.05$

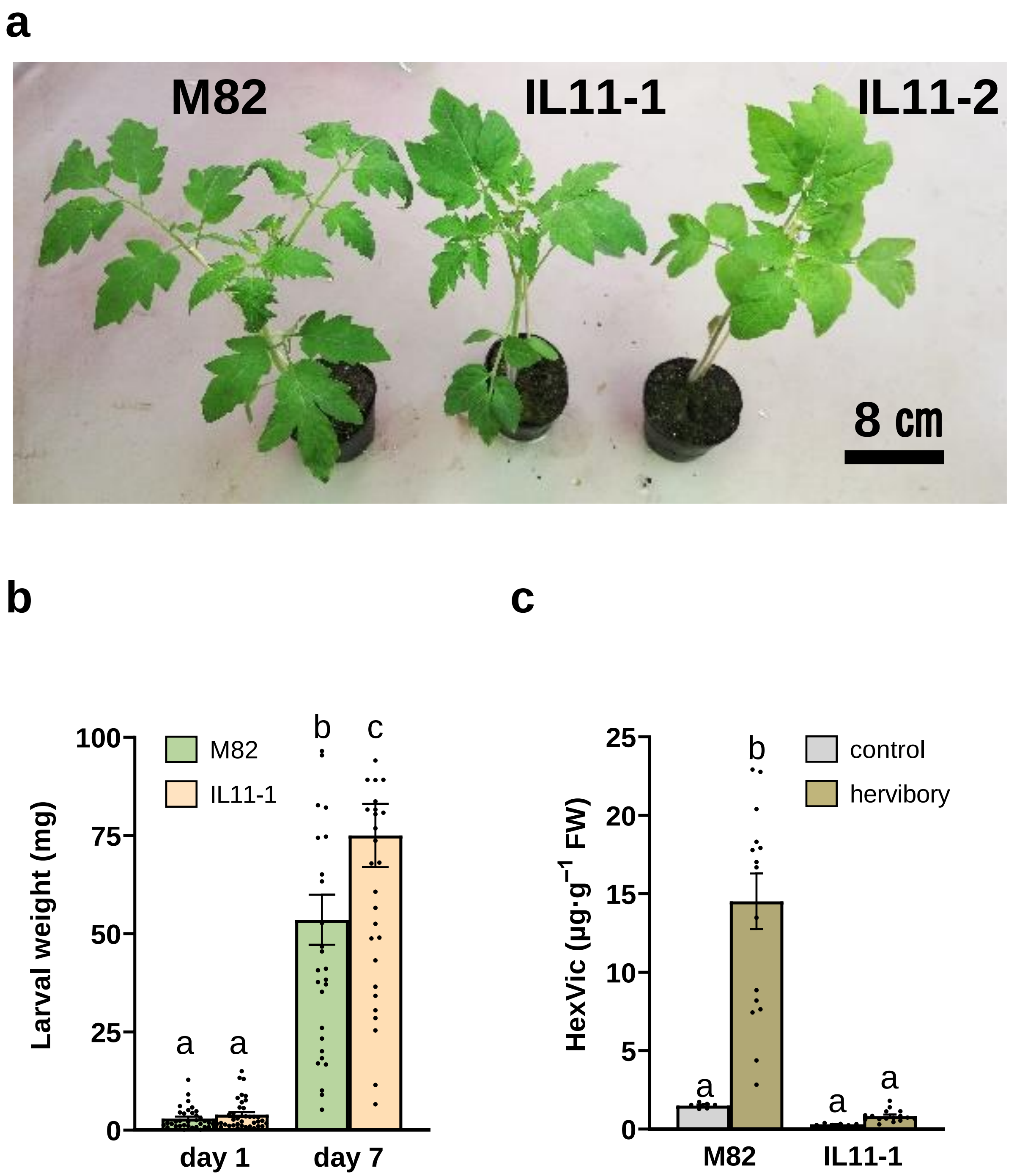

Supplementary Figure 2: Plant and insect phenotypes of HexVic-deficient tomato.
a Appearance of introgression lines (ILs). Five-week-old IL11-2 had a different shape and soft, light greenish leaves compared to wild-type M82 and IL11-1. **b** Weights of common cutworms before and after growth for seven days on M82 (green box) or IL11-1 (orange box). Each plant was challenged with a single third instar, and insects were weighed on days 1 and 7 after infestation. Data are means ± SE from 8 independent experiments (thirty-four plants for M82 and thirty-seven plants for IL11-1 on day 1, and thirty plants for M82 and thirty-two plants for IL11-1on day 7). Differences among lines and time points were analyzed using the Tukey's multiple comparison test, and significant differences are indicated with different characters ($p < 0.05$). **c** HexVic content in leaves of M82 and IL11-1 plants challenged or not with common cutworms for two days. Data are means ± SE from 3 independent experiments (six plants for M82 and IL11-1 as control, and sixteen plants for M82 and IL11-1for herbivory treatment). Differences among species and treatments were analyzed using the Tukey's multiple comparison test, and statistical differences are indicated with different characters ($p < 0.05$).

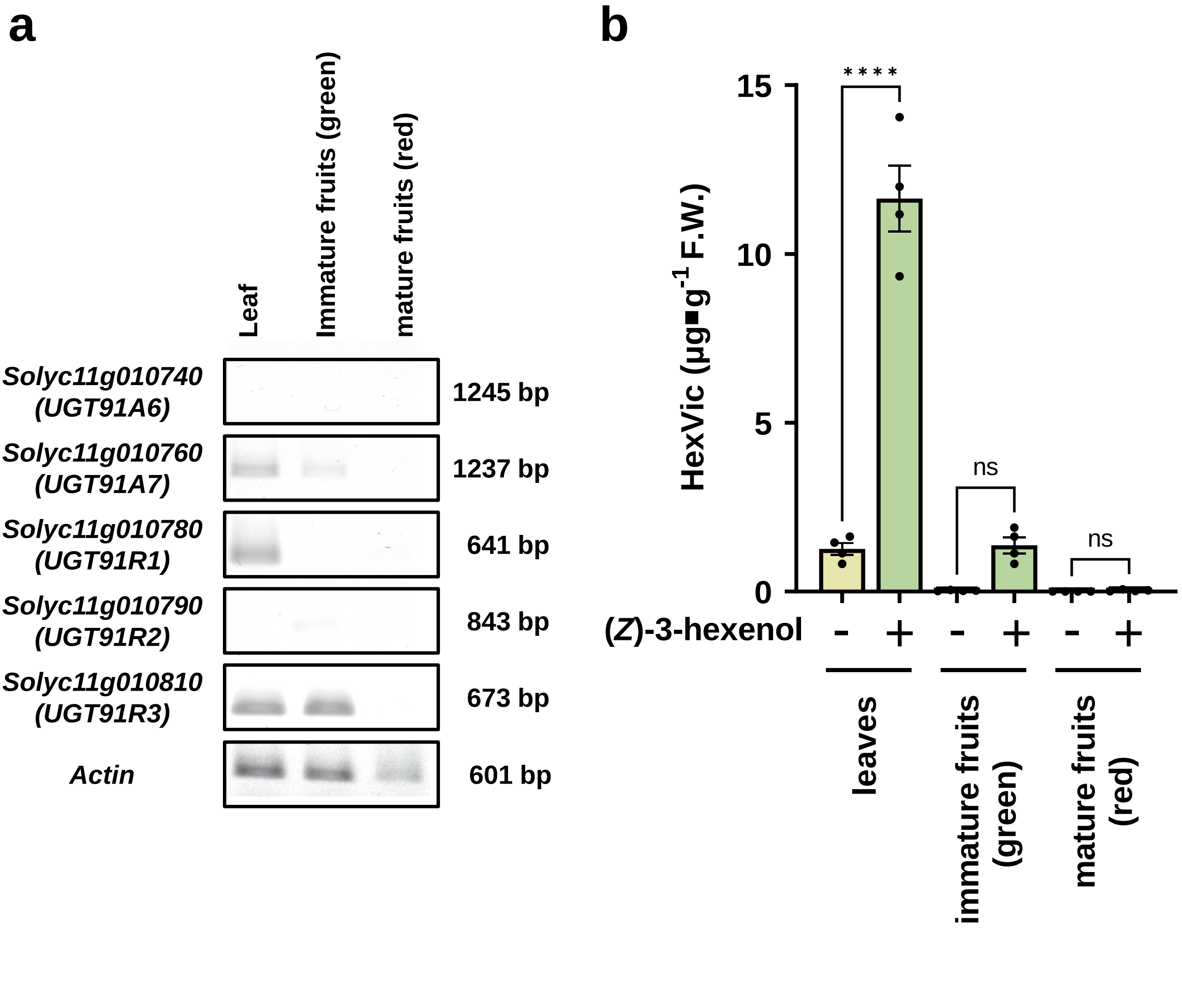

Supplementary Figure 3: Tissue-specific biosynthesis of HexVic in *S. lycopersicum* cv. M82.
a Gene expression of *UGT91* in different tissues of *S. lycopersicum* as analyzed using semiquantitative RT-PCR. **b** HexVic contents in leaves of *S. lycopersicum* exposed (+) or not (-) to (*Z*)-3-hexenol. Data are means ± SE from 4 independent experiments. *P* values were analyzed using One-way ANOVA test with the Tukey's multiple comparison. Statistical significant differences are indicated as ****$p < 0.0001$. ns indicates no statistical difference.

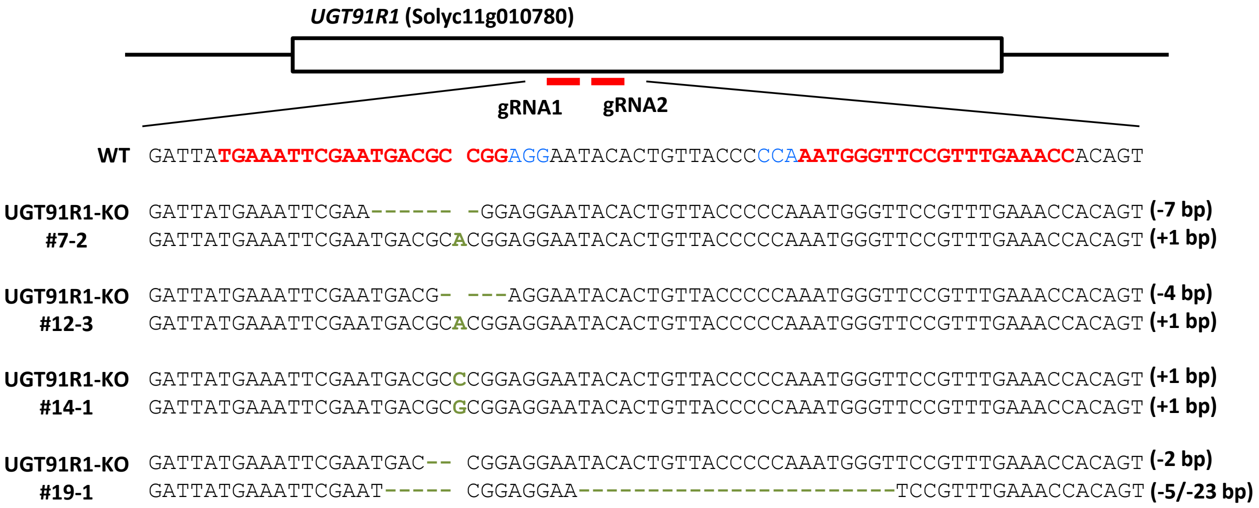

Supplementary Figure 4: Mutation alleles of tomato UGT91R1. Gene model of *UGT91R1* (*Solyc11g010780*) was depicted with a solid line (intergenic region) and open box (exon). Two gRNA regions for CRISPR-Cas9-based genome editing are indicated by red lines. Nucleotide sequence of target region contained gRNA and PAM sequences as red and blue characters, respectively. Nucleotide sequences of four knockout mutants harboring biallelic mutation are shown below sequence of WT. Insertion and deletion by genome editing are indicated as green characters.

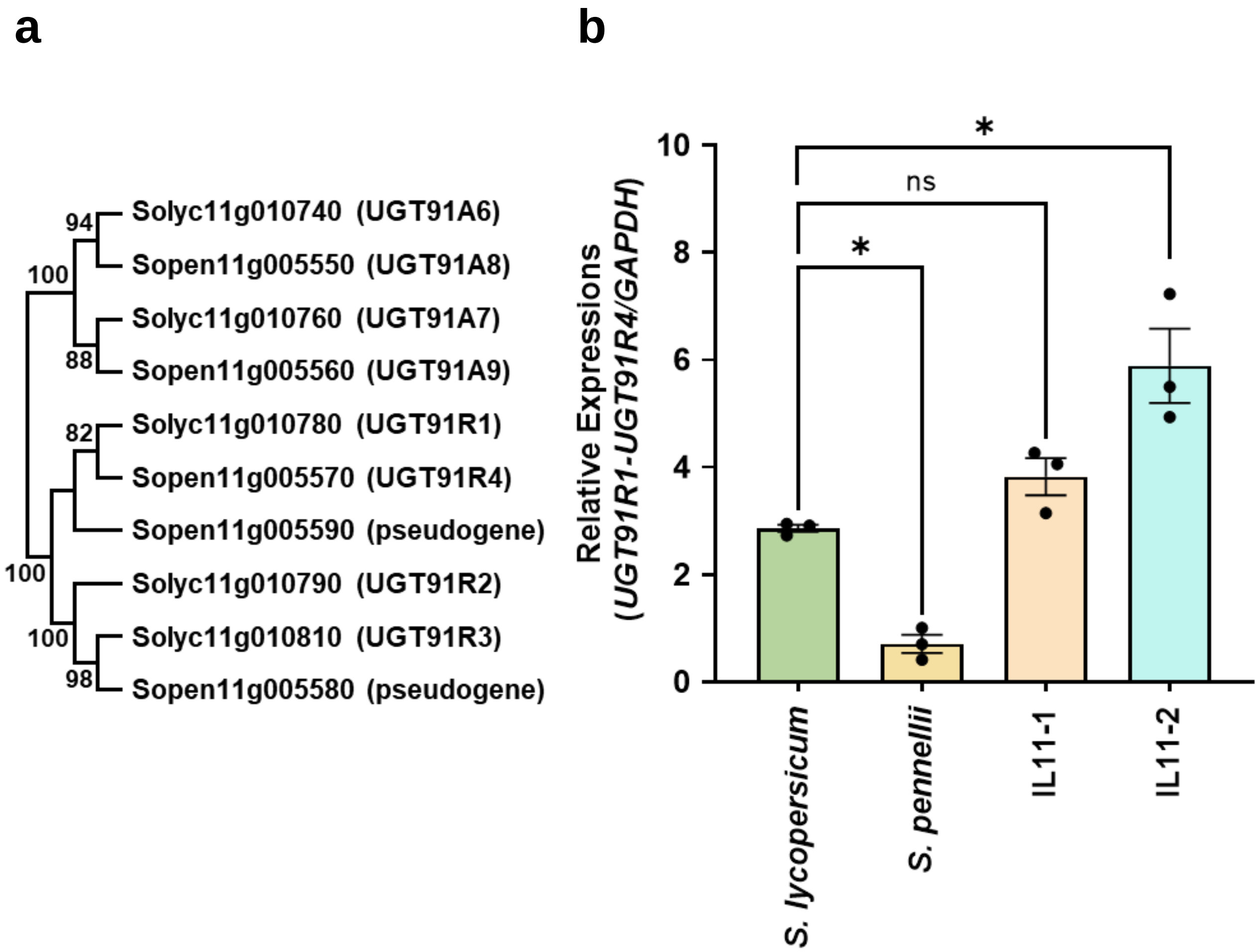

Supplementary Figure 5: Phylogenetic relationships of tomato *UGT91*s in tomato and *UGT91T1/UGT91R4* expression. a Nucleotide sequences of *UGT91* coding region were extracted from genome sequences of *S. lycopersicum* and *S. pennellii*. Phylogenetic tree was constructed using the maximum-likelihood method with 1000 bootstrap replications. **b** qRT-PCR analysis of *UGT91R1* and *UGT91R4* of leaves in *S. lycopersicum*, *S. pennellii* and two introgression lines (IL11-1 and IL11-2). Data are means ± SE from 3 independent experiments. *p* values were analyzed using One-way ANOVA test followed by the Tukey's multiple comparison. Statistical significant differences are indicated as $*p < 0.05$. ns indicates no statistical difference.

```
UGT91R1     ATGGCGGAAAACGGAAAAAAATTGCATATTGCAGTATTTCCATGGCTAGCTTTTGGTCAT    60
UGT91R1     M  A  E  N  G  K  K  L  H  I  A  V  F  P  W  L  A  F  G  H
UGT91R4     M  A  E  N  G  K  K  L  H  I  A  V  F  P  W  L  A  F  G  H
UGT91R4     ATGGCGGAAAACGGCAAAAAATTGCATATTGCAGTATTTCCATGGCTAGCTTTTGGTCAT    60
            ************** *********************************************

UGT91R1     ATGATTCCGTATTTAGAGCTATCAAAGCTTATAGCTCAAAAGGGTCATAAAATTTCATTC    120
UGT91R1     M  I  P  Y  L  E  L  S  K  L  I  A  Q  K  G  H  K  I  S  F
UGT91R4     M  I  P  Y  L  E  L  S  K  L  I  A  Q  K  G  H  I  I  S  F
UGT91R4     ATGATTCCGTATTTAGAGCTATCAAAGCTCATAGCTCAAAAGGGTCATATAATTTCATTC    120
            ***************************** ******************* **********

UGT91R1     ATTTCGACTCCTAGAAACATTGATCGTCTCCCAAAACTTCCACCAAATCTCACCCCTTTT    180
UGT91R1     I  S  T  P  R  N  I  D  R  L  P  K  L  P  P  N  L  T  P  F
UGT91R4     I  S  T  P  R  N  I  D  R  L  P  K  L  P  P  N  L  T  P  F
UGT91R4     ATTTCGACTCCTAGAAATATTGATCGTCTCCCAAAACTTCCACCAAATCTTACTCCCTTT    180
            ***************** ******************************** ** ** ***

UGT91R1     TTAAATTTTGTCAAACTTCCGATGCCCCACGTCGAAAAGTTGCCGGAAAATGCTGAAGCC    240
UGT91R1     L  N  F  V  K  L  P  M  P  H  V  E  K  L  P  E  N  A  E  A
UGT91R4     F  N  F  V  K  L  P  M  P  H  V  E  K  L  P  E  N  A  E  A
UGT91R4     TTCAATTTCGTCAAACTTCCGATGCCCCACGTGGAAAAGTTGCCGGAAAATGCAGAAGCC    240
            ** ***** *********************** ******************** ******

UGT91R1     ACCATTGATTTACCTTACGAGCAAGTCAAGTACCTCAAACTTGCTCAAGATGCACTACAA    300
UGT91R1     T  I  D  L  P  Y  E  Q  V  K  Y  L  K  L  A  Q  D  A  L  Q
UGT91R4     T  I  D  L  P  Y  E  Q  V  K  Y  L  K  L  A  H  D  A  L  Q
UGT91R4     ACCATTGATTTACCTTATGAGCAAGTCAAGTATCTCAAACTTGCTCATGATGCACTACAA    300
            ***************** ************** ************** ************

UGT91R1     GAATCGATGTCTAAGTTTATCGAAGATTCAGATATTGATTTTATACTATTCGATTTTACT    360
UGT91R1     E  S  M  S  K  F  I  E  D  S  D  I  D  F  I  L  F  D  F  T
UGT91R4     E  S  M  A  K  F  L  E  D  S  D  I  D  F  I  L  F  D  F  A
UGT91R4     GAATCAATGGCTAAGTTTCTCGAAGATTCAGATATTGATTTTATACTATTTGATTTTGCC    360
            ***** *** ******** ******************************* ****** * 

UGT91R1     TCTTATTGGGTTCCTTCAATTGCTTCAAAATTCAACATTCCGTCGGGATATTTCAGCATA    420
UGT91R1     S  Y  W  V  P  S  I  A  S  K  F  N  I  P  S  G  Y  F  S  I
UGT91R4     S  Y  W  I  P  S  I  A  S  K  F  N  I  P  T  G  Y  F  S  I
UGT91R4     TCTTATTGGATTCCTTCAATTGCTTCAAAATTCAACATTCCGACGGGTTACTTCAGCATA    420
            ********* ******************************** **** ** *********

UGT91R1     TTCATCGCTGCGTTTCTGGGTTTCACCGGACCTGTGCCGGGATTGAACAATGATTATGAA    480
UGT91R1     F  I  A  A  F  L  G  F  T  G  P  V  P  G  L  N  N  D  Y  E
UGT91R4     F  I  A  A  V  L  G  F  I  G  S  E  P  G  L  N  N  D  Y  Q
UGT91R4     TTCATAGCTGCGGTTCTGGGTTTCATCGGATCTGAGCCAGGATTGAACAATGATTATCAA    480
            ***** ****** ************ **** *** *** ****************** **

UGT91R1     ATTCGAATGACGCCGGAGGAATACACTGTTACCCCCAAATGGGTTCCGTTTGAAACCACA    540
UGT91R1     I  R  M  T  P  E  E  Y  T  V  T  P  K  W  V  P  F  E  T  T
UGT91R4     I  R  K  T  P  E  E  Y  T  V  S  P  N  W  V  P  F  E  T  T
UGT91R4     ATTCGGAAGACGCCAGAGGAATACACTGTTTCCCCAAATTGGGTGCCGTTTGAAACCACA    540
            ***** * ****** *************** **** ** ***** ***************
```

```
UGT91R1   GTTGCTTTCAAGCTTTTCGAAGTCTCGAGAATCTTCGAAGCTTCCATGAAAGGAGAGGAA   600
UGT91R1   V  A  F  K  L  F  E  V  S  R  I  F  E  A  S  M  K  G  E  E
UGT91R4   V  A  F  K  L  F  E  V  S  R  I  F  E  A  S  M  K  G  E  E
UGT91R4   GTTGCTTTCAAGCTTTTCGAAGTTTCGAGAATCTTCGAAGCTTCCATGAAGGGAGAAGAA   600
          *********************** ************************** ***** ***

UGT91R1   GAGAACATTGCTGATATTGTTCGTTACTATAGATCTGTTGAAAACTGTGATTTTTTGCTT   660
UGT91R1   E  N  I  A  D  I  V  R  Y  Y  R  S  V  E  N  C  D  F  L  L
UGT91R4   D  N  V  S  D  I  I  R  M  Y  K  G  L  R  Y  S  D  F  L  L
UGT91R4   GACAATGTTTCTGATATTATTCGTATGTATAAAGGTCTTAGATACTCTGATTTTTTGCTT   660
          ** **  ** ******** *****   **** *  * **  * *** *************

UGT91R1   GTGAGGAGCTGTTCAGAATTTGAACCAGAATGGTTGAAAGTTGTCGGAGATATTCACCGG   720
UGT91R1   V  R  S  C  S  E  F  E  P  E  W  L  K  V  V  G  D  I  H  R
UGT91R4   V  R  S  C  S  E  F  E  P  E  W  L  K  V  V  G  D  I  H  R
UGT91R4   GTGAGGAGCTGTTCGGAATTTGAACCCGAATGGTTGAAAGTTGTCGGAGATATCCACCGG   720
          ************** *********** ************************** ******
                                                 Primer F for qRT-PCR  --
UGT91R1   AAACCGGTTTTTCCGGTGGGTCAACTTCCGACTACACCGTATGAAGATGACAGCACGAAG   780
UGT91R1   K  P  V  F  P  V  G  Q  L  P  T  T  P  Y  E  D  D  S  T  K
UGT91R4   K  P  V  F  P  V  G  Q  L  P  T  T  P  Y  E  D  D  S  T  K
UGT91R4   AAGCCGGTTTTTCCGGTGGGTCAACTTCCGACTACGCCGTATGAAGATGACAGCACGAAG   780
          ** ******************************** ************************
          -------------------→
UGT91R1   ATCGATGCATGGAGAGAGATAAAGCTATGGCTTGATAAGCAAGAAAAGGGGAAAGTTATT   840
UGT91R1   I  D  A  W  R  E  I  K  L  W  L  D  K  Q  E  K  G  K  V  I
UGT91R4   I  D  A  W  R  E  I  K  L  W  L  D  K  Q  E  K  G  K  V  I
UGT91R4   ATCGATGCATGGAGAGAGATAAAACTATGGCTTGATAAGCAAGAAAAGGGGAAAGTTATT   840
          *********************** ************************************

UGT91R1   TACGTTGCATTTGGGAGCGAGGCAAAACCGAGTCAAAATGAACTTACTGAGTTATCTCTT   900
UGT91R1   Y  V  A  F  G  S  E  A  K  P  S  Q  N  E  L  T  E  L  S  L
UGT91R4   Y  V  A  F  G  S  E  A  K  P  S  Q  N  E  L  T  E  L  S  L
UGT91R4   TACGTTGCATTTGGGAGCGAGGCAAAACCGAGTCAAAATGAACTTACTGAGTTATCACTC   900
          ******************************************************** ** 

UGT91R1   GGGTTAGAGCTTTCTGGGTTGCCATTCTTTTGGGTTTTAAGAATTAAAAGAGGGGAGTCC   960
UGT91R1   G  L  E  L  S  G  L  P  F  F  W  V  L  R  I  K  R  G  E  S
UGT91R4   G  L  E  L  S  G  L  P  F  F  W  V  L  R  I  K  R  G  E  S
UGT91R4   GGACTAGAGCTTTCTGGGTTGCCATTCTTTTGGGTTTTAAGAATTAAAAGAGGGGAGTCC   960
          **  ********************************************************
          ←----------------------  Primer R for qRT-PCR
UGT91R1   GATGATGAATTGATTCAATTACCAGAAGGTTTCGAAGAACGAACAAAGGGAAGAGGAATA   1020
UGT91R1   D  D  E  L  I  Q  L  P  E  G  F  E  E  R  T  K  G  R  G  I
UGT91R4   D  D  E  L  I  Q  L  P  E  G  F  E  E  R  T  K  G  R  G  I
UGT91R4   GATGATGAATTGATTCAATTACCAGAAGGTTTCGAAGAACGAACAAAGGGACGAGGAATA   1020
          *************************************************** ********

UGT91R1   GTGTGCACGAGTTGGGCACCACAACTCAAGATACTGAGTCATGACTCAGTGGGTGGATTT   1080
UGT91R1   V  C  T  S  W  A  P  Q  L  K  I  L  S  H  D  S  V  G  G  F
UGT91R4   V  Y  T  S  W  A  P  Q  L  K  I  L  S  H  D  S  V  G  G  F
UGT91R4   GTGTACACAAGTTGGGCCCCACAACTCAAGATACTGAGTCATGATTCAGTAGGTGGGTTT   1080
          **** *** ******** ************************** ***** ***** ***
```

```
UGT91R1    TTGACTCATTCAGGATGGAGTTCAGTAGTCGAGGCAATACAATTTGAAAAGTCATTGGTT    1140
UGT91R1    L  T  H  S  G  W  S  S  V  V  E  A  I  Q  F  E  K  S  L  V
UGT91R4    L  T  H  S  G  W  S  S  V  V  E  A  I  Q  F  E  K  S  L  V
UGT91R4    TTGACTCATTCAGGATGGAGTTCAGTAGTCGAGGCAATACAATTTGAGAAGTCATTGGTT    1140
           *********************************************** ************

UGT91R1    CTCTTAACATTTTTGGCTGATCAAGGGATAAATGCTAGGCTTTTGGAGGAGAAGAAAATG    1200
UGT91R1    L  L  T  F  L  A  D  Q  G  I  N  A  R  L  L  E  E  K  K  M
UGT91R4    L  L  T  F  L  A  D  Q  G  I  N  A  R  L  L  E  E  K  K  M
UGT91R4    CTCTTAACATTTTTGGCTGATCAAGGGATAAATGCTAGGCTCTTGGAGGAGAAGAAGATG    1200
           ***************************************** ************** ***

UGT91R1    GCGTATTCGATACCGAGAAATGATCAAGACGGATCATTCACTCGTGACTCAGTGGCCGAG    1260
UGT91R1    A  Y  S  I  P  R  N  D  Q  D  G  S  F  T  R  D  S  V  A  E
UGT91R4    A  Y  S  I  P  R  N  D  Q  D  G  S  F  T  R  H  S  V  A  E
UGT91R4    GCATATTCGATACCGAGAAATGATCAAGACGGGTCGTTCACTCGTCACTCAGTGGCCGAG    1260
           ** ***************************** ** ********* **************

UGT91R1    TCACTGAATTTGGTACTCGTTAAAGAAGAGGGTTTTATTTATCGAGAAAAGATTAAAGAG    1320
UGT91R1    S  L  N  L  V  L  V  K  E  E  G  F  I  Y  R  E  K  I  K  E
UGT91R4    S  L  K  L  V  L  T  K  K  E  G  F  I  Y  R  E  K  I  K  E
UGT91R4    TCATTGAAACTGGTACTCACTAAAAAAGAGGGTTTTATTTATCGAGAAAAGATTAAAGAG    1320
           *** ****  ********  **** ***********************************

UGT91R1    ATGAAAGATCTTTTCTGTGACAAGAAAAGGCAAAATAATTATGTGGAGAATTTGTTAAGT    1380
UGT91R1    M  K  D  L  F  C  D  K  K  R  Q  N  N  Y  V  E  N  L  L  S
UGT91R4    M  K  N  L  F  C  D  K  E  R  Q  N  N  Y  V  E  N  L  L  S
UGT91R4    ATGAAAAATCTTTTCTGTGACAAGGAACGACAAAATAATTATGTGGAGAATTTGTTGAGT    1380
           ****** ***************** ** * ************************** ***

UGT91R1    TTTCTTCAAGACTATGAAAAGATTAAAGCATGA                               1413
UGT91R1    F  L  Q  D  Y  E  K  I  K  A  *
UGT91R4    F  L  Q  N  Y  E  K  I  K  A  *
UGT91R4    TTTCTTCAAAATTATGAAAAGATTAAAGCATGA                               1413
           ********* * *********************
```

Supplementary Figure 6: Comparison of putative amino acid sequence and ORF sequence of UGT91R1 and UGT91R4. Different bases in nucleotide sequence are indicated in red letters. Different bases in nucleotide sequence are indicated in red letters. Different residues in amino acid sequence are shown in red and yellow highlights. Blue arrows indicate annealing regions of specific primers for qRT-PCR.

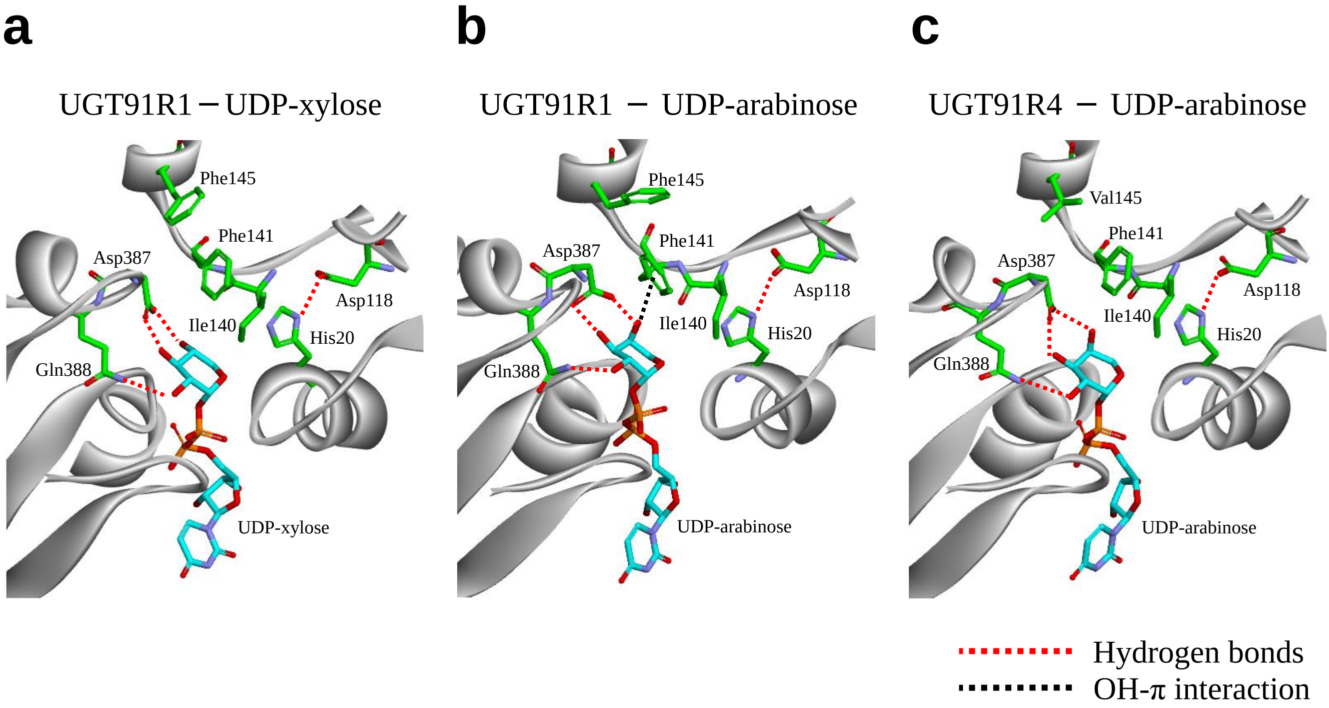

Supplementary Figure 7: Homology models of UGT91R1 and UGT91R4 with UDP-sugar (UDP-xylose/UDP-arabinose). a Homology model of UGT91R1 docking with UDP-xylose. **b** Homology model of UGT91R1 docking with UDP-arabinose. **c** Homology model of UGT91R4 docking with UDP-arabinose. Gene 3D models for UGT91R1 and UGT91R4 were constructed using the crystal structure of At_UGT72B1 (PDB code 2vce) as a template. For homology models, crucial amino acid residues in the active site and UDP sugars are drawn as sticks. Carbon atoms are colored green for UGT amino acid residues and cyan for UDP sugars. Oxygen atoms are red, nitrogen atoms are blue, and phosphorus atoms are orange. Red dotted lines indicate plausible hydrogen bonds, and a black dotted line indicates OH-π interaction. The sugar acceptor's structure is removed to simplify the models' visibility.

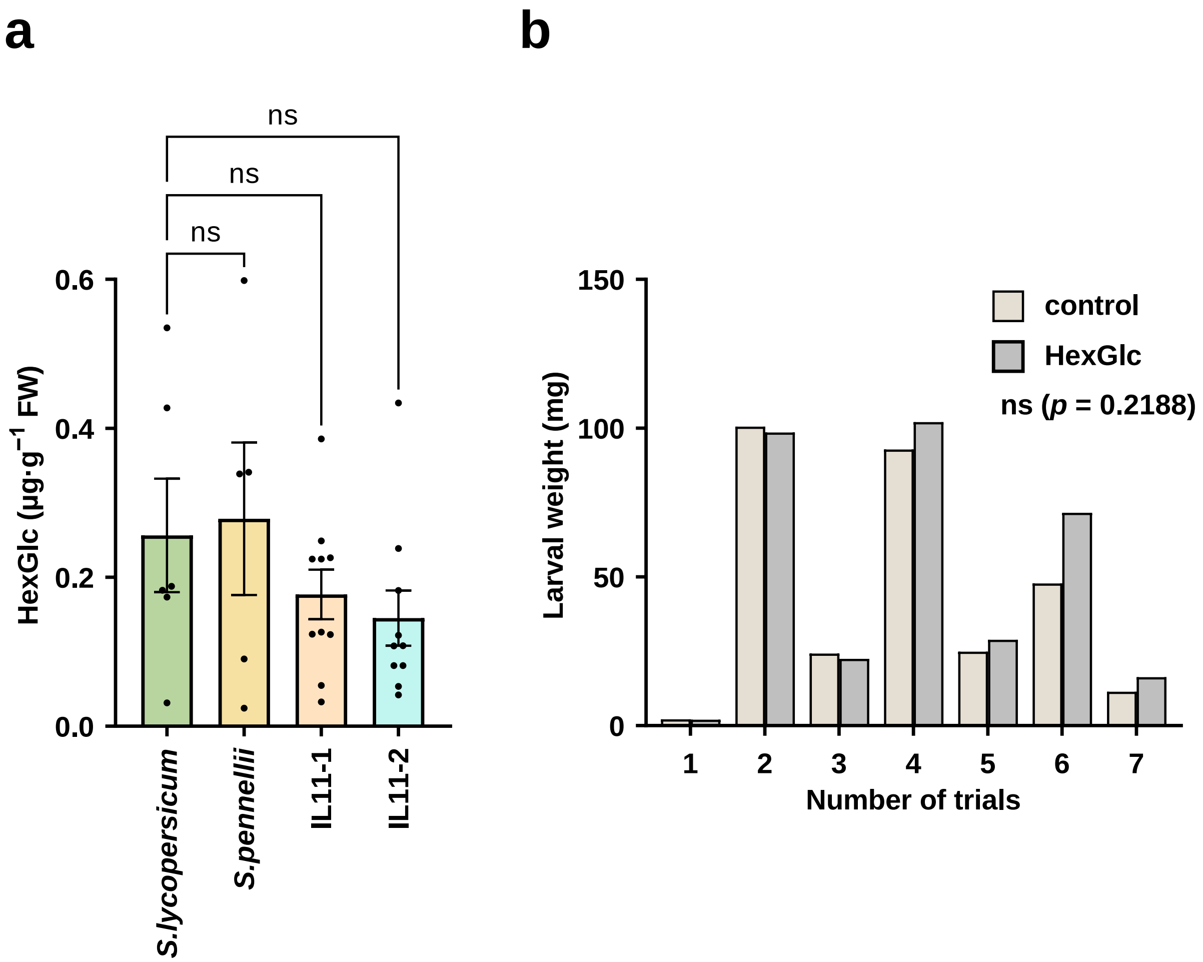

Supplementary Figure 8: Biosynthesis and function of (*Z*)-3-hexenyl β-D-glucopyranoside. a HexGlc content in leaves of *S. lycopersicum*, *S. pennellii*, and two introgression lines (IL11-1 and IL11-2). Plants were exposed to (*Z*)-3-hexenol for 6 h. Data are means ± SE from six plants for *S.lycopersicum*, five plants for *S. pennellii* and ten plants for two introgression lines (IL11-1 and IL11-2). Statistical significance tests were analyzed using one-way ANOVA test with Tukey's multiple comparison. ns indicates no statistical difference. **b** Common cutworms were weighed before and after a 7-day experimental period on a control or HexGlc-containing artificial diet. Data are from 7 independent experiments and were analyzed using the Wilcoxon signed-rank tests.

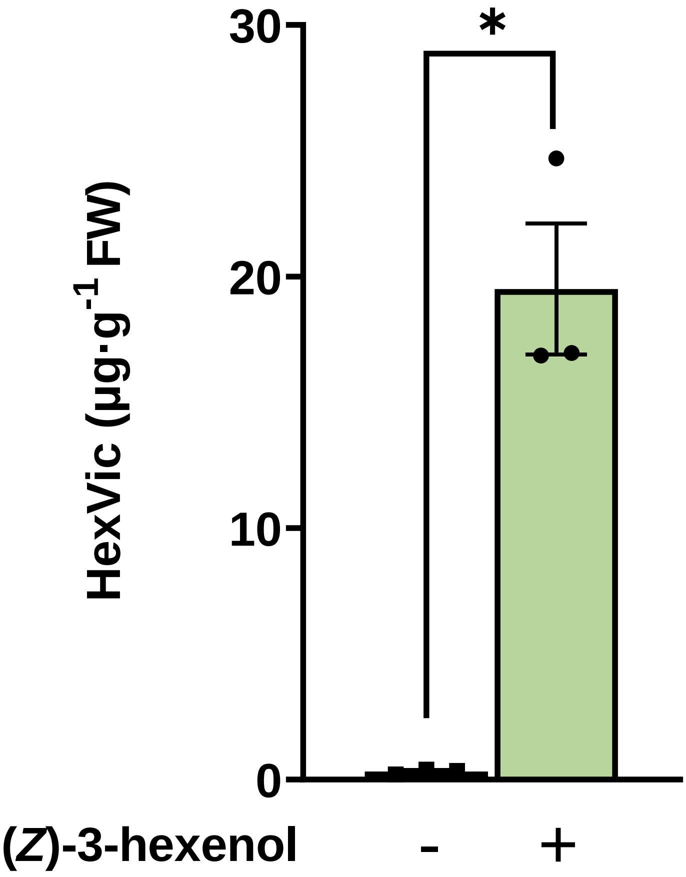

Supplementary Figure 9: HexVic contents in roots of *S. lycopersicum* cv. M82 exposed to (*Z*)-3-hexenol. A plus symbol indicates (*Z*)-3-hexenol exposure and a minus symbol indicates control. Data are presented as the means ± SE from three independent experiments. Statistical significance test was analyzed using two-tailed unpaired *t*-test. Statistical significant difference is indicated as $*p < 0.05$.

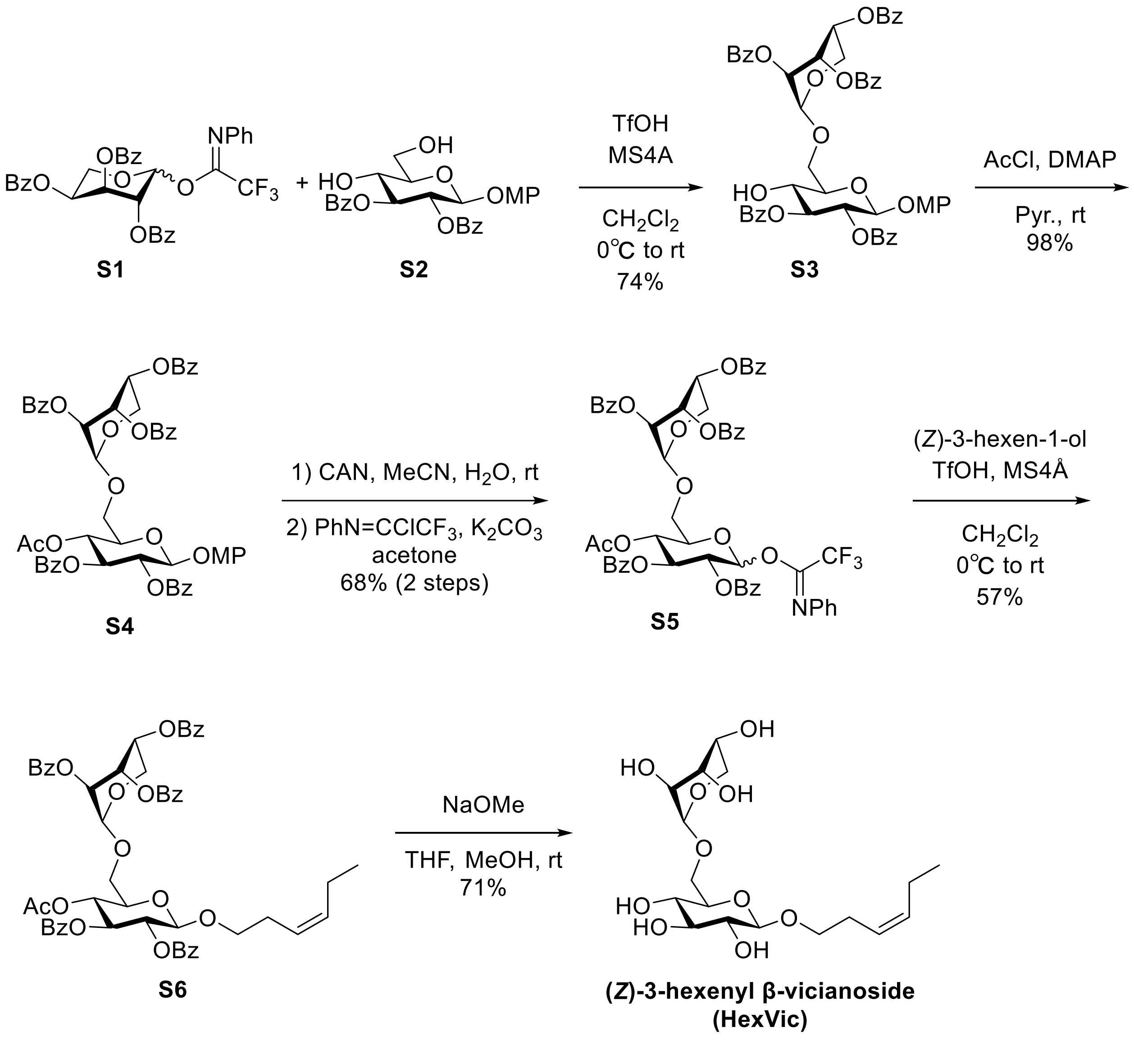

Supplementary Figure 10: Synthesis of (*Z*)-3-hexenyl β-vicianoside (HexVic). S1; 2,3,4-tri-*O*-Benzoyl-L-arabinopyranosyl (*N*-phenyl)trifluoroacetimidate, **S2**; *p*-methoxyphenyl 2,3-di-*O*-benzyl-β-D-glucopyranoside, **S3**; *p*-methoxyphenyl (2,3,4-tri-*O*-Benzoyl-α-L-arabinopyranosyl) -(1→6)-2,3-di-*O*-benzyl-β-D-glucopyranoside, **S4**; *p*-methoxyphenyl (2,3,4-tri-*O*-Benzoyl-α-L-arabinopyranosyl)-(1→6)-2,3-di-*O*-benzyl-4-*O*-acetyl-β-D-glucopyranoside, **S5**; (2,3,4-tri-*O*-Benzoyl-α-L-arabinopyranosyl)-(1→6)-2,3-di-*O*-benzyl-4-*O*-acetyl-D-glucopyranosyl 2,2,2-trifluoro-*N*-phenyl-acetimidate, S6; 3-*cis*-hexenyl (2,3,4-tri-*O*-Benzoyl-α-L-arabinopyranosyl)-(1→6)-2,3-di-*O*-benzyl-4-*O*-acetyl-β-D-glucopyranoside.

Supplementary Table 1. List of genes located at the loci where IL11-1 and IL11-2 overlap.

Red characters indicate UGT91 genes.

	Gene ID	description
30165	Solyc11g008070.1	Protein FAM126B IPR018619 Hyccin
30166	Solyc11g008080.1	Unknown Protein IPR012862 Protein of unknown function DUF1635
30167	Solyc11g008090.1	Shugoshin-1
30168	Solyc11g008100.1	Unknown Protein
30169	Solyc11g008110.1	Genomic DNA chromosome 5 P1 clone MJJ3
30170	Solyc11g008120.1	Genomic DNA chromosome 5 P1 clone MJJ3
30171	Solyc11g008130.1	Nuclear pore complex protein-related protein (Fragment)
30172	Solyc11g008140.1	Pectate lyase family protein IPR002022 Pectate lyase_Amb allergen
30173	Solyc11g008150.1	Transcription elongation factor spt5 IPR005100 Supt5 repeat
30174	Solyc11g008160.1	Ulp1 protease family C -terminal catalytic domain containing protein IPR003653 Peptidase C48, SUMO_Sentrin_Ubl1
30175	Solyc11g008180.1	Nodulin family protein IPR010658 Nodulin -like
30176	Solyc11g008190.1	Nodulin family protein IPR010658 Nodulin -like
30177	Solyc11g008200.1	Nodulin-like protein (Fragment) IPR010658 Nodulin -like
30178	Solyc11g008210.1	Glycine-rich RNA-binding protein-like IPR015465 RNA recognition motif, glycine rich protein
30179	Solyc11g008220.1	Unknown Protein
30180	Solyc11g008230.1	SKP1-like 1 IPR016897 E3 ubiquitin ligase, SCF complex, Skp subunit
30181	Solyc11g008240.1	SKP1-like protein IPR016897 E3 ubiquitin ligase, SCF complex, Skp subunit
30182	Solyc11g008250.1	Single-stranded nucleic acid binding R3H domain protein IPR003959
30183	Solyc11g008260.1	Cysteine proteinase cathepsin F IPR013128 Peptidase C1A, papain
30184	Solyc11g008270.1	Genomic DNA chromosome 3 P1 clone MSJ11
30185	Solyc11g008280.1	Serine carboxypeptidase IPR001563 Peptidase S10, serine carboxypeptidase
30186	Solyc11g008290.1	RNA binding protein IPR012677 Nucleotide -binding, alpha-beta plait
30187	Solyc11g008300.1	F-box family protein IPR001810 Cyclin -like F-box
30188	Solyc11g008310.1	Unknown Protein IPR019320 Protein of unknown function NEP
30189	Solyc11g008320.1	Lysine-specific histone demethylase 1 IPR002937 Amine oxidase
30190	Solyc11g008330.1	Zeta2-COP IPR011012 Longin -like
30191	Solyc11g008340.1	Exocyst complex component 4 IPR007191 Sec8 exocyst complex component specific domain
30192	Solyc11g008350.1	Kinesin-like protein IPR001752 Kinesin, motor region
30193	Solyc11g008360.1	Pseudouridine synthase family protein IPR 006145 Pseudouridine synthase, RsuA and RluB_C_D_E_F
30194	Solyc11g008370.1	Mitochondrial glycerol-3-phosphate dehydrogenase

30195	Solyc11g008380.1	Glycerol-3-phosphate dehydrogenase IPR000447 FAD -dependent glycerol -3-phosphate dehydrogenase
30196	Solyc11g008390.1	U-box domain-containing protein 14 IPR011989 Armadillo -like helical
30197	Solyc11g008400.1	SKP1-like protein IPR016897 E3 ubiquitin ligase, SCF complex, Skp subunit
30198	Solyc11g008410.1	Unknown Protein
30199	Solyc11g008420.1	SKP1-like protein IPR016897 E3 ubiquitin ligase, SCF complex, Skp subunit
30200	Solyc11g008430.1	Ras-related protein Rab-5C IPR015599 Rab5 -related
30201	Solyc11g008440.1	Amino acid transporter IPR013057 Amino acid transporter, transmembrane
30202	Solyc11g008450.1	Thioredoxin family protein IPR013766 Thioredoxin domain
30203	Solyc11g008460.1	U4_U6.U5 tri-snRNP-associated protein 1 IPR005011 SART -1 protein
30204	Solyc11g008470.1	U4_U6.U5 tri-snRNP-associated protein 1 IPR005011 SART -1 protein
30205	Solyc11g008480.1	Photosystem II oxygen evolving complex protein PsbP IPR016123 Mog1_PsbP, alpha_beta_alpha sandwich
30206	Solyc11g008490.1	Pumilio-like IPR011989 Armadillo -like helical
30207	Solyc11g008500.1	Actin IPR004000 Actin_actin -like
30208	Solyc11g008510.1	60S ribosomal protein L38 IPR002675 Ribosomal protein L38e
30209	Solyc11g008520.1	Ribonuclease 3-like protein 3 IPR000999 Ribonuclease III
30210	Solyc11g008530.1	Ribonuclease 3-like protein 3 IPR000999 Ribonuclease III
30211	Solyc11g008540.1	Ribonuclease 3-like protein 3 IPR000999 Ribonuclease III
30212	Solyc11g008550.1	Lecithin cholesterol acyltransferase family protein IPR003386 Lecithin:cholesterol acyltransferase
30213	Solyc11g008560.1	AP2-like ethylene -responsive transcription factor At1g16060 IPR001471 Pathogenesis-related transcriptional factor and ERF, DNA-binding
30214	Solyc11g008570.1	Calcineurin-related phosphoesterase-like IPR004843 Metallophosphoesterase
30215	Solyc11g008580.1	Ariadne-like ubiquitin ligase IPR002867 Zinc finger, C6HC -type
30216	Solyc11g008590.1	UDP-galactose transporter-like protein IPR0072 71 Nucleotide -sugar transporter
30217	Solyc11g008600.1	Genomic DNA chromosome 5 P1 clone MUA2
30218	Solyc11g008610.1	Nucleotide-sugar transporter UDP N -acetylglucosamine-like signal peptide 9 or more transmembrane domains (Fragment) IPR007271 Nucleoti de-sugar transporter
30219	Solyc11g008620.1	Phosphoglycolate phosphatase IPR006349 2 -phosphoglycolate phosphatase, eukaryotic
30220	Solyc11g008630.1	Hydroxycinnamoyl CoA shikimate_quinate hydroxycinnamoyltransferase -like protein (Fragment) IPR003480 Transferase
30221	Solyc11g008640.1	Flowering locus T IPR008914 Phosphatidylethanolamine -binding protein PEBP
30222	Solyc11g008650.1	Flowering locus T1 IPR008914 Phosphatidylethanolamine -binding protein PEBP
30223	Solyc11g008660.1	Flowering locus T IPR008914 Phosphatidylethanolamine -binding protein PEBP
30224	Solyc11g008670.1	At1g65470_F5I14_33 (Fragment)

30225	Solyc11g008680.1	Acyl- IPR005067 Fatty acid desaturase, type 2
30226	Solyc11g008690.1	TO23-3 (Fragment)
30227	Solyc11g008700.1	Splicing factor U2AF-associated protein IPR012677 Nucleotide -binding, alpha-beta plait
30228	Solyc11g008710.1	30S ribosomal protein S5 IPR 000851 Ribosomal protein S5
30229	Solyc11g008720.1	Beta-glucosidase G4 IPR001360 Glycoside hydrolase, family 1
30230	Solyc11g008730.1	U11_U12 small nuclear ribonucleoprotein protein IPR019955 Ubiquitin supergroup
30231	Solyc11g008740.1	Double-strand-break repair protein rad21 IPR006910 Rad21_Rec8 like protein, N -terminal
30232	Solyc11g008750.1	Unknown Protein
30233	Solyc11g008760.1	Pentatricopeptide repeat-containing protein IPR002885 Pentatricopeptide repeat
30234	Solyc11g008770.1	LETM1 and EF -hand domain -containing protein 1, mitochondrial IPR011685 LETM1-like
30235	Solyc11g008780.1	Acetolactate synthase small subunit IPR004789 Acetolactate synthase, small subunit
30236	Solyc11g008790.1	ARV1 IPR007290 Arv1 -like protein
30237	Solyc11g008800.1	Inositol 1 4 5 -trisphosphate 5 -phosphatase-like protein IPR000300 Inositol polyphosphate related phosphatase
30238	Solyc11g008810.1	Beta-hexosaminidase b IPR001540 Glycoside hydrolase, family 20
30239	Solyc11g008820.1	Endoglucanase IPR001701 Glycoside hydrolase, family 9
30240	Solyc11g008830.1	LOB domain protein IPR004883 Lateral organ boundaries, LOB
30241	Solyc11g008840.1	En_Spm-like transposon protein
30242	Solyc11g008850.1	Serine protease IPR015724 Serine endopeptidase DegP2
30243	Solyc11g008860.1	Laccase-2 IPR001117 Multicopper oxidase, type 1
30244	Solyc11g008870.1	Methylenetetrahydrofolate reductase IPR004621 Eukaryotic -type methylenetetrahydrofolate reductase
30245	Solyc11g008880.1	Wiscott-Aldrich syndrome C-terminal IPR000095 PAK -box_P21-Rho-binding
30246	Solyc11g008890.1	Predicted membrane protein (Fragment) IPR001727 Uncharacterised protein family UPF0016
30247	Solyc11g008900.1	Zinc finger CCCH domain -containing protein 66 IPR0005 71 Zinc finger, CCCH -type
30248	Solyc11g008910.1	Cysteine-rich peptide
30249	Solyc11g008920.1	Unknown Protein
30250	Solyc11g008930.1	Lysine ketoglutarate reductase trans -splicing related 1 -like IPR007877 Protein of unknown function DUF707
30251	Solyc11g008940.1	Pentatricopeptide repeat-containing protein IPR002885 Pentatricopeptide repeat
30252	Solyc11g008950.1	Unknown Protein
30253	Solyc11g008960.1	Receptor like kinase, RLK

30254	Solyc11g008970.1	Pentatricopeptide repeat-containing protein IPR002885 Pentatricopeptide repeat
30255	Solyc11g008980.1	Os02g0726600 protein (Fragment)
30256	Solyc11g008990.1	Phage shock protein A PspA IPR007157 PspA_IM30
30257	Solyc11g009000.1	Carboxylesterase bioH IPR000073 Alpha_beta hydrolase fold -1
30258	Solyc11g009010.1	Hydrolase alpha_beta fold family protein IPR000073 Alpha_beta hydrolase fold -1
30259	Solyc11g009020.1	HAD-superfamily hydrolase subfamily IA variant 3 containing protein expressed IPR005834 Haloacid dehalogenase -like hydrolase
30260	Solyc11g009030.1	Ribosomal protein L37
30261	Solyc11g009040.1	EPIDERMAL PATTERNING FACTOR-like protein 1
30262	Solyc11g009050.1	F-box protein-like IPR001810 Cyclin -like F-box
30263	Solyc11g009060.1	Unknown Protein
30264	Solyc11g009070.1	Genomic DNA chromosome 5 P1 clone MQN23
30265	Solyc11g009080.1	Phospho-2-dehydro-3-deoxyheptonate aldolase 1 IPR002480 DAHP synthetase, class II
30266	Solyc11g009090.1	Polyadenylate-binding protein family protein IPR012677 Nucleotide -binding, alpha-beta plait
30267	Solyc11g009100.1	ABC transporter G family member 11 IPR013525 ABC -2 type transporter
30268	Solyc11g010100.1	Ras-related protein Rab-25 IPR015595 Rab11 -related
30269	Solyc11g010110.1	Ribosomal RNA small subunit methyltransferase F_eukaryo tic nucleolar NOL1_Nop2p
30270	Solyc11g010120.1	Peroxidase 17 IPR002016 Haem peroxidase, plant_fungal_bacterial
30271	Solyc11g010130.1	Serine-threonine protein kinase IPR015740 Plant protein serine_threonine kinase -like
30272	Solyc11g010140.1	Unknown Protein
30273	Solyc11g010150.1	Serine-threonine protein kinase IPR017442 Serine_threonine protein kinase -related
30274	Solyc11g010160.1	Cc-nbs-lrr, resistance protein
30275	Solyc11g010170.1	LanC-like protein 2 IPR020464 LanC -like protein, eukaryotic
30276	Solyc11g010180.1	Mitochondrial_chloroplast ribosomal protein L54_L37 (ISS) IPR013870 Ribosomal protein L37, mitochondrial
30277	Solyc11g010190.1	Prohibitin IPR000163 Prohibitin
30278	Solyc11g010200.1	14-3-3 protein beta_alpha-1 IPR 000308 14 -3-3 protein
30279	Solyc11g010210.1	SKIP interacting protein 3 (Fragment)
30280	Solyc11g010220.1	Pentatricopeptide repeat-containing protein IPR002885 Pentatricopeptide repeat
30281	Solyc11g010230.1	Histone H3 IPR000164 Histone H3
30282	Solyc11g010240.1	Protein SCAI
30283	Solyc11g010250.1	Avr9_Cf-9 rapidly elicited protein 75
30284	Solyc11g010260.1	Pentatricopeptide repeat-containing protein IPR002885 Pentatricopeptide repeat
30285	Solyc11g010270.1	Homeobox-leucine zipper protein IP R001356 Homeobox

30286	Solyc11g010280.1	Unknown Protein
30287	Solyc11g010290.1	2-oxoglutarate_malate translocator IPR001898 Sodium_sulphate symporter
30288	Solyc11g010300.1	3 4-dihydroxy-2-butanone 4-phosphate synthase IPR000422 DHBP synthase RibB
30289	Solyc11g010310.1	ATP dependent RNA helicase IPR014001 DEAD -like helicase, N-terminal
30290	Solyc11g010320.1	AT2G46550 protein (Fragment)
30291	Solyc11g010330.1	RING finger protein IPR018957 Zinc finger, C3HC4 RING -type
30292	Solyc11g010340.1	BHLH transcription factor IPR001092 Basic helix -loop-helix dimerisation region bHLH
30293	Solyc11g010350.1	Glucan 1 3-beta-glucosidase IPR001547 Glycoside hydrolase, family 5
30294	Solyc11g010360.1	Plant-specific domain TIGR01570 family protein IPR0 06460 Protein of unknown function DUF617, plant
30295	Solyc11g010370.1	At4g40080-like protein (Fragment) IPR013809 Epsin -like, N-terminal
30296	Solyc11g010380.1	Mate efflux family protein IPR002528 Multi antimicrobial extrusion protein MatE
30297	Solyc11g010390.1	Unknown Protein
30298	Solyc11g010400.1	1-aminocyclopropane-1-carboxylate oxidase IPR005123 Oxoglutarate and iron -dependent oxygenase
30299	Solyc11g010410.1	1-aminocyclopropane-1-carboxylate oxidase IPR005123 Oxoglutarate and iron -dependent oxygenase
30300	Solyc11g010420.1	Ovarian cancer-associated gene 2 protein homolog IPR005645 Protein of unknown function DUF341
30301	Solyc11g010430.1	Ovarian cancer-associated gene 2 protein homolog IPR005645 Protein of unknown function DUF341
30302	Solyc11g010440.1	FIP1 IPR004182 GRAM
30303	Solyc11g010450.1	Phosphofructokinase family protein IPR012004 Pyrophosphate -dependent phosphofructokinase TP0108
30304	Solyc11g010460.1	Cyclin D2 IPR015451 Cyclin D
30305	Solyc11g010470.1	14-3-3 protein beta_alpha IPR000308 14 -3-3 protein
30306	Solyc11g010480.1	Threonine endopeptidase
30307	Solyc11g010490.1	Genomic DNA chromosome 5 P1 clone MNA5
30308	Solyc11g010500.1	Mitochondrial carrier family IPR001993 Mitochondrial substrate carrier
30309	Solyc11g010510.1	Thymidylate synthase (Fragment) IPR001498 Uncharacterised protein family UPF0029, N-terminal
30310	Solyc11g010520.1	Unknown Protein IPR010800 Glycine rich
30311	Solyc11g010530.1	Unknown Protein IPR010800 Glycine rich
30312	Solyc11g010540.1	DnaJ domain containing protein expressed IPR015609 Molecular chaperone, heat shock protein, Hsp40, DnaJ
30313	Solyc11g010550.1	GPI ethanolamine phosphate transferase IPR017850 Alkaline -phosphatase-like, core

30314	Solyc11g010560.1	Kinesin-like protein IPR0 01752 Kinesin, motor region
30315	Solyc11g010570.1	MADS box transcription factor IPR002100 Transcription factor, MADS -box IPR002487 Transcription factor, K -box
30316	Solyc11g010580.2	Unknown Protein
30317	Solyc11g010590.1	Protein-tyrosine phosphata se-like member A IPR007482 Protein -tyrosine phosphatase-like, PTPLA
30318	Solyc11g010600.1	Os01g0841200 protein (Fragment) IPR004348 Protein of unknown function DUF246, plant
30319	Solyc11g010610.1	Small conductance mechanosensitive (MscS -family) ion channel IPR006685 Mechanosensitive ion channel MscS
30320	Solyc11g010620.1	Unknown Protein
30321	Solyc11g010630.2	Unknown Protein
30322	Solyc11g010650.2	Unknown Protein IPR000868 Isochorismatase -like
30323	Solyc11g010660.1	SGT1 protein IPR010770 SGT1
30324	Solyc11g010670.1	Unknown Protein
30325	Solyc11g010680.2	Unknown Protein
30326	Solyc11g010690.1	Nucleobase ascorbate transporter IPR006043 Xanthine_uracil_vitamin C permease
30327	Solyc11g010700.1	Receptor-like protein kinase IPR001245 T yrosine protein kinase
30328	Solyc11g010710.1	AP2-like ethylene -responsive transcription factor At1g16060 IPR001471 Pathogenesis-related transcriptional factor and ERF, DNA-binding
30329	Solyc11g010720.1	Genomic DNA chromosome 5 TAC clone K21L13 IPR01 1990 Tetratricopeptide -like helical
30330	Solyc11g010730.1	Receptor-like kinase IPR002290 Serine_threonine protein kinase
**30331**	**Solyc11g010740.1**	**UDP-glucosyltransferase IPR002213 UDP -glucuronosyl_UDP-glucosyltransferase**
**30332**	**Solyc11g010750.1**	**UDP-glucosyltransferase IPR002213 UDP -glucuronosyl_UDP-glucosyltransferase**
**30333**	**Solyc11g010760.1**	**UDP-glucosyltransferase IPR002213 UDP -glucuronosyl_UDP-glucosyltransferase**
30334	Solyc11g010770.1	Unknown Protein
**30335**	**Solyc11g010780.1**	**Glucosyltransferase IPR002213 UDP -glucuronosyl_UDP-glucosyltransferase**
**30336**	**Solyc11g010790.1**	**Glucosyltransferase IPR002213 UDP -glucuronosyl_UDP-glucosyltransferase**
**30337**	**Solyc11g010800.1**	**Anthocyanidin 3 -O-glucosyltransferase IPR002213 UDP -glucuronosyl_UDP-glucosyltransferase**
**30338**	**Solyc11g010810.1**	**Glucosyltransferase IPR002213 UDP -glucuronosyl_UDP-glucosyltransferase**
30339	Solyc11g010830.1	Unknown Protein
30340	Solyc11g010840.1	Poly(RC) binding protein 3 IPR018111 K Homology, type 1, subgroup

30341	Solyc11g010850.1	1-deoxy-D-xylulose 5-phosphate synthase 2 IPR005477 Deoxyxylulose -5-phosphate synthase
30342	Solyc11g010860.1	RING-finger protein like IPR018957 Zinc finger, C3HC4 RING -type
30343	Solyc11g010870.1	Unknown Protein
30344	Solyc11g010880.1	Unknown Protein
30345	Solyc11g010890.1	Exostosin family protein IPR004263 Exostosin -like
30346	Solyc11g010900.1	RRNA methyltransferase IPR001537 tRNA_rRNA methyltransferase, SpoU
30347	Solyc11g010910.1	WD repeat protein IPR020472 G -protein beta WD-40 repeat, region
30348	Solyc11g010920.1	Kinesin IPR001752 Kinesin, motor region
30349	Solyc11g010930.1	HVA22-like protein e IPR004345 TB2_DP1 and HVA22 related protein
30350	Solyc11g010940.1	Dof zinc finger protein 6 IPR003851 Zinc finger, Dof -type
30351	Solyc11g010950.1	Elongator complex protein 4 IPR008728 Elongator complex protein 4
30352	Solyc11g010960.1	Alcohol dehydrogenase IPR002085 Alcohol dehydrogenase superfamily, zinc -containing
30353	Solyc11g010980.1	Alcohol dehydrogenase IPR002085 Alcohol dehydrogenase superfamily, zinc -containing
30354	Solyc11g010990.1	Alcohol dehydrogenase IPR002085 Alcohol dehydrogenase superfamily, zinc -containing
30355	Solyc11g011000.1	Cysteine-rich repeat secretory protein 60 IPR002902 Protein of unknow n function DUF26
30356	Solyc11g011010.1	Erwinia induced protein 2
30357	Solyc11g011020.1	Receptor like kinase, RLK
30358	Solyc11g011030.1	Pto-responsive gene 1 protein IPR010399 Tify
30359	Solyc11g011040.1	ADP-ribosylation factor-like protein IPR006 688 ADP -ribosylation factor
30360	Solyc11g011050.1	MYB transcription factor IPR015495 Myb transcription factor
30361	Solyc11g011060.1	AT5g16610_MTG13_5 (Fragment)
30362	Solyc11g011070.1	Genomic DNA chromosome 5 P1 clone MTG13
30363	Solyc11g011080.1	Tir-nbs-lrr, resistance protein
30364	Solyc11g011090.1	Tir-nbs-lrr, resistance protein
30365	Solyc11g011100.1	Unknown Protein
30366	Solyc11g011110.1	GDSL esterase_lipase At5g37690 IPR001087 Lipase, GDSL
30367	Solyc11g011120.1	Calmodulin-binding heat-shock protein IPR002921 Lipase, class 3
30368	Solyc11g011130.1	THO complex subunit 4 IPR012677 Nucleotide -binding, alpha-beta plait
30369	Solyc11g011140.1	50S ribosomal protein L23 IPR013025 Ribosomal protein L25_L23
30370	Solyc11g011150.1	DNA repair protein Rad4 family IPR004583 DNA repair protein Rad4
30371	Solyc11g011160.1	High mobility group protein IPR005818 Histone H1_H5
30372	Solyc11g011170.1	ATA15 protein

30373	Solyc11g011180.1	LRR receptor-like serine_threonine-protein kinase, RLP
30374	Solyc11g011190.1	Exostosin family protein IPR004263 Exostosin -like
30375	Solyc11g011200.1	Post-GPI attachment to proteins factor 3 IPR007217 Per1 -like
30376	Solyc11g011210.1	Gibberellin regulated protein IPR003854 Gibberellin regulated protei n
30377	Solyc11g011220.1	Unknown Protein
30378	Solyc11g011230.1	F-box family protein IPR001810 Cyclin -like F-box
30379	Solyc11g011240.1	Geranylgeranyl pyrophosphate synthase 1 IPR000092 Polyprenyl synthetase
30380	Solyc11g011250.1	Chloride intracel lular channel 6 IPR017933 Glutathione S -transferase_chloride channel, C-terminal
30381	Solyc11g011260.1	GAI-like protein 1 (Fragment) IPR005202 GRAS transcription factor
30382	Solyc11g011270.1	Rhamnogalacturonate lyase IPR010325 Rhamnogalacturonate lyase
30383	Solyc11g011280.1	LG127_30 like gene IPR008979 Galactose -binding like
30384	Solyc11g011290.1	Rhamnogalacturonate lyase IPR010325 Rhamnogalacturonate lyase
30385	Solyc11g011300.1	Rhamnogalacturonate lyase IPR010325 Rhamnogalacturonate l yase
30386	Solyc11g011310.1	Rhamnogalacturonate lyase IPR010325 Rhamnogalacturonate lyase
30387	Solyc11g011320.1	Rhamnogalacturonate lyase IPR010325 Rhamnogalacturonate lyase
30388	Solyc11g011330.1	Cinnamyl alcohol dehydrogenase IPR002085 Alcohol dehydrogenase superfamily, zinc-containing
30389	Solyc11g011340.1	Alcohol dehydrogenase IPR002085 Alcohol dehydrogenase superfamily, zinc - containing
30390	Solyc11g011350.1	Tir-nbs-lrr, resistance protein
30391	Solyc11g011360.1	Nudix hydrolase 4 IPR00 0086 NUDIX hydrolase domain
30392	Solyc11g011370.1	Condensin complex subunit 3 IPR016024 Armadillo -type fold
30393	Solyc11g011380.1	Glutamine synthetase IPR008146 Glutamine synthetase, catalytic region
30394	Solyc11g011390.1	TPR repeat Kinesin light chain Kinesin light chain IPR011990 Tetratricopeptide -like helical
30395	Solyc11g011400.1	Unknown Protein
30396	Solyc11g011410.1	Pentatricopeptide repeat-containing protein IPR002885 Pentatricopeptide repeat
30397	Solyc11g011420.1	Peptidyl-prolyl cis-trans isomerase IPR001179 Peptidyl -prolyl cis -trans isomerase, FKBP-type
30398	Solyc11g011430.1	Ycf2 IPR008543 Chloroplast Ycf2
30399	Solyc11g011440.1	Aspartic proteinase nepenthesin-1 IPR001461 Peptidase A1
30400	Solyc11g011450.1	Glutathione-dependent formaldehyde -activating GFA IPR006913 Glutathione - dependent formaldehyde-activating, GFA
30401	Solyc11g011460.1	ATP-dependent RNA helicase IPR001650 DNA_RNA helicase, C -terminal
30402	Solyc11g011470.1	NADH-ubiquinone oxidoreductase subunit IPR010228 NADH:ubiquinone oxidoreductase, subunit G

30403	Solyc11g011480.1	Nicastrin IPR008710 Nicastrin
30404	Solyc11g011490.1	CTV.12
30405	Solyc11g011500.1	Potassium channel IPR002110 Ankyrin
30406	Solyc11g011510.1	Unknown Protein
30407	Solyc11g011520.1	Unknown Protein
30408	Solyc11g011530.1	Unknown Protein
30409	Solyc11g011540.1	F-box family protein IPR001810 Cyclin -like F-box
30410	Solyc11g011550.1	Unknown Protein
30411	Solyc11g011560.1	PHD finger family protein IPR011011 Zinc finger, FYVE_PHD-type
30412	Solyc11g011570.1	RING finger protein 44 IPR011016 Zinc finger, RING -CH-type
30413	Solyc11g011580.1	FIP1 IPR004182 GRAM
30414	Solyc11g011590.1	FIP1 IPR004182 GRAM
30415	Solyc11g011600.1	GRAM-containing_ABA-responsive protein (Fragment)
30416	Solyc11g011610.1	Fatty acid -binding protein -like protein IPR014878 Region of unknown function DUF1794
30417	Solyc11g011620.1	Fatty acid -binding protein -like protein IPR014878 Region of unknown function DUF1794
30418	Solyc11g011630.1	Auxin-induced SAUR-like protein IPR003676 Auxin responsive SAUR protein
30419	Solyc11g011640.1	Auxin-responsive protein IPR003676 Auxin responsive SAUR protein
30420	Solyc11g011650.1	Auxin-induced SAUR-like protein IPR003676 Auxin responsive SAUR p rotein
30421	Solyc11g011660.1	Auxin-induced SAUR-like protein IPR003676 Auxin responsive SAUR protein
30422	Solyc11g011670.1	Auxin-responsive protein IPR003676 Auxin responsive SAUR protein
30423	Solyc11g011680.1	Auxin-induced SAUR-like protein IPR 003676 Auxin responsive SAUR protein
30424	Solyc11g011690.1	Auxin-responsive protein IPR003676 Auxin responsive SAUR protein
30425	Solyc11g011700.1	Auxin-induced SAUR-like protein IPR003676 Auxin responsive SAUR protein
30426	Solyc11g011710.1	Auxin-responsive protein IPR003676 Auxin responsive SAUR protein
30427	Solyc11g011720.1	Auxin-induced SAUR-like protein IPR003676 Auxin responsive SAUR protein
30428	Solyc11g011730.1	Auxin-induced SAUR-like protein IPR003676 Auxin responsive SAUR protei n
30429	Solyc11g011740.1	Ethylene-responsive transcription factor 2 IPR001471 Pathogenesis -related transcriptional factor and ERF, DNA-binding
30430	Solyc11g011750.1	Ethylene-responsive transcription factor 2 IPR001471 Pathogenesis -related transcriptional factor and ERF, DNA-binding
30431	Solyc11g011760.1	Genomic DNA chromosome 5 P1 clone MQK4
30432	Solyc11g011770.1	Myb family transcription factor -like IPR006447 Myb -like DNA -binding region, SHAQKYF class
30433	Solyc11g011780.1	Nonsense-mediated mRNA decay NMD3 family protein IPR007064 NMD3
30434	Solyc11g011790.1	Importin beta-3 IPR011989 Armadillo -like helical

30435	Solyc11g011800.1	Importin beta IPR011989 Armadillo -like helical
30436	Solyc11g011810.1	Oxidoreductase family protein-binding domain
30437	Solyc11g011820.1	Oxidoreductase
30438	Solyc11g011830.1	Unknown Protein
30439	Solyc11g011840.1	Oxidoreductase family protein-binding domain
30440	Solyc11g011850.1	Galactosylgalactosylxylosylprotein 3 -beta-glucuronosyltransferase 1 IPR0050 27 Glycosyl transferase, family 43
30441	Solyc11g011860.1	Pentatricopeptide repeat-containing protein IPR002885 Pentatricopeptide repeat
30442	Solyc11g011870.1	RLK, Receptor like protein, putative resistance protein with an antifungal domain
30443	Solyc11g011880.1	RLK, Receptor like protein, putative resistance protein with an antifungal domain
30444	Solyc11g011890.1	Zinc finger protein 7 IPR007087 Zinc finger, C2H2 -type
30445	Solyc11g011900.1	Ubiquitin family protein IPR019956 Ubiquitin subgrou p
30446	Solyc11g011910.1	Transmembrane 9 superfamily protein member 1
30447	Solyc11g011920.1	Glutamate decarboxylase IPR010107 Glutamate decarboxylase
30448	Solyc11g011930.1	Isocitrate dehydrogenase IPR004790 Isocitrate dehydrogenase NADP -dependent, eukaryotic
30449	Solyc11g011940.1	Homeobox-leucine zipper protein PROTODERMAL FACTOR 2 IPR002913 Lipid -binding START
30450	Solyc11g011950.1	Unknown Protein
30451	Solyc11g011960.1	UTP-glucose 1 phosphate uridylyltransferase IPR016267 UTP --glucose-1-phosphate uridylyltransferase, subgroup
30452	Solyc11g011970.1	Tyrosine phosphatase family protein IPR020428 Protein -tyrosine phosphatase, dual specificity phosphatase, eukaryotic
30453	Solyc11g011980.1	WD-40 repeat protein IPR020472 G -protein beta WD-40 repeat, region
30454	Solyc11g011990.1	Plastid terminal oxydase

Supplementary Table 2: *UGT91* family genes in *S. lycopersicum* and *S. pennellii*.

S. lycopersicum		*S. pennellii*	
Gene ID in SGN[1]	UGT ID	Gene ID in SGN[1]	UGT ID
Solyc11g010740	*UGT91A6*	*Sopen11g005550*	*UGT91A8*
Solyc11g010750	pseudogene	-	-
Solyc11g010760	*UGT91A7*	*Sopen11g005560*	*UGT91A9*
Solyc11g010780	*UGT91R1*	*Sopen11g005570*	*UGT91R4*
Solyc11g010790	*UGT91R2*	*Sopen11g005590*	pseudogene
Solyc11g010800	pseudogene	-	-
Solyc11g010810	*UGT91R3*	*Sopen11g005580*	pseudogene

[1] Sol Genomics Network (https://solgenomics.net/)

Supplementary Table 3: Primers using in this study.

Target gene ID	Forward (5′-3′)	Reverse (5′-3′)	Objectives
Solyc11g010780 & *Sopen11g005570* (*UGT91R1/R4*)	AGATCGATGCATGGA GAGAGA	GGTAATTGAATCAATT CATCATC	Quantitative RT-PCR
GAPDH	CAAGGATTGGAGAGG TGGAA	CCTGACTGTGAGGTC AACCA	Quantitative RT-PCR
Solyc11g010740	ATGGAAACTCAACTT GTCAAATCC	ACCAACCCCTTTGTCC TGTAG	RT-PCR
Solyc11g010760	GCCACAAAATTTCGTT TATCTCAAC	CTAAATATCGAACTCA ATTCCTTTGC	RT-PCR
Solyc11g010780	CTTCGAAGCTTCCATG AAAGGA	GTATCGAATACGCCAT TTTCTTC	RT-PCR
Solyc11g010790	CTTCCACCAAGTCTTA TTCCC	TTCTCCGAAACCTTCT GGTAG	RT-PCR
Solyc11g010810	TCAGCTCCTGATTTCA TACTC	GTTCTTCGAAACCTTC TGGTAC	RT-PCR
ACTIN	ATGACTCAAATCATGT TTGAGAC	CAGTGAATTCCTTGCT CATAC	RT-PCR
UGT91R1-Target-1	ATTGGGTTTCAAACG GAACCCATT	AAACAATGGGTTCCG TTTGAAACC	gRNA cloning
UGT91R1-Target-2	ATTGTGAAATTCGAAT GACGCCGG	AAACCCGGCGTCATT CGAATTTCA	gRNA cloning
UGT91R1-Seq	CGATTCCACAAGACA ATTGG	AGCAGAAAAGTGTAA CCCAC	Sequencing
UGT91R1-OX	ACTGTTGATAGTCGAA TGGCGGAAAACGGAA	TGTAGTCCATGTCGAC TGCTTTAATCTTTTCA TAG	UGT91R1-OX cloning
