# Supplementary material for: Identification of a tomato UDP-arabinosyltransferase for airborne volatile reception
Source: Nat Commun. 2023 Feb 8;14:677. doi: 10.1038/s41467-023-36381-8 (PMC9908901; doi:10.1038/s41467-023-36381-8)
Supplement: Supplementary file 2 — Descriptions of Additional Supplementary Files [file 41467_2023_36381_MOESM2_ESM.pdf]

Descriptions of Additional Supplementary Files

Supplementary Data 1
Description: Amino acids sequences for Phylogenetic analysis (Fig. 2b)

Supplementary Data 2
Description: Amino acids sequences for Phylogenetic analysis (Supplementary Fig. 5a)

Supplementary Data 3
Description: Synthesis of HexVic (Supplementary Fig. 10)
