# Supplementary material for: Identification of a tomato UDP-arabinosyltransferase for airborne volatile reception
Source: Nat Commun. 2023 Feb 8;14:677. doi: 10.1038/s41467-023-36381-8 (PMC9908901; doi:10.1038/s41467-023-36381-8)
Supplement: Supplementary file 3 — Supplementary Data 1 [file 41467_2023_36381_MOESM3_ESM.pdf]

Supplementary Data 1

Amino acids sequences for Phylogenetic analysis (Fig. 2b)

>tomato UGT91R2
MAENGKKLHIAVFPWLAFGHMIPYLELSKLIAQKGHKISFISTPRNIDRLPKLPPSLIPFF
NFVKLPMPHVEKLPENAEATIDLPYEQVKHLKLAHDALQESIAKFLEDSDIDFILFDFV
SYWLPSIASKFNIPTGYFSIFVAAYLGFTGPVPGLNNNYEIRMTLEELTVSPKWVPFETA
VAFKEFELLRIYEGCKEGEEENFYDISRMYKTFENCDFLLVRSCLEFEPEWLKVVEDIH
PKPVIPVGQLPTTSYEDDNTDIDAWREIKLWLDKQEKGKVIYVAFGSEAKLSQNELTE
LSLGLELSGLPFFWVLRTKRGESDNELIQLPEGFGERTKERGIVYTSWVPQLKILSHDS
VGGFLTHAGWSSIVEAIQFEKPLLLLTFLADQGINARLLEEKKVAYLIPRNDWDGSFT
HNAVVESLYLVLLEKEGEIYRKKIKEVKNLCCDKKRQDDYVENLLRFLQNYKKIKV

>tomato UGT91R3
MAENSKKLHIAVFPWLAFGHMIPYLELSKLIAQKGHKISFISTPRNIDRLPKLPPSLAPF
LNFVKIPLPYVEKLPKNAEATTDLPYEQVKYLKLAHDALKEPMAKFLEDSAPDFILFDF
TSYWLPSIASKFNIPTGYFSIFVAAYLGFTGPVPGLNNNYENRMTLEELTVSPKWVPFE
TAVAFKEFELLRIYEGCKEGEEENFYDISRMYKTFENCDFLLVRSCLEFEPEWLKVVEDI
HPKPVIPVGQLPTTSYEDDNTDIDAWREIKLWLDKQEKGKVIYVAFGSEAKLSQNELT
ELSLGLELSGLPFFWVLRTKRGESDNELIQVPEGFEERTKERGIVYTSWVPQLKILSHD
SVGGFLTHAGWSSIVEAIQFEKPLLLLTFLADQGINARLLEEKKVAYLIPRNDWDGSFT
HKAVVESLYLVLLEKEGEIYQKKIKEVKNLCCDKKRQDDYVENLIRFLQNYKKIKV

>tomato UGT91R1
MAENGKKLHIAVFPWLAFGHMIPYLELSKLIAQKGHKISFISTPRNIDRLPKLPPNLTPF
LNFVKLPMPHVEKLPENAEATIDLPYEQVKYLKLAQDALQESMSKFIEDSDIDFILFDF
TSYWVPSIASKFNIPSGYFSIFIAAFLGFTGPVPGLNNDYEIRMTPEEYTVTPKWVPFET
TVAFKLFEVSRIFEASMKGEEENIADIVRYYRSVENCDFLLVRSCSEFEPEWLKVVGDIH
RKPVFPVGQLPTTPYEDDSTKIDAWREIKLWLDKQEKGKVIYVAFGSEAKPSQNELTE
LSLGLELSGLPFFWVLRIKRGESDDELIQLPEGFEERTKGRGIVCTSWAPQLKILSHDS
VGGFLTHSGWSSVVEAIQFEKSLVLLTFLADQGINARLLEEKKMAYSIPRNDQDGSFT
RDSVAESLNLVLVKEEGFIYREKIKEMKDLFCDKKRQNNYVENLLSFLQDYEKIKA

>Arabidopsis UGT91A1
MTNFKDNDGDGTKLHVVMFPWLAFGHMVPYLELSKLIAQKGHKVSFISTPRNIDRLL
PRLPENLSSVINFVKLSLPVGDNKLPEDGEATTDVPFELIPYLKIAYDGLKVPVTEFLES
SKPDWVLQDFAGFWLPPISRRLGIKTGFFSAFNGATLGILKPPGFEEYRTSPADFMKP
PKWVPFETSVAFKLFECRFIFKGFMAETTEGNVPDIHRVGGVIDGCDVIFVRSCYEYEA
EWLGLTQELHRKPVIPVGVLPPKPDEKFEDTDTWLSVKKWLDSRKSKSIVYVAFGSEA

KPSQTELNEIALGLELSGLPFFWVLKTRRGPWDTEPVELPEGFEERTADRGMVWRG
WVEQLRTLSHDSIGLVLTHPGWGTIIEAIRFAKPMAMLVFVYDQGLNARVIEEKKIGY
MIPRDETEGFFTKESVANSLRLVMVEEEGKVYRENVKEMKGVFGDMDRQDRYVDSF
LEYLVTNR

>Arabidopsis UGT91B1
MAEPKPKLHVAVFPWLALGHMIPYLQLSKLIARKGHTVSFISTARNISRLPNISSDLSVN
FVSLPLSQTVDHLPENAEATTDVPETHIAYLKKAFDGLSEAFTEFLEASKPNWIVYDIL
HHWVPPIAEKLGVRRAIFCTFNAASIIIIGGPASVMIQGHDPRKTAEDLIVPPPWVPFET
NIVYRLFEAKRIMEYPTAGVTGVELNDNCRLGLAYVGSEVIVIRSCMELEPEWIQLLSK
LQGKPVIPIGLLPATPMDDADDEGTWLDIREWLDRHQAKSVVYVALGTEVTISNEEI
QGLAHGLELCRLPFFWTLRKRTRASMLLPDGFKERVKERGVIWTEWVPQTKILSHGS
VGGFVTHCGWGSAVEGLSFGVPLIMFPCNLDQPLVARLLSGMNIGLEIPRNERDGLF
TSASVAETIRHVVVEEEGKIYRNNAASQQKKIFGNKRLQDQYADGFIEFLENPIAGV

>Arabidopsis UGT91C1
MVDKREEVMHVAMFPWLAMGHLLPFLRLSKLLAQKGHKISFISTPRNIERLPKLQSNL
ASSITFVSFPLPPISGLPPSSESSMDVPYNKQQSLKAAFDLLQPPLKEFLRRSSPDWIIYD
YASHWLPSIAAELGISKAFFSLFNAATLCFMGPSSSLIEEIRSTPEDFTVVPPWVPFKSNI
VFRYHEVTRYVEKTEEDVTGVSDSVRFGYSIDESDAVFVRSCPEFEPEWFGLLKDLYR
KPVFPIGFLPPVIEDDDAVDTTWVRIKKWLDKQRLNSVVYVSLGTEASLRHEEVTELA
LGLEKSETPFFWVLRNEPKIPDGFKTRVKGRGMVHVGWVPQVKILSHESVGGFLTHC
GWNSVVEGLGFGKVPIFFPVLNEQGLNTRLLHGKGLGVEVSRDERDGSFDSDSVADS
IRLVMIDDAGEEIRAKAKVMKDLFGNMDENIRYVDELVRFMRSKGSSSSS

>tomato UGT91A6
METQLVKSIANGDESNTKKLHIVMFPWLAFGHIIPFLELSKFIARKGHKISFISSPRNIDR
LPKIPSEFSNSITFVKIPLAKIDGLPKDAEATIDIITSEEMTYLKKAMDGMEKDVTNFLE
NSCPDWIIQDFAQYWLAPISTRLGISRIFYSIINAWFLSFLGSFENMINTNNCTSPPKLE
DFLVPPKWIPFETKATYRLHEARWMVESSQKNVSGVSDMYRNGVTIEGADAIIIRHCH
EFEGQWLKLLEDLHHMPVLPTGLMPPIVESSSHEKNESWISIKDWLDEKPKGSVVYVA
LGSEVTVGQNEINELARGLELSGSPFFWVLRKSSGLGSIDPIVLPDGFEERTKDRGIVW
KSWAPQLKILSHESVGGLLTHCGWSSIIEGLIFGHPLIMLPFLVDQGLNARILQDKGV
GVEVPRNEEGGTYTSDSVVNSVKLVMVENDGKIIREKAKEMSAIFGNKELHDKYIENL
INFLENQLIDS

>tomato UGT91A7
MANGDENTTKKLHIVMFPWLAFGHIIPFLELSKFLALKGHKISFISTPRNIDRLPKIPSE
FSNSITFVKIPLPKIDGLPKDAEATMDITSEEMTYLKKAMDGMENEVTNFLENNCPD
WIIQDFAQYWLAPISTRLGISRIFYSIINAWFLSFLGSFENMINTNNCTSPPKLEDFLVPP
KWIPFETKATYRLHEARWMVESSQKNVSGVSDMYRNGVTIEGADAIIIRHCHEFEGQ
WLKLLEDLHHMPVLPTGLMPPIVESSSHEKNESWISIKEWLDEKPKGSVVYVALGSEV
TVGQNEINELARGLELSGSPFFWVLRKSSGLGSIDPIVLPDGFEERTKDRGIVWKSWAP
QLKILSHESVGGFLTHCGWSSIIEGLMFGHSLIMLPFLVDQGLNARIIEDKGVGIEVPR
NEEDGSYTSNSVANSVKLVMVKNDGKLIREKAKELSSIFSNKELHDKYIENLINFLEDY
KKSQ

>Petunia PhA3G6RT
MENEMKHSNDALHVVMFPFFAFGHISPFVQLANKLSSYGVKVSFFTASGNASRVKSM
LNSAPTTHIVPLTLPHVEGLPPGAESTAELTPASAELLKVALDLMQPQIKTLLSHLKP
HFVLFDFAQEWLPKMANGLGIKTVYYSVVVALSTAFLTCPARVLEPKKYPSLEDMKK
PPLGFPQTSVTSVRTFEARDFLYVFKSFHNGPTLYDRIQSGLRGCSAILAKTCSQMEG
PYIKYVEAQFNKPVFSNRTRSSGPASGKLEEKWATWLNKFEGGTVIYCSFGSETFLTD
DQVKELALGLEQTGLPFFLVLNFPANVDVSAELNRALPEGFLERVKDKGIIHSGWVQ
QQNILAHSSVGCYVCHAGFSSVIEALVNDCQVVMLPQKGDQILNAKLVSGDMEAGV
EINRRDEDGYFGKEDIKEAVEKVMVDVEKDPGKLIRENQKKWKEFLLNKDIQSKYIGN
LVNEMTAMAKVSTT

>morning glory UGT79G16
MGSQATTYHMAMYPWFGVGHLTGFFRLANKLAGKGHRISFLIPKNTQSKLESFNLH
PHLISFVPIVVPSIPGLPPGAETTSDVPFPSTHLLMEAMDKTQNDIEIILKDLKVDVVFY
DFTHWLPSLARKIGIKSVFYSTISPLMHGYALSPERRVVGKQLTEADMMKAPASFPDP
SIKLHAHEARGFTARTVMKFGGDITFFDRIFTAVSESDGLAYSTCREIEGQFCDYIETQ
FQKPVLLAGPALPVPSKSTMEQKWSDWLGKFKEGSVIYCAFGSECTLRKDKFQELLW
GLELTGMPFFAALKPPFETESVEAAIPEELKEKIQGRGIVHGEWVQQQLFLQHPSVGC
FVSHCGWASLSEALVNDCQIVLLPQVGDQIINARIMSVSLKVGVEVEKGEEDGVFSRE
SVCKAVKAVMDEKSEIGREVRGNHDKLRGFLMNADLDSKYMDSFNQKLQDLLG

>Arabidopsis UGT79B1
MGVFGSNESSSMSIVMYPWLAFGHMTPFLHLSNKLAEKGHKIVFLLPKKALNQLEPL
NLYPNLITFHTISIPQVKGLPPGAETNSDVPFFLTHLLAVAMDQTRPEVETIFRTIKPD
LVFYDSAHWIPEIAKPIGAKTVCFNIVSAASIALSLVPSAEREVIDGKEMSGEELAKTPL

GYPSSKVVLRPHEAKSLSFVWRKHEAIGSFFDGKVTAMRNCDAIAIRTCRETEGKFCD
YISRQYSKPVYLTGPVLPGSQPNQPSLDPQWAEWLAKFNHGSVVFCAFGSQPVVNKI
DQFQELCLGLESTGFPFLVAIKPPSGVSTVEEALPEGFKERVQGRGVVFGGWIQQPLV
LNHPSVGCFVSHCGFGSMWESLMSDCQIVLVPQHGEQILNARLMTEEMEVAVEVER
EKKGWFSRQSLENAVKSVMEEGSEIGEKVRKNHDKWRCVLTDSGFSDGYIDKFEQNL
IELVKS

>Arabidopsis UGT79B2
MGGLKFHVLMYPWFATGHMTPFLFLANKLAEKGHTVTFLIPKKALKQLENLNLFPH
NIVFRSVTVPHVDGLPVGTETVSEIPVTSADLLMSAMDLTRDQVEGVVRAVEPDLIFF
DFAHWIPEVARDFGLKTVKYVVVSASTIASMLVPGGELGVPPPGYPSSKVLLRKQDAY
TMKNLESTNTINVGPNLLERVTTSLMNSDVIAIRTAREIEGNFCDYIEKHCRKKVLLT
GPVFPEPDKTRELEERWVKWLSGYEPDSVVFCALGSQVILEKDQFQELCLGMELTGS
PFLVAVKPPRGSSTIQEALPEGFEERVKGRGVVWGEWVQQPLLLSHPSVGCFVSHCG
FGSMWESLLSDCQIVLVPQLGDQVLNTRLLSDELKVSVEVAREETGWFSKESLFDAIN
SVMKRDSEIGNLVKKNHTKWRETLTSPGLVTGYVDNFIESLQDLVSGTNHVSK

>Arabidopsis UGT79B3
MGGLKFHVLMYPWFATGHMTPFLFLANKLAEKGHTVTFLLPKKSLKQLEHFNLFPH
NIVFRSVTVPHVDGLPVGTETASEIPVTSTDLLMSAMDLTRDQVEAVVRAVEPDLIFF
DFAHWIPEVARDFGLKTVKYVVVSASTIASMLVPGGELGVPPPGYPSSKVLLRKQDAY
TMKKLEPTNTIDVGPNLLERVTTSLMNSDVIAIRTAREIEGNFCDYIEKHCRKKVLLT
GPVFPEPDKTRELEERWVKWLSGYEPDSVVFCALGSQVILEKDQFQELCLGMELTGS
PFLVAVKPPRGSSTIQEALPEGFEERVKGRGLVWGGWVQQPLILSHPSVGCFVSHCGF
GSMWESLLSDCQIVLVPQLGDQVLNTRLLSDELKVSVEVAREETGWFSKESLCDAVN
SVMKRDSELGNLVRKNHTKWRETVASPGLMTGYVDAFVESLQDLVSGTTHD

>Arabidopsis UGT79B5
MGSKFHAFMYPWFGFGHMIPYLHLANKLAEKGHRVTFFLPKKAHKQLQPLNLFPDSI
VFEPLTLPPVDGLPFGAETASDLPNSTKKPIFVAMDLLRDQIEAKVRALKPDLIFFDFV
HWVPEMAEEFGIKSVNYQIISAACVAMVLAPRAELGFPPPDYPLSKVALRGHEANVCS
LFANSHELFGLITKGLKNCDVVSIRTCVELEGKLCGFIEKECQKKLLLTGPMLPEPQN
KSGKFLEDRWNHWLNGFEPGSVVFCAFGTQFFFEKDQFQEFCLGMELMGLPFLISV
MPPKGSPTVQEALPKGFEERVKKHGIVWEGWLEQPLILSHPSVGCFVNHCGFGSMW
ESLVSDCQIVFIPQLADQVLITRLLTEELEVSVKVQREDSGWFSKEDLRDTVKSVMDI
DSEIGNLVKRNHKKLKETLVSPGLLSGYADKFVEALEIEVNNTKFS

>Arabidopsis UGT79B6
MGSKFHAFMFPWFGFGHMTAFLHLANKLAEKDHKITFLLPKKARKQLESLNLFPDCI
VFQTLTIPSVDGLPDGAETTSDIPISLGSFLASAMDRTRIQVKEAVSVGKPDLIFFDFA
HWIPEIAREYGVKSVNFITISAACVAISFVPGRSQDDLGSTPPGYPSSKVLLRGHETNSL
SFLSYPFGDGTSFYERIMIGLKNCDVISIRTCQEMEGKFCDFIENQFQRKVLLTGPMLP
EPDNSKPLEDQWRQWLSKFDPGSVIYCALGSQIILEKDQFQELCLGMELTGLPFLVAV
KPPKGSSTIQEALPKGFEERVKARGVVWGGWVQQPLILAHPSIGCFVSHCGFGSMWE
ALVNDCQIVFIPHLGEQILNTRLMSEELKVSVEVKREETGWFSKESLSGAVRSVMDRD
SELGNWARRNHVKWKESLLRHGLMSGYLNKFVEALEKLVQNINLE

>Arabidopsis UGT79B7
MEPKFHAFMFPWFAFGHMIPFLHLANKLAEKGHRVTFLLPKKAQKQLEHHNLFPDSI
VFHPLTVPPVNGLPAGAETTSDIPISLDNLLSKALDLTRDQVEAAVRALRPDLIFFDFA
QWIPDMAKEHMIKSVSYIIVSATTIAHTHVPGGKLGVRPPGYPSSKVMFRENDVHALA
TLSIFYKRLYHQITTGLKSCDVIALRTCKEVEGMFCDFISRQYHKKVLLTGPMFPEPDT
SKPLEERWNHFLSGFAPKSVVFCSPGSQVILEKDQFQELCLGMELTGLPFLLAVKPPR
GSSTVQEGLPEGFEERVKDRGVVWGGWVQQPLILAHPSIGCFVNHCGPGTIWESLVS
DCQMVLIPFLSDQVLFTRLMTEEFEVSVEVPREKTGWFSKESLSNAIKSVMDKDSDIG
KLVRSNHTKLKEILVSPGLLTGYVDHFVEGLQENLI

>Arabidopsis UGT79B8
MEPTFHAFMFPWFAFGHMIPFLHLANKLAEKGHQITFLLPKKAQKQLEHHNLFPDSI
VFHPLTIPHVNGLPAGAETTSDISISMDNLLSEALDLTRDQVEAAVRALRPDLIFFDFA
HWIPEIAKEHMIKSVSYMIVSATTIAYTFAPGGVLGVPPPGYPSSKVLYRENDAHALAT
LSIFYKRLYHQITTGFKSCDIIALRTCNEIEGKFCDYISSQYHKKVLLTGPMLPEQDTSK
PLEEQLSHFLSRFPPRSVVFCALGSQIVLEKDQFQELCLGMELTGLPFLIAVKPPRGSS
TVEEGLPEGFQERVKGRGVVWGGWVQQPLILDHPSIGCFVNHCGPGTIWECLMTD
CQMVLLPFLGDQVLFTRLMTEEFKVSVEVSREKTGWFSKESLSDAIKSVMDKDSDLG
KLVRSNHAKLKETLGSHGLLTGYVDKFVEELQEYLI

>Arabidopsis UGT79B9
MGQNFHAFMFPWFAFGHMTPYLHLANKLAAKGHRVTFLLPKKAQKQLEHHNLFPD
RIIFHSLTIPHVDGLPAGAETASDIPISLGKFLTAAMDLTRDQVEAAVRALRPDLIFFDT
AYWVPEMAKEHRVKSVIYFVISANSIAHELVPGGELGVPPPGYPSSKVLYRGHDAHAL
LTFSIFYERLHYRITTGLKNCDFISIRTCKEIEGKFCDYIERQYQRKVLLTGPMLPEPDN

SRPLEDRWNHWLNQFKPGSVIYCALGSQITLEKDQFQELCLGMELTGLPFLVAVKPP
KGAKTIQEALPEGFEERVKNHGVVWGEWVQQPLILAHPSVGCFVTHCGFGSMWESL
VSDCQIVLLPYLCDQILNTRLMSEELEVSVEVKREETGWFSKESLSVAITSVMDKDSEL
GNLVRRNHAKLKEVLVSPGLLTGYTDEFVETLQNIVNDTNLE

>Arabidopsis UGT79B10
MGQTFHAFMFPWFAFGHMTPYLHLANKLAERGHRITFLIPKKAQKQLEHLNLFPDSI
VFHSLTIPHVDGLPAGAETFSDIPMPLWKFLPPAIDLTRDQVEAAVSALSPDLILFDIAS
WVPEVAKEYRVKSMLYNIISATSIAHDFVPGGELGVPPPGYPSSKLLYRKHDAHALLSF
SVYYKRFSHRLITGLMNCDFISIRTCKEIEGKFCEYLERQYHKKVFLTGPMLPEPNKGK
PLEDRWSHWLNGFEQGSVVFCALGSQVTLEKDQFQELCLGIELTGLPFFVAVTPPKG
AKTIQDALPEGFEERVKDRGVVLGEWVQQPLLLAHPSVGCFLSHCGFGSMWESIMSD
CQIVLLPFLADQVLNTRLMTEELKVSVEVQREETGWFSKESLSVAITSVMDQASEIGN
LVRRNHSKLKEVLVSDGLLTGYTDKFVDTLENLVSETKRE

>Arabidopsis UGT79B11
MGQKIHAFMFPWFAFGHMTPYLHLGNKLAEKGHRVTFLLPKKAQKQLEHQNLFPH
GIVFHPLVIPHVDGLPAGAETASDIPISLVKFLSIAMDLTRDQIEAAIGALRPDLILFDLA
HWVPEMAKALKVKSMLYNVMSATSIAHDLVPGGELGVAPPGYPSSKALYREHDAHA
LLTFSGFYKRFYHRFTTGLMNCDFISIRTCEEIEGKFCDYIESQYKKKVLLTGPMLPEP
DKSKPLEDQWSHWLSGFGQGSVVFCALGSQTILEKNQFQELCLGIELTGLPFLVAVK
PPKGANTIHEALPEGFEERVKGRGIVWGEWVQQPSWQPLILAHPSVGCFVSHCGFGS
MWESLMSDCQIVFIPVLNDQVLTTRVMTEELEVSVEVQREETGWFSKENLSGAIMSL
MDQDSEIGNQVRRNHSKLKETLASPGLLTGYTDKFVDTLENLVNEQGYIS

>tomato NSGT1
MERIKENSPSILLFPWLGFGHVNPFLALAKKLSKMNFHIYFLSTPIILKSIKETLDKNST
NYNQSIQLVEFHLPYLHELPPHYHTTKDLPPHLNSTLIQAFQMASSKFPSIIETLKPNLI
IYDGFQPWVATMASSYNIHAIMFYVSSTSGLAYLYHQFLHGSSSLTSFPFSSIYLHDHEI
KKLGIQPIKPRDEKAFAYIILESFEQSHNIVLLNTCREIEGKYIDYVSTIGKKELIPIGPLIR
EAMIDEEEDWGTIQSWLDKKDQLSCVYVSFGSESFLSKQEIEEIAKGLELSKVNFIWTI
KFPKGVNKTIEEMVPQGFLESTKGKGMVIEGWAPQSLILNHSSIGGFITHCGWNSILES
MSFGIPIIAMPMNHDQPLNSRLVEELGIGVEILRGENGKIMKEEVAKGIRKVIEEKPRK
QIHLKAMQLSEKIKLKAIDEGVKKLLKLLY

>tomato NSGT2

MERIKENSPSILLFPWLGFGHVNPFLALAKKLSKMNFHIYFLSTPIILKSIKETLDKNST
NYNQSIQLVEFHLPYLHELPPHYHTTKDLPPHLNSTLIQAFQMASSKFPSIIETLKPNLI
IYDGFQPWVATMASSYNIHAIMFYVSSTSGLAYLYHQFLYGSSSLTSFPFLHDYEIKKL
DMKPIKPRDEKAFGYVVLKSFEKSHNIVLLNTCREIEGKYIDYVSTIGKKELIQIGPLIRE
ATLGEEENLGTIQSWLDNKDHISCVYVSFGSEFFMSKQEIEEIAKGLELSKVSFIWTIKF
PKGVNTTIEEMVPQGFLESTKGKGMVIEGWVPQSLILNHSSIGGFVTHCGWNSMLES
MSFGIPLIAMPMNHDQPLNSRLVEELGIGVEILRGENGEIMKEEVAKGIKKVVEDNTR
KQVNLKAMELSEKIKFKGEKAIDEGVKKGLKLLC

>pomelo CmF7G2RT
MDTKHQDKPSILMLPWLAHGHIAPHLELAKKLSQKNFHIYFCSTPNNLQSFGRNVEK
NFSSSIQLIELQLPNTFPELPSQNQTTKNLPPHLIYTLVGAFEDAKPAFCNILETLKPTL
VMYDLFQPWAAEAAYQYDIAAILFLPLSAVACSFLLHNIVNPSLKYPFFESDYQDRESK
NINYFLHLTANGTLNKDRFLKAFELSCKFVFIKTSREIESKYLDYFPSLMGNEIIPVGPLI
QEPTFKEDDTKIMDWLSQKEPRSVVYASFGSEYFPSKDEIHEIASGLLLSEVNFIWAFR
LHPDEKMTIEEALPQGFAEEIERNNKGMIVQGWVPQAKILRHGSIGGFLSHCGWGSV
VEGMVFGVPIIGVPMAYEQPSNAKVVVDNGMGMVVPRDKINQRLGGEEVARVIKHV
VLQEEAKQIRRKANEISESMKKIGDAEMSVVVEKLLQLVKKSE

>tomato GAME18
MGNMRIVMLPYLAYGHITPFLELAKKLSNRGFSIHICSSPINLSFIKAKIPEKYSSSIHLVE
LHLPNLPELPPHHHTTNGLPNHLKQTLFKTLKMTKPQLHQILSDLKPDFFIYDIMLL
WSAVVASSLNIPSLRFYTVNAAIFSYFFHFYFNPGEEFPFPALYMRDYELAKMTHEVAD
DAEVEVDRDKVTESDKFVLVHSTKSIDGKYMDYLCGTGQAKVVPIGTESPEDGVGD
VDKIDIELVKWLEKKTEHSTVYVSFGSEYFLSKEEMEEVAYGLEVSGVDFIWVVRYQK
GEQLELPQGFKERIGDRGRIIEGWAPQQRILKHSSIGGFVTHCGWNSTLESIEFGVPIIA
MPMLYDQPLNARLMVENGVAVEVPRDEKGNLDRVNIAEKIKHVIRDETGENLRKKM
NNLGENVRSQREEEMDGVVKVIQLLIDEKKGTL

>Sesami UGT94D1
MDTRKRSIRILMFPWLAHGHISAFLELAKSLAKRNFVIYICSSQVNLNSISKNMSSKDSIS
VKLVELHIPTTILPPPYHTTNGLPPHLMSTLKRALDSARPAFSTLLQTLKPDLVLYDFL
QSWASEEAESQNIPAMVFLSTGAAAISFIMYHWFETRPEEYPFPAIYFREHEYDNFCRF
KSSDSGTSDQLRVSDCVKRSHDLVLIKTFRELEGQYVDFLSDLTRKRFVPVGPLVQEV
GCDMENEGNDIIEWLDGKDRRSTVFSSFGSEYFLSANEIEEIAYGLELSGLNFIWVVRF
PHGDEKIKIEEKLPEGFLERVEGRGLVVEGWAQQRRILSHPSVGGFLSHCGWSSVMEG

VYSGVPIIAVPMHLDQPFNARLVEAVGFGEEVVRSRQGNLDRGEVARVVKKLVMGKS
GEGLRRRVEELSEKMREKGEEEIDSLVEELVTVVRRRERSNLKSENSMKKLNVMDDGE

>daisyUGT94B1
MDSKIDSKTFRVVMLPWLAYSHISRFLVFAKRLTNHNFHIYICSSQTNMQYLKNNLTS
QYSKSIQLIELNLPSSSELPLQYHTTHGLPPHLTKTLSDDYQKSGPDFETILIKLNPHLV
IYDFNQLWAPEVASTLHIPSIQLLSGCVALYALDAHLYTKPLDENLAKFPFPEIYPKNR
DIPKGGSKYIERFVDCMRRSCEIILVRSTMELEGKYIDYLSKTLGKKVLPVGPLVQEASL
LQDDHIWIMKWLDKKEESSVVFVCFGSEYILSDNEIEDIAYGLELSQVSFVWAIRAKTS
ALNGFIDRVGDKGLVIDKWVPQANILSHSSTGGFISHCGWSSTMESIRYGVPIIAMPM
QFDQPYNARLMETVGAGIEVGRDGEGRLKREEIAAVVRKVVVEDSGESIREKAKELGE
IMKKNMEAEVDGIVIENLVKLCEMNN

>tea UGT94P1
MDSKKSKMNVLMLPWLAQGHITPFLELAKKLTNKNFHIYFCSTPINLISIKKRITDKYS
LSIELVEIHLPSSPELPPHYHTTNGLPIHLNSTLKTAFEMASTSFSTILNTLSPDLVIYDV
SPSWAQSTALSFDIPAVQLMITGATVASFGQHMIKHCGSVEFPFPAIKLQGFHETQFR
HFVETVVKEYNDKQVASVNDQPSCNFMLYNTFRELEGKYIDYLPVIGEKKVVPVGPL
VQGIDDDENEHSEIIQWLDNKGEYSTLFVSFGSEYFMSKEEIEEIAHGLELSMVNFIWV
VRFPEVEKVELEEALSKGFIDRVGERGLVVEGWAPQARILTHSSTGGFVSHCGWNSVL
ESLKFGVPMVAIPMQYEQPLNAKLVEEVGVAAEVNRDINGRLNREEIAQVIRKVVVEK
SGEDIRIKARIFGDKIRMKGDEEIDEAVEVLLQLCKDVKLLKN

>Arabidopsis UGT72B2
MAEANTPHIAIMPSPGMGHLIPFVELAKRLVQHDCFTVTMIISGETSPSKAQRSVLNSL
PSSIASVFLPPADLSDVPSTARIETRAMLTMTRSNPALRELFGSLSTKKSLPAVLVVDM
FGADAFDVAVDFHVSPYIFYASNANVLSFFLHLPKLDKTVSCEFRYLTEPLKIPGCVPIT
GKDFLDTVQDRNDDAYKLLLHNTKRYKEAKGILVNSFVDLESNAIKALQEPAPDKPT
VYPIGPLVNTSSSNVNLEDKFGCLSWLDNQPFGSVLYISFGSGGTLTCEQFNELAIGLA
ESGKRFIWVIRSPSEIVSSSYFNPHSETDPFSFLPIGFLDRTKEKGLVVPSWAPQVQILAH
PSTCGFLTHCGWNSTLESIVNGVPLIAWPLFAEQKMNTLLLVEDVGAALRIHAGEDGI
VRREEVVRVVKALMEGEEGKAIGNKVKELKEGVVRVLGDDGLSSKSFGEVLLKWKTH
QRDINQETSH

>Arabidopsis UGT72B3
MADGNTPHVAIIPSPGIGHLIPLVELAKRLLDNHGFTVTFIIPGDSPPSKAQRSVLNSLP

SSIASVFLPPADLSDVPSTARIETRISLTVTRSNPALRELFGSLSAEKRLPAVLVVDLFGT
DAFDVAAEFHVSPYIFYASNANVLTFLLHLPKLDETVSCEFRELTEPVIIPGCVPITGKD
FVDPCQDRKDESYKWLLHNVKRFKEAEGILVNSFVDLEPNTIKIVQEPAPDKPPVYLI
GPLVNSGSHDADVNDEYKCLNWLDNQPFGSVLYVSFGSGGTLTFEQFIELALGLAES
GKRFLWVIRSPSGIASSSYFNPQSRNDPFSFLPQGFLDRTKEKGLVVGSWAPQAQILTH
TSIGGFLTHCGWNSSLESIVNGVPLIAWPLYAEQKMNALLLVDVGAALRARLGEDGV
VGREEVARVVKGLIEGEEGNAVRKKMKELKEGSVRVLRDDGFSTKSLNEVSLKWKAH
QRKIDQEQESFL

>Arabidopsis_UGT72D1
MDQPHALLVASPGLGHLIPILELGNRLSSVLNIHVTILAVTSGSSSPTETEAIHAAAART
ICQITEIPSVDVDNLVEPDATIFTKMVVKMRAMKPAVRDAVKLMKRKPTVMIVDFLG
TELMSVADDVGMTAKYVYVPTHAWFLAVMVYLPVLDTVVEGEYVDIKEPLKIPGCK
PVGPKELMETMLDRSGQQYKECVRAGLEVPMSDGVLVNTWEELQGNTLAALREDE
ELSRVMKVPVYPIGPIVRTNQHVDKPNSIFEWLDEQRERSVVFVCLGSGGTLTFEQTV
ELALGLELSGQRFVWVLRRPASYLGAISSDDEQVSASLPEGFLDRTRGVGIVVTQWAP
QVEILSHRSIGGFLSHCGWSSALESLTKGVPIIAWPLYAEQWMNATLLTEEIGVAVRTS
ELPSERVIGREEVASLVRKIMAEEDEEGQKIRAKAEEVRVSSERAWSKDGSSYNSLFEW
AKRCYLVP

>Arabidopsis UGT79B4
MGSKFHAFLYPWFGFGHMIPYLHLANKLAEKGHRVTFLAPKKAQKQLEPLNLFPNSI
HFENVTLPHVDGLPVGAETTADLPNSSKRVLADAMDLLREQIEVKIRSLKPDLIFFDF
VDWIPQMAKELGIKSVSYQIISAAFIAMFFAPRAELGSPPPGFPSSKVALRGHDANIYSL
FANTRKFLFDRVTTGLKNCDVIAIRTCAEIEGNLCDFIERQCQRKVLLTGPMFLDPQG
KSGKPLEDRWNNWLNGFEPSSVVYCAFGTHFFFEIDQFQELCLGMELTGLPFLVAVM
PPRGSSTIQEALPEGFEERIKGRGIVWGGWVEQPLILSHPSIGCFVNHCGFGSMWESL
VSDCQIVFIPQLVDQVLTTRLLTEELEVSVKVKRDEITGWFSKESLRDTVKSVMDKNS
EIGNLVRRNHKKLKETLVSPGLLSSYADKFVDELENHIHSKN

>Arabidopsis UGT72E1
MFASPGMGHIIPVIELGKRLAGSHGFDVTIFVLETDAASAQSQFLNSPGCDAALVDIV
GLPTPDISGLVDPSAFFGIKLLVMMRETIPTIRSKIEEMQHKPTALIVDLFGLDAIPLGG
EFNMLTYIFIASNARFLAVALFFPTLDKDMEEEHIIKKQPMVMPGCEPVRFEDTLETFL
DPNSQLYREFVPFGSVFPTCDGIIVNTWDDMEPKTLKSLQDPKLLGRIAGVPVYPIGP
LSRPVDPSKTNHPVLDWLNKQPDESVLYISFGSGGSLSAKQLTELAWGLEMSQQRFV

WVVRPPVDGSACSAYLSANSGKIRDGTPDYLPEGFVSRTHERGFMVSSWAPQAEILA
HQAVGGFLTHCGWNSILESVVGGVPMIAWPLFAEQMMNATLLNEELGVAVRSKKLP
SEGVITRAEIEALVRKIMVEEEGAEMRKKIKKLKETAAESLSCDGGVAHESLSRIADESE
HLLERVRCMARGA

>Arabidopsis UGT72B1
MEESKTPHVAIIPSPGMGHLIPLVEFAKRLVHLHGLTVTFVIAGEGPPSKAQRTVLDSL
PSSISSVFLPPVDLTDLSSSTRIESRISLTVTRSNPELRKVFDSFVEGGRLPTALVVDLFG
TDAFDVAVEFHVPPYIFYPTTANVLSFFLHLPKLDETVSCEFRELTEPLMLPGCVPVA
GKDFLDPAQDRKDDAYKWLLHNTKRYKEAEGILVNTFFELEPNAIKALQEPGLDKPP
VYPVGPLVNIGKQEAKQTEESECLKWLDNQPLGSVLYVSFGSGGTLTCEQLNELALG
LADSEQRFLWVIRSPSGIANSSYFDSHSQTDPLTFLPPGFLERTKKRGFVIPFWAPQAQ
VLAHPSTGGFLTHCGWNSTLESVVSGIPLIAWPLYAEQKMNAVLLSEDIRAALRPRAG
DDGLVRREEVARVVKGLMEGEEGKGVRNKMKELKEAACRVLKDDGTSTKALSLVAL
KWKAHKKELEQNGNH

>Arabidopsis UGT72C1
MELHGALVASPGMGHAVPILELGKHLLNHHGFDRVTVFLVTDDVSRSKSLIGKTLME
EDPKFVIRFIPLDVSGQDLSGSLLTKLAEMMRKALPEIKSSVMELEPRPRVFVVDLLGT
EALEVAKELGIMRKHVLVTTSAWFLAFTVYMASLDKQELYKQLSSIGALLIPGCSPVKF
ERAQDPRKYIRELAESQRIGDEVITADGVFVNTWHSLEQVTIGSFLDPENLGRVMRGV
PVYPVGPLVRPAEPGLKHGVLDWLDLQPKESVVYVLLGVVGALTFEQTNELAYGLEL
TGHRFVWVVRPPAEDDPSASMFDKTKNETEPLDFLPNGFLDRTKDIGLVVRTWAPQ
EEILAHKSTGGFVTHCGWNSVLESIVNGVPMVAWPLYSEQKMNARMVSGELKIALQI
NVADGIVKKEVIAEMVKRVMDEEEGKEMRKNVKELKKTAEEALNMTHIPSAYFT

>Arabidopsis UGT72E3
MHITKPHAAMFSSPGMGHVLPVIELAKRLSANHGFHVTVFVLETDAASVQSKLLNST
GVDIVNLPSPDISGLVDPNAHVVTKIGVIMREAVPTLRSKIVAMHQNPTALIIDLFGTD
ALCLAAELNMLTYVFIASNARYLGVSIYYPTLDEVIKEEHTVQRKPLTIPGCEPVRFEDI
MDAYLVPDEPVYHDLVRHCLAYPKADGILVNTWEEMEPKSLKSLQDPKLLGRVARV
PVYPVGPLCRPIQSSTTDHPVFDWLNKQPNESVLYISFGSGGSLTAQQLTELAWGLEE
SQQRFIWVVRPPVDGSSCSDYFSAKGGVTKDNTPEYLPEGFVTRTCDRGFMIPSWAP
QAEILAHQAVGGFLTHCGWSSTLESVLCGVPMIAWPLFAEQNMNAALLSDELGISVR
VDDPKEAISRSKIEAMVRKVMAEDEGEEMRRKVKKLRDTAEMSLSIHGGGSAHESLCR
VTKECQRFLECVGDLGRGA

>Arabidopsis UGT72E2
MHITKPHAAMFSSPGMGHVIPVIELGKRLSANNGFHVTVFVLETDAASAQSKFLNST
GVDIVKLPSPDIYGLVDPDDHVVTKIGVIMRAAVPALRSKIAAMHQKPTALIVDLFGT
DALCLAKEFNMLSYVFIPTNARFLGVSIYYPNLDKDIKEEHTVQRNPLAIPGCEPVRFE
DTLDAYLVPDEPVYRDFVRHGLAYPKADGILVNTWEEMEPKSLKSLLNPKLLGRVAR
VPVYPIGPLCRPIQSSETDHPVLDWLNEQPNESVLYISFGSGGCLSAKQLTELAWGLE
QSQQRFVWVVRPPVDGSCCSEYVSANGGGTEDNTPEYLPEGFVSRTSDRGFVVPSW
APQAEILSHRAVGGFLTHCGWSSTLESVVGGVPMIAWPLFAEQNMNAALLSDELGIA
VRLDDPKEDISRWKIEALVRKVMTEKEGEAMRRKVKKLRDSAEMSLSIDGGGLAHESL
CRVTKECQRFLERVVDLSRGA

>tomato UGT5
MAQIPHIAILPSPGMGHLIPLVEFAKRIFLHHHFSVSLILPTDGPISNAQKIFLNSLPSSM
DYHLLPPVNFDDLPEDVKIETRISLTVSRSLTSLRQVLESIIESKKTVALVVDLFGTDAF
DVAIDLKISPYIFFPSTAMGLSLFLHLPNLDETVSCEYRDLPDPIQIPGCTPIHGKDLLD
PVQDRNDESYKWLLHHAKRYGMAEGIIVNSFKELEGGAIGALQKDEPGKPTVYPVGP
LIQMDSGSKVDGSECMTWLDEQPRGSVLYISYGSGGTLSHEQLIEVAAGLEMSEQRF
LWVVRCPNDKIANATFFNVQDSTNPLEFLPKGFLERTKGFGLVLPNWAPQARILSHE
STGGFLTHCGWNSTLESVVHGVPLIAWPLYAEQKMNAVMLSEDIKVALRPKVNEEN
GIVGRLEIAKVVKGLMEGEEGKGVRSRMRDLKDAAAKVLSEDGSSTKALAELATKLRK
KCQMIDVANH

>grapeGT16
MGDKPHVVCIPFPAQGHIKPMLKLAKLLHYRGFHITFVNTEFNHKRLLRSRGPHALD
GMPGFCFESIPDGLPPVDADATQHIPSLCESTPKSCLIPFQQLIAKLNDAPSSNVPPVT
CIVSDGSMCFTLKASEELGIPNVLFWTTSACGFMAYKQFRPLIDGVLVPLKDLSYLTN
GYLETIIDWVPGMKNMRLRDFPSFIRTRDPSDHFMLDFIIDTTDSASKASGLILNTFHA
LEHDVLNPLSSMFPTICTVGPLPLLLNQIPDDNSIESNLWREETECLQWLNSKQPNSV
VYVNFGSITVMTPEQLVEFAWGLANSHKPFLWIIRPDLVVGDSVILPPEFVNETIQRGL
MAGWCPQEKVLNHPSVGGFLTHSGWNSTIESICAGVPMICWPFFAEQQTNCRYACT
EWGVGMEIDNNVERDEVEKLVKELMEGEKGKSMKKAAMEWRTKAEEATAPCGSSYL
NLDKLVDILLTKP

>Kiwi AdTG4
MGSAGMPEKPHAVCLPYPAQGHITPMLKLAKLLHSKGFHVTFVNTEFNHKRLLKSR

GPDSLTGLSSFRFETIPDGLPESDLDATQFIPSLCESTRKNCLGPFRQLLGKLNNTVSS
GVPPVSCVVSDGVMSFSLDAAEELGIPQVLFWTTSVCGFMAYVHYRNLIEKGYTPLK
DVSYVTNGYLDTVIDWIPGMEGIRLKDLPSFLRTTDPNDIMLDFVLSETKNTHRSSAII
FNTFDKLEHQVLEPLASMFPPIYTIGPLNLLMNQIKEESLKMIGSNLWKEEPMCIEWL
NSKEPKSVVYVNFGSITVMTPNQLVEFAWGLANSNQSFLWIIRPDLVVGESAVLPPEF
VAVTKERGMLASWAPQEEVLAHSSVGGFLTHCGWNSTLESISSGVAVVCWPFFAEQQ
TNCWYCCGELGIGMEIDSDVKREEVERLVRELMVGEKGKEMKERAMGWKRLAEEAT
QSSSGSSFLNLDKLVHQVLLSPRP

>grape GT14
MGSMEKPHAVCIPYPAQGHINPMLKVAKLLHFRGFRITFVNTEFNHTRLLKAQGPNS
LNGLPTFQFETIPDGLPPSNVDATQDIPSLCASTKKNCLAPFRRLLAKLNDRGPPVTC
IFSDAVMSFTLDAAQELGIPDLLLWTASACGFMAYVQYRSLIDKGFTPLKDESYLTNG
YLDTVVDWIPGMKGIRLKDLPSFIRTTDPDDIMLDFAMGELERARKASAIIFNTFDAL
EQEVLDAIAPMYPPIYTIGPLQLLPDQIHDSELKLIGSNLWKEEPECLKWLDSKEPNSV
VYVNYGSITVMTPQQLIEFAWGLANSNQSFLWILRPDLVSGESAILPPEFVAETEDRGL
LAGWCPQEQVLTHQAIGGFLTHNGWNSTIEGLCAGVPMICWPFFAEQQTNCRYCC
TEWGVGMEIDSDVKRDEVAKLVRELMVGEKGKVMKKKTMEWKHRAEVATTGPDG
SSYLNLEKIFEQVLL

>tea UGT85K11
MGSRKQPHAVCVPFPAQGHINPMMQLAKLLHSRGFYITFVNTEFNHRRLLQSKGPEF
LKGCADFQFESIPDGLPPSDRDATQDPPTLCIAMRDNCLDPFRVLLKKLNNNNNSIA
SRQVPGVTCVVSDGAMNFAMKAAEEAGIPEVQFWTASACGFMGYLHYPQLVQRGIF
PFKDESFQSDGSLDTTIDWIPGMRNIRLKDMPSFIRTTDPNDILFNYLSEEVQNCLKAS
AIIFNTFDTLEHQVLQAIASKFHNIYTIGPLSLLSKQVIDGEFKSLNSSLWKEDTKCLQ
WLDTKEPNSVVYVNYGSITVMTDQHLKEFAWGLANSKHPFLWIVRPDIVMGDSAILP
EHFVEETKDRGLLVSWCPQEQVLSHPSIGVFLTHCGWNSTLESICGGVPIICWPFFAE
QQTNCRYACTEWGIGMEVNHDVKRNEIVALINEMLEGDKGKQMRKKALKLKKEAEE
ATDVGGLSYNNFDRLIKEALHYCEQY

>Arabidopsis UGT85A7
MESHVVHNAQKPHVVCVPYPAQGHINPMLKVAKLLYAKGFHVTFVNTLYNHNRLLR
SRGPNALDGFPSFRFESIPDGLPETDGDRTQHTPTVCMSIEKNCLAPFKEILRRINDK
DDVPPVSCIVSDGVMSFTLDAAEELGVPEVIFWTNSACGFMTILHFYLFIEKGLSPFKD
ESYMSKEHLDTVIDWIPSMKNLRLKDIPSYIRTTNPDNIMLNFLIREVERSKRASAIILN

TFDELEHDVIQSMQSILPPVYSIGPLHLLVKEEINEASEIGQMGLNLWREEMECLDWL
DTKTPNSVLFVNFGCITVMSAKQLEEFAWGLAASRKEFLWVIRPNLVVGEAMVVLPQ
EFLAETIDRRMLASWCPQEKVLSHPAIGGFLTHCGWNSTLESLAGGVPMICWPCFSE
QPTNCKFCCDEWGVGIEIGKDVKREEVETVVRELMDGEKGKKLREKAEEWRRLAEEA
TRYKHGSSVMNLETLIHKVFLENLR

>Arabidopsis UGT85A2
MGSHVAQKQHVVCVPYPAQGHINPMMKVAKLLYAKGFHITFVNTVYNHNRLLRSRG
PNAVDGLPSFRFESIPDGLPETDVDVTQDIPTLCESTMKHCLAPFKELLRQINARDDV
PPVSCIVSDGCMSFTLDAAEELGVPEVLFWTTSACGFLAYLYYYRFIEKGLSPIKDESYL
TKEHLDTKIDWIPSMKNLRLKDIPSFIRTTNPDDIMLNFIIREADRAKRASAIILNTFDD
LEHDVIQSMKSIVPPVYSIGPLHLLEKQESGEYSEIGRTGSNLWREETECLDWLNTKAR
NSVVYVNFGSITVLSAKQLVEFAWGLAATGKEFLWVIRPDLVAGDEAMVPPEFLTAT
ADRRMLASWCPQEKVLSHPAIGGFLTHCGWNSTLESLCGGVPMVCWPFFAEQQTN
CKFSRDEWEVGIEIGGDVKREEVEAVVRELMDEEKGKNMREKAEEWRRLANEATEHK
HGSSKLNFEMLVNKVLLGE

>Arabidopsis UGT85A5
MASHAVTSGQKPHVVCIPFPAQGHINPMLKVAKLLYARGFHVTFVNTNYNHNRLIRS
RGPNSLDGLPSFRFESIPDGLPEENKDVMQDVPTLCESTMKNCLAPFKELLRRINTTK
DVPPVSCIVSDGVMSFTLDAAEELGVPDVLFWTPSACGFLAYLHFYRFIEKGLSPIKDE
SSLDTKINWIPSMKNLGLKDIPSFIRATNTEDIMLNFFVHEADRAKRASAIILNTFDSLE
HDVVRSIQSIIPQVYTIGPLHLFVNRDIDEESDIGQIGTNMWREEMECLDWLDTKSPN
SVVYVNFGSITVMSAKQLVEFAWGLAATKKDFLWVIRPDLVAGDVPMLPPDFLIETA
NRRMLASWCPQEKVLSHPAVGGFLTHSGWNSTLESLSGGVPMVCWPFFAEQQTNC
KYCCDEWEVGMEIGGDVRREEVEELVRELMDGDKGKKMRQKAEEWQRLAEEATKPI
YGSSELNFQMVVDKVLLGE

>Arabidopsis UGT85A3
MGSRFVSNEQKPHVVCVPYPAQGHINPMMKVAKLLHVKGFHVTFVNTVYNHNRLL
RSRGANALDGLPSFQFESIPDGLPETGVDATQDIPALSESTTKNCLVPFKKLLQRIVTR
EDVPPVSCIVSDGSMSFTLDVAEELGVPEIHFWTTSACGFMAYLHFYLFIEKGLCPVK
DASCLTKEYLDTVIDWIPSMNNVKLKDIPSFIRTTNPNDIMLNFVVREACRTKRASAII
LNTFDDLEHDIIQSMQSILPPVYPIGPLHLLVNREIEEDSEIGRMGSNLWKEETECLGW
LNTKSRNSVVYVNFGSITIMTTAQLLEFAWGLAATGKEFLWVMRPDSVAGEEAVIPKE
FLAETADRRMLTSWCPQEKVLSHPAVGGFLTHCGWNSTLESLSCGVPMVCWPFFAE

QQTNCKFSCDEWEVGIEIGGDVKRGEVEAVVRELMDGEKGKKMREKAVEWRRLAEK
ATKLPCGSSVINFETIVNKVLLGKIPNT

>Arabidopsis UGT85A1
MGSQIIHNSQKPHVVCVPYPAQGHINPMMRVAKLLHARGFYVTFVNTVYNHNRFLR
SRGSNALDGLPSFRFESIADGLPETDMDATQDITALCESTMKNCLAPFRELLQRINAG
DNVPPVSCIVSDGCMSFTLDVAEELGVPEVLFWTTSGCAFLAYLHFYLFIEKGLCPLK
DESYLTKEYLEDTVIDFIPTMKNVKLKDIPSFIRTTNPDDVMISFALRETERAKRASAIIL
NTFDDLEHDVVHAMQSILPPVYSVGPLHLLANREIEEGSEIGMMSSNLWKEEMECLD
WLDTKTQNSVIYINFGSITVLSVKQLVEFAWGLAGSGKEFLWVIRPDLVAGEEAMVPP
DFLMETKDRSMLASWCPQEKVLSHPAIGGFLTHCGWNSILESLSCGVPMVCWPFFA
DQQMNCKFCCDEWDVGIEIGGDVKREEVEAVVRELMDGEKGKKMREKAVEWQRLA
EKATEHKLGSSVMNFETVVSKFLLGQKSQD

>Arabidopsis UGT85A4
MEQHGGSSSQKPHAMCIPYPAQGHINPMLKLAKLLHARGFHVTFVNTDYNHRRILQ
SRGPHALNGLPSFRFETIPDGLPWTDVDAKQDMLKLIDSTINNCLAPFKDLILRLNSG
SDIPPVSCIISDASMSFTIDAAEELKIPVVLLWTNSATALILYLHYQKLIEKEIIPLKDSSD
LKKHLETEIDWIPSMKKIKLKDFPDFVTTTNPQDPMISFILHVTGRIKRASAIFINTFEK
LEHNVLLSLRSLLPQIYSVGPFQILENREIDKNSEIRKLGLNLWEEETESLDWLDTKAE
KAVIYVNFGSLTVLTSEQILEFAWGLARSGKEFLWVVRSGMVDGDDSILPAEFLSETK
NRGMLIKGWCSQEKVLSHPAIGGFLTHCGWNSTLESLYAGVPMICWPFFADQLTNR
KFCCEDWGIGMEIGEEVKRERVETVVKELMDGEKGKRLREKVVEWRRLAEEASAPPL
GSSYVNFETVVNKVLTCHTIRST
