# Supplementary material for: Identification of a tomato UDP-arabinosyltransferase for airborne volatile reception
Source: Nat Commun. 2023 Feb 8;14:677. doi: 10.1038/s41467-023-36381-8 (PMC9908901; doi:10.1038/s41467-023-36381-8)
Supplement: Supplementary file 4 — Supplementary Data 2 [file 41467_2023_36381_MOESM4_ESM.pdf]

Supplementary Data 2

Amino acids sequences for Phylogenetic analysis (Supplementary Fig. 5a)

>Sopen11g005580
MAENSKKLHIAVFPWLAFGHMIPYLELSKLIAQKGHKFSFISTPRNIDSLPKLPPSLAPF
LNFVKIPLPHVEKLPKNAEATTDLSYEQVKYLKLAQDELKEPMDKFLEDSAPDFILFD
FTSYWLPSIASKFNIPTGYFSIFVAAYVGFTGPVPGLNNNYEIRMTLEELTVSPKWVPFE
TAVAFKEFELLRIYEGCKEGEEENFYDISRMYKTFENCNFLLVRSCLEFEPEWLKVVEDI
HPKPVIPVGQLPTTSYEDDNTDIDAWREIKLWPDKQEKGKVIYVAFGSEAKLSQNELS
YH

>Sopen11g005590
MAKNSKMLHIGVFPWLAFGHMIPYLELSKQIAQKGHKISFISTPRNIDCLPKLSPNLTP
FLNFVKLPMPYVEKMPENAEATIDVTYEQVKYLKLAQDGLEESMAKFLEDSDLDFILF
DFTSYWVPSIASKFNIPSGHFSIYIAAFLGFTGPVPGLNNDYEIRTTLEEYTVPPKWVPF
ETTVAFKLFEVLRIFEATMKGEEENIADITRYYKSVENCDFLFVRSCSELEPEWLKVLG
DIHRKPVFPLGQLPTTQYEDDSTKIDAWIEIKLWLDKQEKGKVIYVAFGSEAKPSQNE
LIELSLGLELSGLPFLWVLRTKRGESDDELIQLPEGFEDRMKGRGIVCTSWAPQLKILS
HDSVGGFLTHSG

>UGT91A6
METQLVKSIANGDESNTKKLHIVMFPWLAFGHIIPFLELSKFIARKGHKISFISSPRNIDR
LPKIPSEFSNSITFVKIPLAKIDGLPKDAEATIDIITSEEMTYLKKAMDGMEKDVTNFLE
NSCPDWIIQDFAQYWLAPISTRLGISRIFYSIINAWFLSFLGSFENMINTNNCTSPPKLE
DFLVPPKWIPFETKATYRLHEARWMVESSQKNVSGVSDMYRNGVTIEGADAIIIRHCH
EFEGQWLKLLEDLHHMPVLPTGLMPPIVESSSHEKNESWISIKDWLDEKPKGSVVYVA
LGSEVTVGQNEINELARGLELSGSPFFWVLRKSSGLGSIDPIVLPDGFEERTKDRGIVW
KSWAPQLKILSHESVGGLLTHCGWSSIIEGLIFGHPLIMLPFLVDQGLNARILQDKGV
GVEVPRNEEGGTYTSDSVVNSVKLVMVENDGKIIREKAKEMSAIFGNKELHDKYIENL
INFLENQLIDS

>UGT91A7
MANGDENTTKKLHIVMFPWLAFGHIIPFLELSKFLALKGHKISFISTPRNIDRLPKIPSE
FSNSITFVKIPLPKIDGLPKDAEATMDITSEEMTYLKKAMDGMENEVTNFLENNCPD
WIIQDFAQYWLAPISTRLGISRIFYSIINAWFLSFLGSFENMINTNNCTSPPKLEDFLVPP
KWIPFETKATYRLHEARWMVESSQKNVSGVSDMYRNGVTIEGADAIIIRHCHEFEGQ
WLKLLEDLHHMPVLPTGLMPPIVESSSHEKNESWISIKEWLDEKPKGSVVYVALGSEV
TVGQNEINELARGLELSGSPFFWVLRKSSGLGSIDPIVLPDGFEERTKDRGIVWKSWAP
QLKILSHESVGGFLTHCGWSSIIEGLMFGHSLIMLPFLVDQGLNARIIEDKGVGIEVPR

NEEDGSYTSNSVANSVKLVMVKNDGKLIREKAKELSSIFSNKELHDKYIENLINFLEDY
KKSQ

>UGT91A8
METQLVKSIANGDESNTKKLHIVMFPWLAFGHIIPFLELSKFIARKGHKISFISTPRNID
RLPKIPSEFSNSITFVKIPLAKIDGLPKDAEATIDIITSEEMTYLKKAMDGMEKDVTNFL
ENNCPDWIIQDFAQYWLAPISTRLGISRIFYSIINAWFLSFLGSFENMINTNNCTSPPKL
EDFLVPPRWIPFETKAAYRLHEARWMVESSQKNVSGVSDMYRNGVTIEGADAIIIRHC
HEFEGQWLKLLEDLHHMPVLPTGLMPPIVESSSDEKNESWISIKQWLDEKPKGSVVY
VALGSEVTVGQSGINELARGLELSGSPFFWVLRKLSGLGNIDPIVLPDGFEERTKGQGI
VWKSWAPQLKILSHESVGGFLTHCGWSSTIEGLIFGLPLIMLPFLVDQGLNARILRDE
GVGVEVPRNEEGGTYTSDSVVNSVKLVMVENDGKIIREKAKEMSSIFGNKELHDKYIE
NLINFLENHKK

>UGT91A9
MANGDENNTKKLHIVMFPWLAFGHIIPFLELSKFIARKGHKISFISTPRNIDRLPKIPSEF
SNSITFVKIPLAKVDGLPKDAEATMDITSEEIIYLKKAMDGMENEVTNFLENNCPDWII
QDFAQYWLAPISTRLGISRIFYSIINAWFLSFLGSFENMINTNNCTSPPKLEDFLVPPRW
IPFETKAAYRLHEARWMVESSQKNVSGVSDMYRNGVTIEGADAIIIRHCHEFEGQWLK
LLEDLHHMPVLPTGLMPPIVETSSDEKNEFWISIKEWLDEKPKGSVVYVALGSEVTVG
QSEINELASGLELSWSPFFWVLRKPSGSRNKDPIELPDGFEERTKGRGIVWKSWVPQL
KILSHESIGGFLTHCGWSSIIEGLMFGHSLIMLPFLVDQGLNARIIEDKGVGIEVPRNEE
DGSYTSNSVANSVKLAMVNNDGKLIREKAKELSSIFSNKELHDKYIENLINFLEDYKKN
Q

>UGT91R1
MAENGKKLHIAVFPWLAFGHMIPYLELSKLIAQKGHKISFISTPRNIDRLPKLPPNLTPF
LNFVKLPMPHVEKLPENAEATIDLPYEQVKYLKLAQDALQESMSKFIEDSDIDFILFDF
TSYWVPSIASKFNIPSGYFSIFIAAFLGFTGPVPGLNNDYEIRMTPEEYTVTPKWVPFET
TVAFKLFEVSRIFEASMKGEEENIADIVRYYRSVENCDFLLVRSCSEFEPEWLKVVGDIH
RKPVFPVGQLPTTPYEDDSTKIDAWREIKLWLDKQEKGKVIYVAFGSEAKPSQNELTE
LSLGLELSGLPFFWVLRIKRGESDDELIQLPEGFEERTKGRGIVCTSWAPQLKILSHDS
VGGFLTHSGWSSVVEAIQFEKSLVLLTFLADQGINARLLEEKKMAYSIPRNDQDGSFT
RDSVAESLNLVLVKEEGFIYREKIKEMKDLFCDKKRQNNYVENLLSFLQDYEKIKA

>UGT91R2

MAENGKKLHIAVFPWLAFGHMIPYLELSKLIAQKGHKISFISTPRNIDRLPKLPPSLIPFF
NFVKLPMPHVEKLPENAEATIDLPYEQVKHLKLAHDALQESIAKFLEDSDIDFILFDFV
SYWLPSIASKFNIPTGYFSIFVAAYLGFTGPVPGLNNNYEIRMTLEELTVSPKWVPFETA
VAFKEFELLRIYEGCKEGEEENFYDISRMYKTFENCDFLLVRSCLEFEPEWLKVVEDIH
PKPVIPVGQLPTTSYEDDNTDIDAWREIKLWLDKQEKGKVIYVAFGSEAKLSQNELTE
LSLGLELSGLPFFWVLRTKRGESDNELIQLPEGFGERTKERGIVYTSWVPQLKILSHDS
VGGFLTHAGWSSIVEAIQFEKPLLLLTFLADQGINARLLEEKKVAYLIPRNDWDGSFT
HNAVVESLYLVLLEKEGEIYRKKIKEVKNLCCDKKRQDDYVENLLRFLQNYKKIKV

>UGT91R3
MAENSKKLHIAVFPWLAFGHMIPYLELSKLIAQKGHKISFISTPRNIDRLPKLPPSLAPF
LNFVKIPLPYVEKLPKNAEATTDLPYEQVKYLKLAHDALKEPMAKFLEDSAPDFILFDF
TSYWLPSIASKFNIPTGYFSIFVAAYLGFTGPVPGLNNNYENRMTLEELTVSPKWVPFE
TAVAFKEFELLRIYEGCKEGEEENFYDISRMYKTFENCDFLLVRSCLEFEPEWLKVVEDI
HPKPVIPVGQLPTTSYEDDNTDIDAWREIKLWLDKQEKGKVIYVAFGSEAKLSQNELT
ELSLGLELSGLPFFWVLRTKRGESDNELIQVPEGFEERTKERGIVYTSWVPQLKILSHD
SVGGFLTHAGWSSIVEAIQFEKPLLLLTFLADQGINARLLEEKKVAYLIPRNDWDGSFT
HKAVVESLYLVLLEKEGEIYQKKIKEVKNLCCDKKRQDDYVENLIRFLQNYKKIKV

>UGT91R4
MAENGKKLHIAVFPWLAFGHMIPYLELSKLIAQKGHIISFISTPRNIDRLPKLPPNLTPF
FNFVKLPMPHVEKLPENAEATIDLPYEQVKYLKLAHDALQESMAKFLEDSDIDFILFD
FASYWIPSIASKFNIPTGYFSIFIAAVLGFIGSEPGLNNDYQIRKTPEEYTVSPNWVPFET
TVAFKLFEVSRIFEASMKGEEDNVSDIIRMYKGLRYSDFLLVRSCSEFEPEWLKVVGDI
HRKPVFPVGQLPTTPYEDDSTKIDAWREIKLWLDKQEKGKVIYVAFGSEAKPSQNEL
TELSLGLELSGLPFFWVLRIKRGESDDELIQLPEGFEERTKGRGIVYTSWAPQLKILSH
DSVGGFLTHSGWSSVVEAIQFEKSLVLLTFLADQGINARLLEEKKMAYSIPRNDQDGS
FTRHSVAESLKLVLTKKEGFIYREKIKEMKNLFCDKERQNNYVENLLSFLQNYEKIKA
