# Supplementary material for: Identification of a tomato UDP-arabinosyltransferase for airborne volatile reception
Source: Nat Commun. 2023 Feb 8;14:677. doi: 10.1038/s41467-023-36381-8 (PMC9908901; doi:10.1038/s41467-023-36381-8)
Supplement: Supplementary file 5 — Supplementary Data 3 [file 41467_2023_36381_MOESM5_ESM.pdf]

Supplementary Data 3

Synthesis of (*Z*)-3-hexenyl β-vicianoside (HexVic) (Supplementary Fig. 10)

General methods

Synthesis of *p*-methoxyphenyl (2,3,4-tri-*O*-Benzoyl-α-L-arabinopyranosyl) -(1→6)-2,3-di-*O*-benzyl-β-D-glucopyranoside (S3)

Synthesis of *p*-methoxyphenyl (2,3,4-tri-*O*-Benzoyl-α-L-arabinopyranosyl)-(1→6)-2,3-di-*O*-benzyl-4-*O*-acetyl-β-D-glucopyranoside (S4)

Synthesis of (2,3,4-tri-*O*-Benzoyl-α-L-arabinopyranosyl)-(1→6)-2,3-di-*O*-benzyl-4-*O*-acetyl-D-glucopyranosyl 2,2,2-trifluoro-*N*-phenyl-acetimidate (S5)

Synthesis of 3-*cis*-hexenyl (2,3,4-tri-*O*-Benzoyl-α-L-arabinopyranosyl)-(1→6)-2,3-di-*O*-benzyl-4-*O*-acetyl-β-D-glucopyranoside (S6)

Supplementary references

General methods

All chemicals were reagent grade and purchased from Nacalai tesque. Unless otherwise noted, all chemicals and solvents were used as supplied. Molecular sieves were activated oil bath at 160°C for 2h, followed by being dried *in vacuo*. Moisture sensitive reactions were performed under argon atmosphere. All reactions were monitored by TLC on Silica Gel 60 F_{254} precoated glass slides (Merck). Detection was performed by examination under UV light (254 nm) and/or by charring with 5% 12molybdo(VI)phosphoric acid in EtOH and/or anisaldehyde-H_2SO_4 staining. Flash column chromatography was performed on silica gel (Nacalai, Silica Gel 60 mesh 230-400). Preparative TLC (PLC) was performed on Silica Gel 60 plate F_{254} (Merck). ^{1}H and ^{13}C NMR spectra were recorded at 298 K on Bruker AVANCE III HD 400 spectrometer. Chemical shifts are reported in ppm relative to the solvent peak. High-resolution ESI-MS was carried out in positive and negative mode on Shimadzu LCMS-IT-TOF-MS spectrometer with aqueous MeOH containing 0.1 % HCO_2H as the mobile phase and sodium trifluoroacetate as an external standard.

***p*-methoxyphenyl (2,3,4-tri-*O*-Benzoyl-α-L-arabinopyranosyl) -(1→6)-2,3-di-*O*-benzyl-β-D-glucopyranoside** (S3)

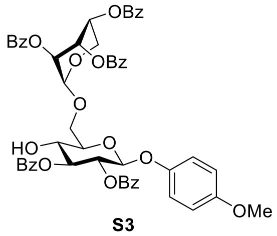

S3

A solution of *p*-methoxyphenyl 2,3-di-*O*-benzyl-*β*-D-glucopyranoside (**S2**)[1] (1.40 g, 2.84 mmol), 2,3,4-tri-O-Benzoyl-L-arabinopyranosyl (*N*-phenyl)trifluoroacetimidate (**S1**)[2] (1.80 g, 2.84 mmol) and MS 4Å (3.20 g) in anhydrous CH_2Cl_2 (28 mL) was stirred at room temperature for 0.5 h. TfOH (24 μL, 0.284 mmol) was added to the mixture and the mixture was stirred at −40 °C for 2 h. The reaction mixture was quenched with Et_3N and filtered through the Celite with glass filter, then the filtrate was concentrated *in vacuo*. The obtained crude product was purified by flash column chromatography (hexane : AcOEt = 2 : 1 → 3 : 2) to give the desired product **S3** (1.99 g, 74%); ^{1}H NMR (400 MHz, $CDCl_3$): δ 8.05-7.93 (10H, m), 7.59-7.26 (15H, m), 6.91(2H, d, *J* = 8.5 Hz), 6.74 (2H, d, *J* = 8.5 Hz), 5.75-5.57 (4H, m), 5.43-5.39 (1H, m), 5.07 (1H, d, *J* = 7.9. Hz), 4.92 (1H, d, *J* = 5.4 Hz), 4.37 (1H, dd, *J* = 4.6, 12.5 Hz), 4.31 (1H, d, *J* = 11.0 Hz), 4.01-3.90 (3H, m), 3.77-3.75 (1H, m), 3.70 (3H, s); ^{13}C NMR (100 MHz, $CDCl_3$): δ 167.50, 165.82, 165.73, 165.42, 165.32, 155.86, 151.31, 133.70, 133.60, 133.56, 133.51, 133.38, 130.17, 130.02, 129.86, 129.45, 129.43, 129.24, 129.18, 128.98, 128.65, 128.55, 119.16, 114.66, 101.21, 100.81, 77.06, 75.57, 71.51, 70.24, 70.05, 69.65, 68.23, 68.12, 62.08, 55.70; HRMS (IT-TOF, $[M+NH^4]^+$) calcd for $C_{53}H_{50}NO_{16}$ 956.3124, found

956.3033.

***p*-methoxyphenyl (2,3,4-tri-*O*-Benzoyl-α-L-arabinopyranosyl)-(1→6)-2,3-di-*O*-benzyl-4-*O*-acetyl-β-D-glucopyranoside** (S4)

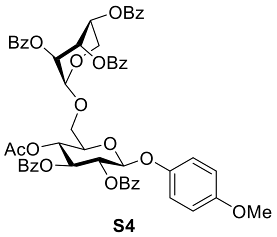

S4

A solution of **S3** (1.99 g, 2.12 mmol), and DMAP (25.9 mg) in pyridine (10 mL) was stirred at room temperature. Acetyl chloride (0.18 mL, 2.54 mmol) was added to the mixture and the mixture was stirred at room temperature for 2.5 h. The reaction mixture was concentrated *in vacuo*. The obtained crude product was purified by flash column chromatography (hexane : AcOEt = 3 : 1 → 2 : 1) to give the desired product **S4** (2.03 g, 98%); ^{1}H NMR (400 MHz, $CDCl_3$): δ 8.05-7.87 (10H, m), 7.59-7.34 (15H, m), 6.89 (2H, d, J = 8.7 Hz), 6.73 (2H, d, J = 8.7 Hz), 5.71-5.55 (5H, m), 5.26 (1H, t, J = 9.6 Hz), 5.06 (1H, d, J = 7.7. Hz), 4.90 (1H, d, J = 5.4 Hz), 4.35 (1H, dd, J = 4.9, 12.5 Hz), 4.07-3.82 (4H, m), 3.70 (3H, s), 1.95 (3H, s); ^{13}C NMR (100 MHz, $CDCl_3$): δ 169.65, 165.90, 165.81, 165.68, 165.38, 165.16, 155.98, 151.20, 133.62, 133.57, 133.47, 133.42, 130.02, 130.00, 129.93, 129.91, 129.52, 129.37, 129.35, 129.27, 128.93, 128.66, 128.57, 128.55, 119.14, 114.74, 101.22, 100.35, 74.06, 73.12, 71.85, 70.27, 69.89, 68.99, 68.16, 67.80, 61.99, 55.72, 20.72; HRMS (IT-TOF, $[M+NH^4]^+$) calcd for $C_{55}H_{52}NO_{17}$ 998.3230, found 998.3125.

(2,3,4-tri-*O*-Benzoyl-α-L-arabinopyranosyl)-(1→6)-2,3-di-*O*-benzyl-4-*O*-acetyl-D-glucopyranosyl 2,2,2-trifluoro-*N*-phenyl-acetimidate (S5)

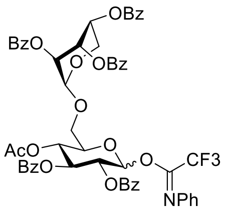

S5

A solution of **S4** (1.92 g, 1.96 mmol) in MeCN (12 mL) and H_2O (6.0 mL) was stirred at room temperature. CAN (3.22 g, 5.87 mmol) was added to the mixture and the mixture was stirred at room temperature for 30 min. The mixture was extracted with AcOEt and the combined organic phase was washed with sat. $NaHCO_3$ aq., H_2O and brine and dried over $MgSO_4$. The obtained crude product was purified by flash column chromatography (hexane : AcOEt = 2 : 1 → 1 : 1) to give the product. A

solution of the obtained product and K_2CO_3 (542 mg, 3.92 mmol) in Acetone (20 mL) was stirred. (Z)-2,2,2-trifluoro-N-phenylacetimidoyl chloride (370 µL, 2.35mmol) was added to the mixture and the mixture was stirred at room temperature for overnight. The reaction mixture was filtered through the Celite with glass filter, then the filtrate was concentrated *in vacuo*. The obtained crude product was purified by flash column chromatography (hexane : AcOEt = 2 : 1) to give the desired product **S5** (1.41 g, 68% in 2 steps); ^{1}H NMR (400 MHz, $CDCl_3$): δ 8.08-8.02 (6H, m), 7.98-7.94 (2H, m), 7.89-7.85 (2H, m), 7.58-7.50 (6H, m), 7.46-7.35 (10H, m), 7.24-6.97 (2H, m), 6.77-6.65 (2H, br), 6.40-6.23 (1H, br), 5.96 (1H, t, J = 9.9 Hz), 5.73-5.65 (3H, m), 5.50-5.25 (2H, m), 4.86-4.85 (1H, m), 4.40-4.33 (2H, m), 4.10-4.06 (1H, m), 3.95-3.90 (1H, m), 3.80 (1H, dd, J = 4.4, 11,4 Hz), 1.94 -1.93(3H, m) ; ^{13}C NMR (100 MHz, $CDCl_3$): δ171.30, 169.36, 165.79, 165.70, 165.66, 165.33, 165.30, 165.27, 164.80, 142.95, 133.78, 133.69, 133.63, 133.56, 133.48, 130.07, 130.03, 129.97, 129.49, 129.44, 129.39, 129.35, 129.05, 129.02, 128.95, 128.80, 128.73, 128.68, 128.65, 128.62, 128.59, 124.76, 124.38, 119.36, 119.20, 100.45, 100.12, 92.36, 74.41, 72.81, 71.73, 71.10, 70.63, 70.31, 70.03, 69.90, 69.77, 69.71, 68.35, 67.89, 67.44, 67.26, 61.57, 20.64, 20.62; HRMS (IT-TOF, $[M+AcO]^-$) calcd for $C_{58}H_{49}NO_{18}$ 1104.2907, found 1104.2785.

3-*cis*-hexenyl (2,3,4-tri-*O*-Benzoyl-α-L-arabinopyranosyl)-(1→6)-2,3-di-*O*-benzyl-4-*O*-acetyl-β-D-glucopyranoside (S6)

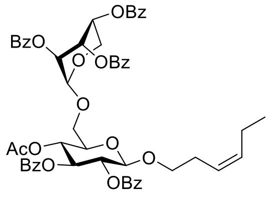

S6

A solution of **S5** (210 mg, 0.20 mmol), MS 4Å (0.25 g) and (Z)-hex-3en-1-ol (28 µL, 0.24 mmol) in DCM (4 mL) was stirred at −20 °C. TfOH (1.78 µL, 20 µmol) was added to the mixture and the mixture was stirred at 0 °C for 2.5 h. The reaction mixture was quenched with Et_3N and filtered through the membrane filter with glass filter, then the filtrate was concentrated *in vacuo*. The obtained crude product was purified by flash column chromatography (toluene : AcOEt = 10 : 1 → 8 : 1) to give the desired product **S6** (110 mg, 57%); ^{1}H NMR (400 MHz, $CDCl_3$): δ 8.06-8.01 (4H, m), 7.97 (2H, d, J = 7.6 Hz), 7.91 (2H, d, J = 7.7 Hz), 7.86 (2H, d, J = 7.7 Hz), 7.60-7.33 (15H, m), 5.73-5.70 (2H, m), 5.64-5.58 (2H, m), 5.31 (1H, dd, J = 9.4, 8.3 Hz), 5.20-5.13 (2H, m), 5.08-5.02 (1H, m), 4.86 (1H, d, J = 5.7 Hz), 4.60 (1H, d, J = 7.8 Hz), 4.36 (1H, dd, J = 4.2, 12.7 Hz), 4.04 (1H, d, J = 11.0 Hz), 3.09-3.85 (2H, m), 3.77-3.72 (1H, m), 3.64-3.59 (1H, m), 3.25-3.19 (1H, m), 2.09-2.02 (1H, m), 1.93 (3H, s), 1.88-1.81 (2H, m), 0.82 (3H, t, J = 7.5 Hz); ^{13}C NMR (100 MHz, $CDCl_3$): δ 171.02, 167.19, 167.08, 166.97, 166.60, 166.38, 135.06, 134.84, 134.80, 134.74, 134.47, 131.29, 131.28, 131.23, 131.15,

130.88, 130.80, 130.67, 130.52, 130.29, 129.91, 129.80, 129.67, 125.69, 102.40, 102.08, 74.83, 74.54, 73.19, 71.64, 71.23, 71.01, 70.63, 69.67, 69.60, 63.57, 28.76, 22.00, 21.88, 15.51; HRMS (IT-TOF, $[M+AcO]^-$) calcd for $C_{56}H_{55}O_{18}$ 1015.3394, found 1015.3290.

(*Z*)-3-hexenyl β-vicianoside (HexVic) [3]

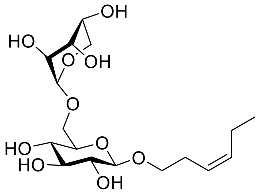

(*Z*)-3-hexenyl β-vicianoside (HexVic)

A solution of **S6** (110 mg, 0.11 mmol) in MeOH (2 mL) and THF (1 mL) was stirred at room temperature. Sodium methoxide 28% in MeOH (2.4 μL, 11 μmol) was added to the mixture and the mixture was stirred at room temperature for overnight. The reaction mixture was quenched with DOWEX(CO_2H) and filtered through the membrane filter with glass filter, then the filtrate was concentrated *in vacuo*. The obtained crude product was purified by flash column chromatography ($CHCl_3$: MeOH = 3 : 1 → 1 : 1) and preparative HPLC (Triart C18 column, H_2O : 90% MeCN = 90 : 10 to 0 : 100, 20 min) to give the desired product **(*Z*)-3-hexenyl β-vicianoside (HexVic)** (32.1 mg, 71%) ^{1}H NMR (400 MHz, CD_3OD): δ 5.50-5.37 (2H, m), 4.33 (1H, d, J = 6.7 Hz), 4.29 (1H, d, J = 7.8 Hz), 4.11 (1H, dd, J = 11.3, 2.2 Hz), 3.90-3.81 (3H, m), 3.75 (1H, dd, J = 11.3, 5.5 Hz), 3.63-3.53 (4H, m), 3.48-3.43 (1H, m), 3.39-3.33 (2H, m), 3.32-3.18 (1H, m), 2.42-2.37 (2H, m), 2.13-2.06 (2H, m), 0.99 (3H, t, J = 7.5 Hz); ^{13}C NMR (100 MHz, CD_3OD): δ 134.50, 125.91, 105.13, 104.36, 77.92, 76.85, 75.04, 74.19, 72.37, 71.55, 70.61, 69.49, 69.46, 66.73, 28.81, 21.56, 14.66; HRMS (IT-TOF, $[M+NH^4]^+$) calcd for $C_{17}H_{34}NO_{10}$ 412.2177, found 412.2199.

Supplementary references

1. Gu, G. et al. Synthesis and Immunological Characterization of Modified Hyaluronic Acid Hexasaccharide Conjugates. *J. Org. Chem.* **78**, 8004−8019 (2013)
2. Yu, B., & Tao, H. Glycosyl Trifluoroacetimidates. 2. Synthesis of Dioscin and Xiebai Saponin I. *J. Org. Chem.* **67**, 9099-9102 (2002)
3. Kishida, M., Fujii, M., Ida, Y., & Akita H. Chemoenzymatic Synthesis of Naturally Occurring (Z)-3-Hexenyl 6-O-Glycosyl-β-D-glucopyranosides. *Heterocycles* **65**, 2127-2137 (2005).
