# Supplementary material for: Identification of a tomato UDP-arabinosyltransferase for airborne volatile reception
Source: Nat Commun. 2023 Feb 8;14:677. doi: 10.1038/s41467-023-36381-8 (PMC9908901; doi:10.1038/s41467-023-36381-8)
Supplement: Supplementary file 6 — Reporting Summary [file 41467_2023_36381_MOESM6_ESM.pdf]

nature portfolio

Corresponding author(s): Junji Takabayashi, Toshiyuki Ohnishi

Last updated by author(s): Jan 18, 2023

Reporting Summary

Nature Portfolio wishes to improve the reproducibility of the work that we publish. This form provides structure for consistency and transparency in reporting. For further information on Nature Portfolio policies, see our Editorial Policies and the Editorial Policy Checklist.

Statistics

For all statistical analyses, confirm that the following items are present in the figure legend, table legend, main text, or Methods section.

n/a	Confirmed	
☐	☒	The exact sample size (*n*) for each experimental group/condition, given as a discrete number and unit of measurement
☐	☒	A statement on whether measurements were taken from distinct samples or whether the same sample was measured repeatedly
☐	☒	The statistical test(s) used AND whether they are one- or two-sided<br>*Only common tests should be described solely by name; describe more complex techniques in the Methods section.*
☒	☐	A description of all covariates tested
☒	☐	A description of any assumptions or corrections, such as tests of normality and adjustment for multiple comparisons
☐	☒	A full description of the statistical parameters including central tendency (e.g. means) or other basic estimates (e.g. regression coefficient) AND variation (e.g. standard deviation) or associated estimates of uncertainty (e.g. confidence intervals)
☐	☒	For null hypothesis testing, the test statistic (e.g. *F*, *t*, *r*) with confidence intervals, effect sizes, degrees of freedom and *P* value noted<br>*Give P values as exact values whenever suitable.*
☒	☐	For Bayesian analysis, information on the choice of priors and Markov chain Monte Carlo settings
☒	☐	For hierarchical and complex designs, identification of the appropriate level for tests and full reporting of outcomes
☒	☐	Estimates of effect sizes (e.g. Cohen's *d*, Pearson's *r*), indicating how they were calculated

Our web collection on statistics for biologists contains articles on many of the points above.

Software and code

Policy information about availability of computer code

Data collection	LCMS-2020 system (Shimadzu) and LabSolutions LCMS ver.5.70; Shimadzu) were used for qualitative and quantitative analysis of compounds. StepOne Real-time PCR system (Applied Biosystems/ Thermo Fisher Scientific) and StepOne Software (ver. 2.3; Applied Biosystems/ Thermo Fisher Scientific) were used gene expression level analysis.
Data analysis	GraphPad Prism (version 9.4.1) R (ver 3.3.0) was used for data visualization and for statistical tests. Clustal W and MEGA6 are used for phylogenic analysis of protein sequences.

For manuscripts utilizing custom algorithms or software that are central to the research but not yet described in published literature, software must be made available to editors and reviewers. We strongly encourage code deposition in a community repository (e.g. GitHub). See the Nature Portfolio guidelines for submitting code & software for further information.

Data

Policy information about availability of data

All manuscripts must include a data availability statement. This statement should provide the following information, where applicable:

- Accession codes, unique identifiers, or web links for publicly available datasets
- A description of any restrictions on data availability
- For clinical datasets or third party data, please ensure that the statement adheres to our policy

The nucleotide sequence of UGT91R1 (Solanum lycopersicum cDNA, clone: LEFL1056CC10, HTC in leaf) is available from NCBI as AK323431.
(Z)-3-hexenyl vicianoside (PubChem CID 11560275) and (Z)-3-hexenyl primeveroside (PubChem CID 131751183) are assigned PubChem CID numbers.

Human research participants

Policy information about studies involving human research participants and Sex and Gender in Research.

Reporting on sex and gender	Our study does not include any studies involving animals and human research participants.
Population characteristics	Our study does not include any studies involving animals and human research participants.
Recruitment	Our study does not include any studies involving animals and human research participants.
Ethics oversight	Our study does not include any studies involving animals and human research participants.

Note that full information on the approval of the study protocol must also be provided in the manuscript.

Field-specific reporting

Please select the one below that is the best fit for your research. If you are not sure, read the appropriate sections before making your selection.

- [x] Life sciences
- [ ] Behavioural & social sciences
- [ ] Ecological, evolutionary & environmental sciences

For a reference copy of the document with all sections, see nature.com/documents/nr-reporting-summary-flat.pdf

Life sciences study design

All studies must disclose on these points even when the disclosure is negative.

Sample size	Standard sample sizes in our field were chosen.
Data exclusions	No data were excluded from the analysis
Replication	Details of the technical replicates used in the various experiments are provided in the Methods section and, where appropriate, in the Figure and Supplementary Figure legends.
Randomization	Not applicable to our study. Due to the nature of the experimental step, binding/randomization did not apply to this study.
Blinding	Binding was not relevant for our study.

Reporting for specific materials, systems and methods

We require information from authors about some types of materials, experimental systems and methods used in many studies. Here, indicate whether each material, system or method listed is relevant to your study. If you are not sure if a list item applies to your research, read the appropriate section before selecting a response.

Materials & experimental systems

n/a	Involved in the study
☒	☐ Antibodies
☒	☐ Eukaryotic cell lines
☒	☐ Palaeontology and archaeology
☒	☐ Animals and other organisms
☒	☐ Clinical data
☒	☐ Dual use research of concern

Methods

n/a	Involved in the study
☒	☐ ChIP-seq
☒	☐ Flow cytometry
☒	☐ MRI-based neuroimaging
